# One-pot regioselective C–H activation iodination–cyanation of 2,4-diarylquinazolines using malononitrile as a cyano source†

**DOI:** 10.1039/c9ra02979f

**Published:** 2019-06-11

**Authors:** Ziqiao Yan, Banlai Ouyang, Xunchun Mao, Wei Gao, Zhihong Deng, Yiyuan Peng

**Affiliations:** Key Laboratory of Functional Small Organic Molecule, Ministry of Education, Jiangxi Province's Key Laboratory of Green Chemistry, Jiangxi Normal University Nanchang Jiangxi 330022 China yypeng@jxnu.edu.cn; Department of Chemistry, Nanchang Normal University Nanchang 330032 China

## Abstract

A one-pot cyanation of 2,4-arylquinazoline with NIS and malononitrile has been developed. The one-pot reaction includes two steps. The Rh-catalyzed selective C–H activation/iodization of 2,4-diarylquinazoline with NIS, and then Cu-catalyzed cyanation of the corresponding iodinated intermediate with malononitrile to selectively give 2-(2-cyanoaryl)-4-arylquinazolines or 2-(2,6-dicyanoaryl)-4-arylquinazolines in good to excellent yields.

## Abstract 2

A one-pot cyanation of 2,4-arylquinazoline with NIS and malononitrile has been developed.

## Graphical abstract

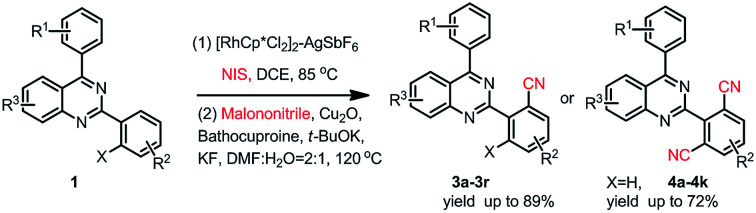

## Introduction

Aromatic nitriles have broad applications in agrochemicals, pharmaceuticals and materials science. The nitrile moiety also serves as a pivotal precursor for a multitude of conversions into a great number of other functional groups, such as hydrolysis into carboxyl or amide, reduction into aldehydes or amines, cycloaddition into heterocycles, *etc.*^1^ Consequently, a number of methods have been developed to introduce a cyanogen group to an aromatic ring. Various catalytic systems and cyanating agents have been established. Of these transformations, transition-metal-catalyzed cyanation of aryl halides^2*a*^ or aromatic C–H bond activation/cyanation was an elegant route to arylnitriles.^2*b*^ Cu(CN)_2_,^3^ Zn(CN)_2_,^4^ NaCN and KCN,^5^ CuSCN,^6^ cyanogen halides,^7^ TMSCN,^8^ NaN_3_,^9^ and K_4_[Fe(CN)_6_]^10^ have been used as cyanation agents to introduce CN into organic molecules. Organic cyanide sources and their combined cyano-groups, such as CH_3_NO_2_,^11^ acetone cyanohydrin,^12^*N*-cyano-*N*-phenyl-*p*-toluenesulfonamide (NCTS),^13^ aryl(cyano)iodonium triflates,^14^ amine/DMSO,^15^ DMF,^16^ formamide,^16*f*,16*g*^ ethyl cyanoacetate,^17^ ethyl(ethoxymethylene)cyanoacetate,^18^ benzyl cyanide,^19^*tert*-butyl nitrite (TBN),^20^ isocyanides,^21^ and acetonitrile^22^ have been used in cyanation for more solubility in organic solvents.

**Scheme 1:**
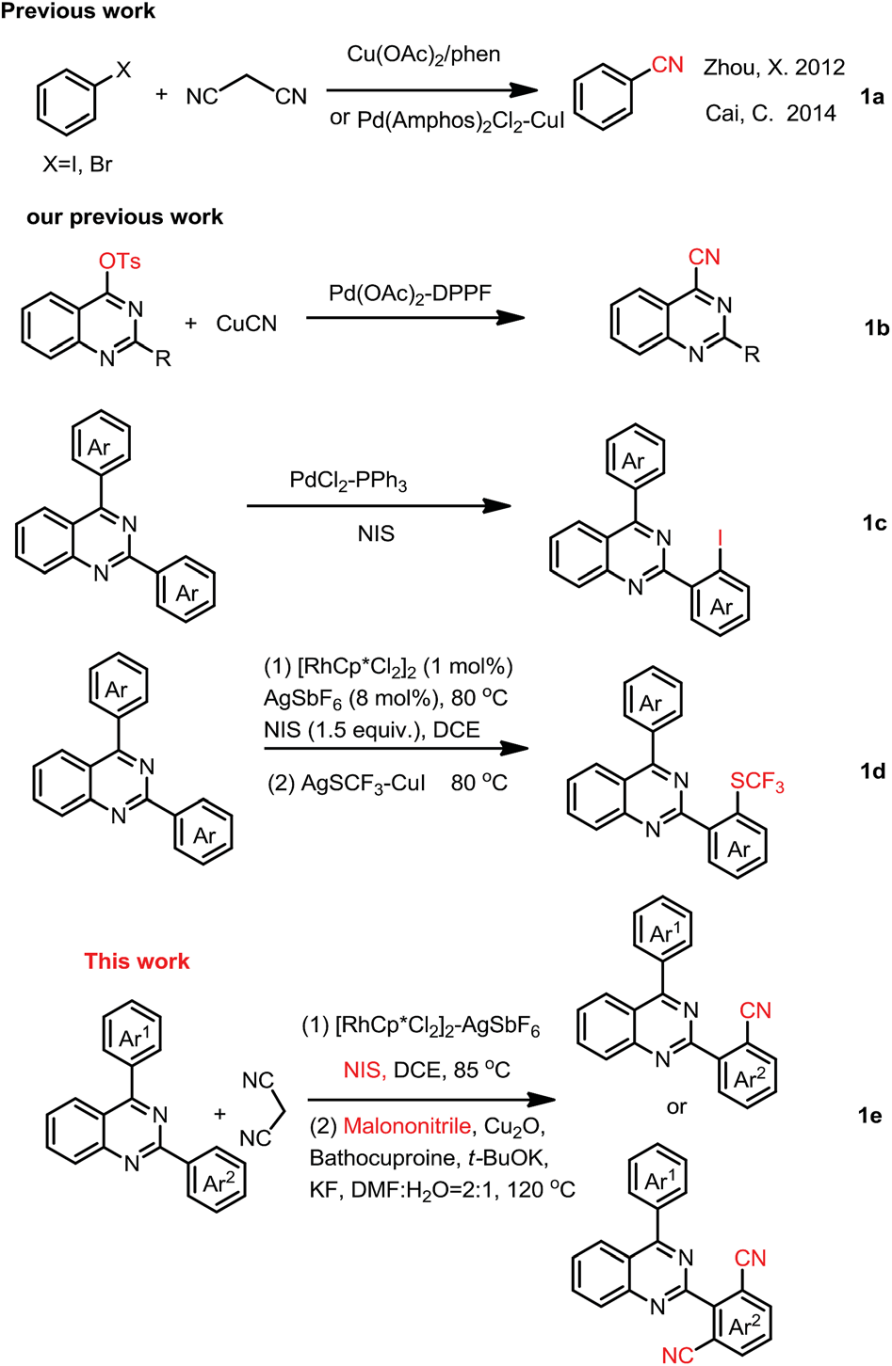
Some previous works and this work.

Recent, malononitrile, being more inexpensive, readily industry available, less toxic, stable and easy-to-handle, had been used as an alternative organic cyano-group source.^23^ Although attempts have been made to utilize malononitrile as the cyano-group source in several aryl halogens cyanation reactions (Scheme 1a),^23^ there is no report about utilizing it as a cyano-group source in one-pot C–H bond activation/cyanation.

**Fig. 1:**
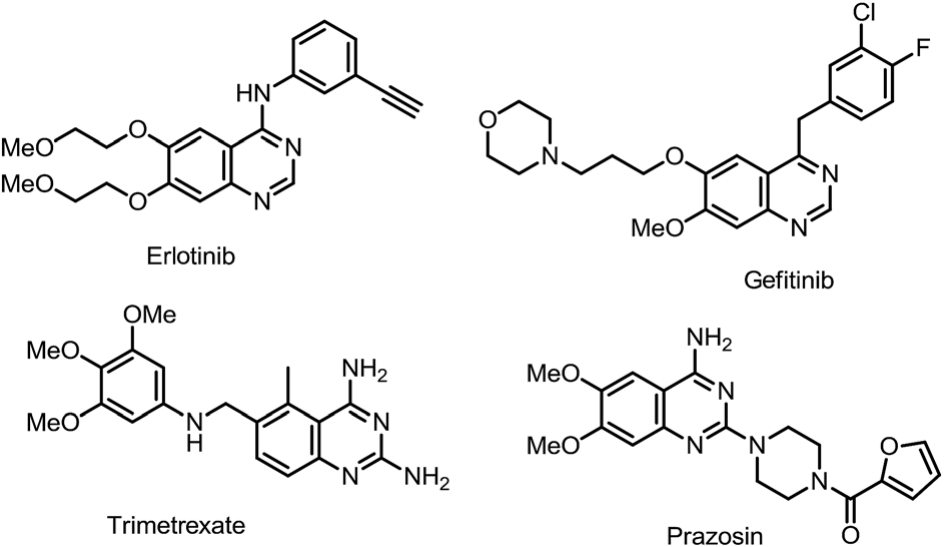
The quinazoline core structure of drugs.

On the other hand, quinazoline core structure exhibits a wide range of important potential biological activities.^24,25^ For example, Erlotinib and Gefitinib are well-known lung cancer drugs.^26^ Prazosin is used for curing high blood pressure^27^ and Trimetrexate has been used in the treatment of pneumocystis pneumonia^28^ (Fig. 1). Thus tremendous efforts have been devoted to develop new synthetic methods for the construction of diverse quinazoline architectures and evaluate their bioactivities.^29^ In the past few years, our group has focused extensively on the development methods for the synthesis of quinazoline skeletons and the late-stage functionalization of the quinazoline core with a wish to construct a quinazoline-based molecular library for bioactivity assay.^30^

However, the construction of quinazoline core coupling-nitrile moiety had been rarely reported. In 2013, we reported palladium catalyzed cyanation of quinazoline-4-tosylates with CuCN for access to quinazoline-4-nitriles (Scheme 1b).^31^ In 2017, we disclosed quinazoline-directed selective *ortho*-iodination of 2,4-di-arylquinazolines for the synthesis of 2-(2-iodoaryl)-4-arylquinazolines (Scheme 1c).^32^ Very recently, iodination of 2,4-diarylquinazoline with NIS catalyzed by rhodium, and then trifluoromethylthiolation of the corresponding iodides for the synthesis of SCF_3_-substituted 2,4-diarylquinazolines was reported in our group (Scheme 1d).^33^ Base on these, we here reported selective synthesis of 2-(2-cyanophenyl)-4-phenylquinazolines and 2-(2,4-dicyanophenyl)-4-phenylquinazolines by one-pot C–H activation iodination/cyanation using malononitrile as a cyano-group source (Scheme 1e).

## Results and discussion

Our interest in this particular on the regioselective C–H activation/cyanation of 2,4-diarylquinazolines originated from our recent studies that [RhCp*Cl_2_]_2_/AgSbF_6_ catalyzed iodination of 2,4-arylquinazoline.^33^ Here, we first optimize the reaction conditions of one-pot C–H iodination/cyanation of 2,4-phenylquinazoline. To avoid produce mixture of mono- and bis-functionalization products, 2-(*o*-methyl)phenyl-4-(*p*-methyl)phenyl quinazoline 1a, which one of *ortho* position was blocked by a methyl, was chosen as the model substrate for the optimization of the one-pot cyanation conditions. According to our previous work,^33^ the standard iodination protocol was carried out by using 1a (1.0 eq.), NIS (*N*-iodosuccinimide, 1.5 eq.), [RhCp*Cl_2_]_2_ (1.0 mol%) and AgSbF_6_ (8.0 mol%) in DCE at 85 °C for 30 min. When the iodination reaction was finished, the DCE solvent was removed under reduced pressure, and the mixture of malononitrile (2.0 eq.), catalyst-system Cu_2_O (10 mol%)–L (20 mol%), *t*-BuOK (2.0 eq.) and DMF (2.0 mL) as the solvent were added and then reacted at 120 °C. To our delight, the target product 3a was isolated in a yield of 25% (Table 1, entry 1). Various of bases, such as K_2_CO_3_, KOH, Cs_2_CO_3_, and NaOH, were screened, and *t*-BuOK was found to be the best one (entries 1–5). Different copper salts were then examined, and Cu_2_O was proved to be the best selected, which resulted in a yield of 58% (entries 6–12). The results of solvents tested indicated that trace amount of product was detected in H_2_O or acetone (not listed in Table 1); low yield of 47% and 49% were obtained in DMAC or DMSO (entries 13 and 14). Additive KF found to be beneficial to the reaction, and gave a yield up to 63% (entry 15). Among the ligands used, L_4_ (Bathocuproine) was found to be more facilitating to the reaction than the others (entries 16–18). Excitingly, adding 50 v% of water in DMF can improve the yield to 84% (entries 19–21) and short the reaction time from 18 h to 3 h. Finally, when NBS (*N*-bromosuccinimide) was used as the reactant instead of NIS under the standard reaction conditions, only trace amount of the desired product was detected.

**Table 1:** Optimization of the reaction conditions^a^^,^^b^

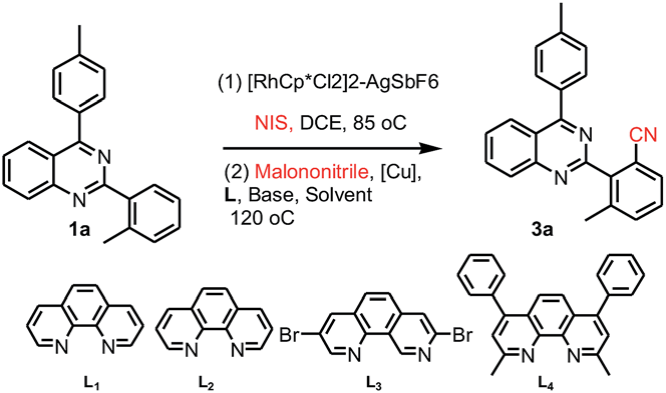					
Entry	[Cu]	L	Base	Solvent	Yield^c^
1	Cu(OAc)_2_	L_1_	*t*-BuOK	DMF	25
2	Cu(OAc)_2_	L_1_	NaOH	DMF	16
3	Cu(OAc)_2_	L_1_	K_2_CO_3_	DMF	21
4	Cu(OAc)_2_	L_1_	KOH	DMF	15
5	Cu(OAc)_2_	L_1_	Cs_2_CO_3_	DMF	18
6	CuCl	L_1_	*t*-BuOK	DMF	32
7	CuCO_3_	L_1_	*t*-BuOK	DMF	24
8	Cu(TFA)_2_	L_1_	*t*-BuOK	DMF	26
9	CuBr	L_1_	*t*-BuOK	DMF	48
10	CuSO_4_	L_1_	*t*-BuOK	DMF	45
11	Cu_2_O	L_1_	*t*-BuOK	DMF	58
12	CuI	L_1_	*t*-BuOK	DMF	44
13	Cu_2_O	L_1_	*t*-BuOK	DMAC	47
14	Cu_2_O	L_1_	*t*-BuOK	DMSO	49
15	Cu_2_O	L_1_	*t*-BuOK–KF	DMF	63
16	Cu_2_O	L_2_	*t*-BuOK–KF	DMF	33
17	Cu_2_O	L_3_	*t*-BuOK–KF	DMF	57
18	Cu_2_O	L_4_	*t*-BuOK–KF	DMF	69
**19**	**Cu**_**2**_ **O**	L_4_	***t*-BuOK–KF**	**DMF** **:** **H**_**2**_**O = 2** **:** **1**	**84**
20	Cu_2_O	L_4_	*t*-BuOK–KF	DMF : H_2_O = 2 : 0.8	71
21	Cu_2_O	L_4_	*t*-BuOK–KF	DMF : H_2_O = 2 : 0.6	75
22^d^	Cu_2_O	L_4_	*t*-BuOK–KF	DMF : H_2_O = 2 : 0.6	Trace

a Optimized conditions are denoted in bold.

b Reaction conditions: 1a (0.1 mmol), NIS (1.5 eq.). [RhCp*Cl_2_]_2_ (1.0 mol%)–AgSbF_6_ (8 mol%), malononitrile (2.0 eq.) [Cu] (10 mol%)–ligand (20 mol%), base (2.0 eq.), in solvent (2.0 mL) at 120 °C.

c Isolated yield.

d NIS was replaced by NBS.

We then started to investigate the scope and the generality of this reaction under optimized conditions [NIS (1.5 eq.), [RhCp*Cl_2_]_2_ (1.0%), AgSbF_6_ (8.0 mol%), malononitrile (2.0 eq.), Cu_2_O (10 mol%), Bathocuproine (20 mol%), *t*-BuOK (2.0 eq.), KF (2.0 eq.) in the solvent of DMF : H_2_O = 2 : 1, at 120 °C for 3.5 hours]. The results are listed in Table 2.

**Table 2:** Substrate scope exploration^a^^,^^b^

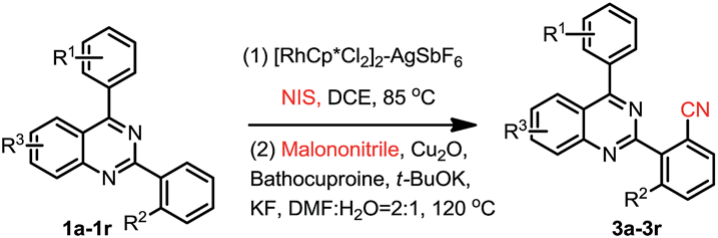
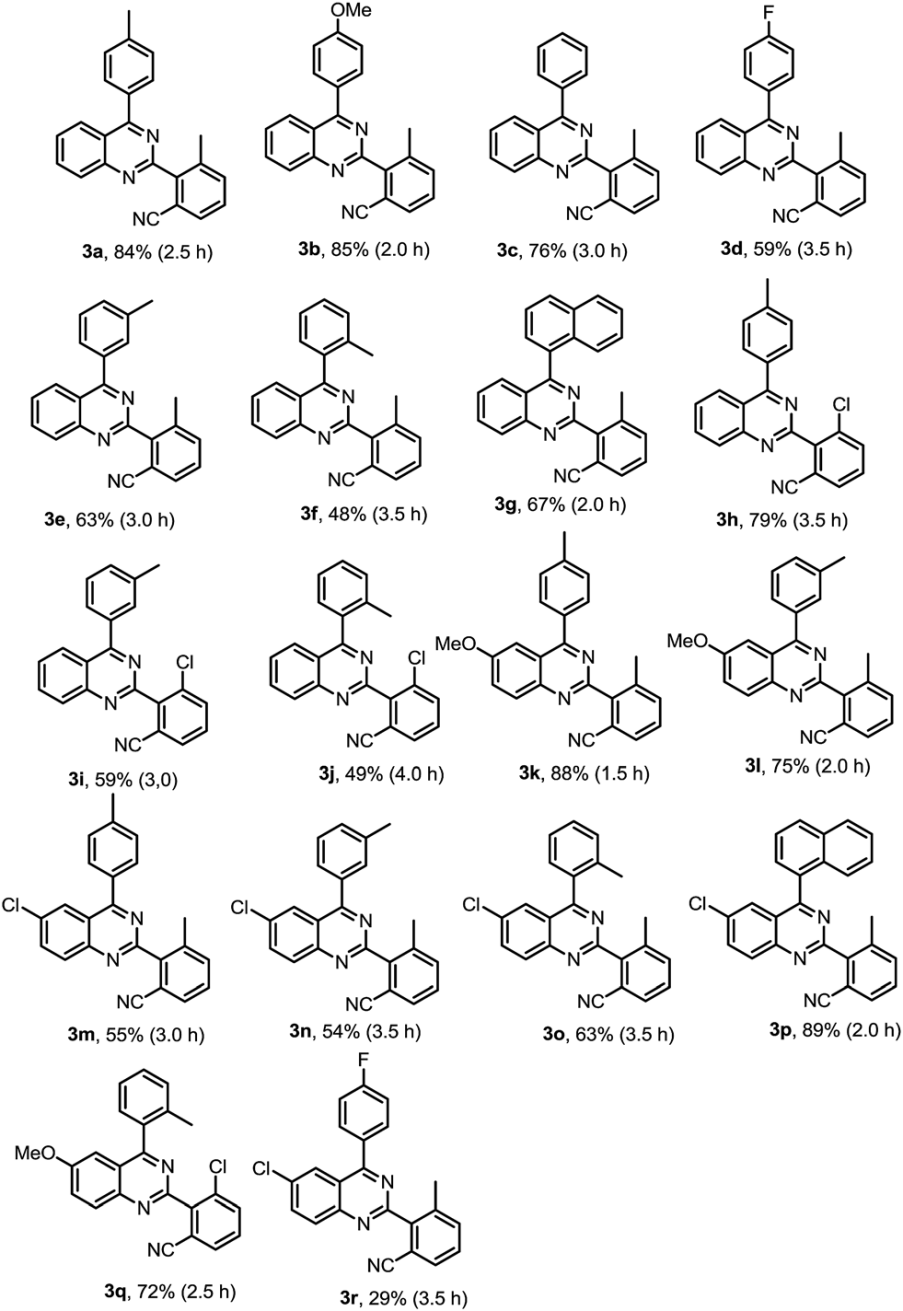

a Reaction conditions: 1a (0.2 mmol), NIS (1.5 eq.), [RhCp*Cl_2_]_2_ (1.0 mol%), AgSbF_6_ (8.0 mol%), DEC 2.0 mL, malononitrile (2.0 eq.), Cu_2_O (10 mol%), Bathocuproine (20 mol%), *t*-BuOK (2.0 eq.), KF (2.0 eq.), DMF : H_2_O = 2 : 1 (3.0 mL).

b Isolated yield.

As shown in Table 2, a variety of 2-(*o*-canyophenyl)-4-arylquinazolines were generated under the standard experimental conditions. In general, the electron-donating R^1^, R^2^, R^3^ group substituent fascinated the reactions, and gave higher yields than that of electron-withdrawing ones. For examples, when R^2^ is a methyl, and R^1^ is *p*-methyl or *p*-methoxyl, the corresponding products 3a and 3b were obtained in excellent of yields of 84% and 85%. The reaction of electron-withdrawing fluorinated substrate 1d gave good yield of 59%. The substituent group at the *meta* or *ortho* position of 4-phenyl, due to the steric hindrance, finished the corresponding products 3e, 3f and 3g in a yield of 63%, 48% and 67%, respectively. When R^2^ is chloro, the reactions gave the corresponding products 3h–3j in good yields 79–49%.

Subsequently, the effects of substituent on the phenyl moiety of quinazoline mother ring were then examined. Pleasingly, methoxy and chloro functionalities were all tolerated, providing the desired products 3k–3q in good to excellent yields. 3r was obtained in a low yield due to double electron-withdrawing substituent effect.

As we seen, when R^2^ is H, the reaction may produce mono-cyanation and bis-cyanation selectivity. In our previous work, exclusively generate mono-iodination on the 2-aryl group was obtained catalyzed by PbCl_2_–PPh_3_, and then cyanation of the corresponding 2-(2-iodoaryl)quinazolines using conventional procedure^1*e*^ could selective giving 2-(2-mono-nitrile)quinazolines. Thus, here we focus our attention on selective to produce bis-cyanation quinazolines in one-pot reaction. The results are listed in Table 3.

**Table 3:** Substrate scope exploration^a^^,^^b^

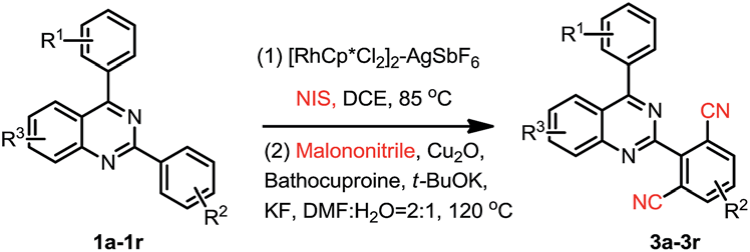
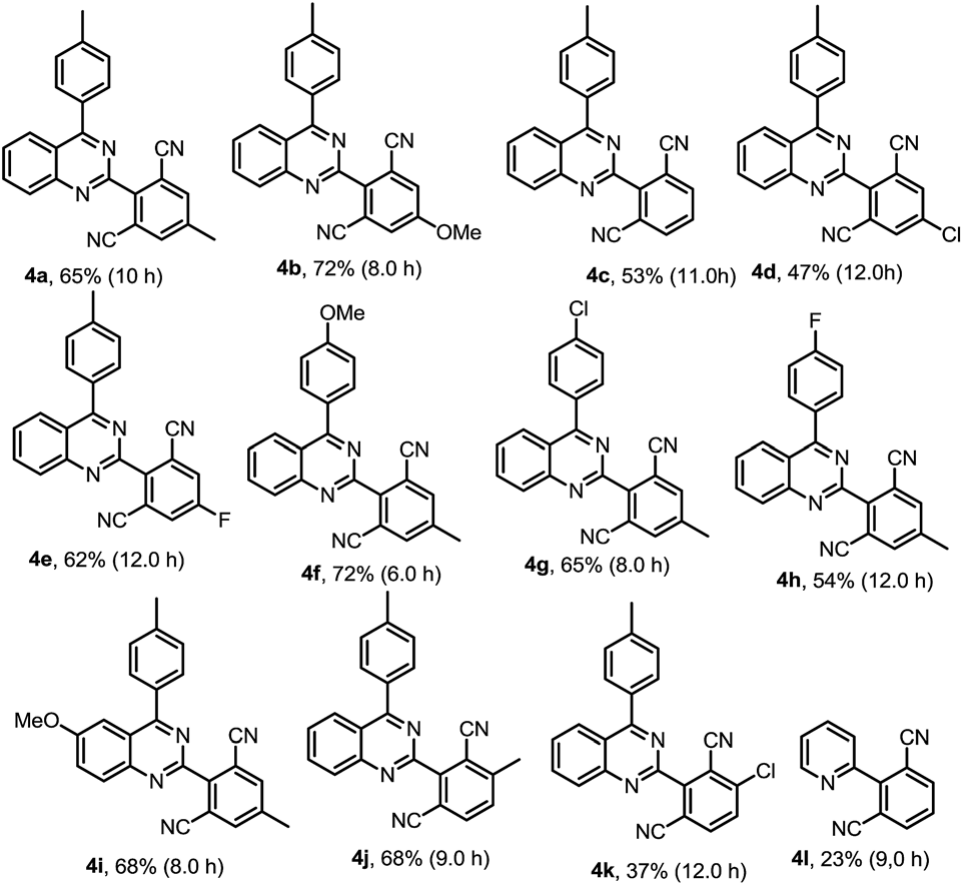

a Reaction conditions: (0.2 mmol), NIS (3.0 eq.). [RhCp*Cl_2_]_2_ (2.0 mol%), AgSbF_6_ (16 mol%), DEC (2.0 mL); malononitrile (4.0 eq.), Cu_2_O (20 mol%), Bathocuproine (40 mol%), *t*-BuOK (4.0 eq.), KF (4.0 eq.), DMF : H_2_O = 2 : 1 (3.0 mL).

b Isolated yield.

When 2,4-di-(*p*-methyl)phenylquinazoline 1aa was selected as the model substrate, and the corresponding regents were adding double of the standard protocol above, the corresponding bis-cyanation product 4a was obtained in a yield of 65% (Table 3). The electron-donating R^1^, R^2^, R^3^ group substituent also fascinating the reactions, and gave higher yields (4a, 4b, 4f and 4i) than that of electron-withdrawing ones (4d, 4e, 4g and 4h). Interestingly, dicyano-compounds were obtained in reasonable yields (4j and 4k) when *m*-substituted of 2,4-diarylquinazolines were used.

In order to further explore the substrate scope, 2-phenylpyridine was then investigated under the standard conditions, however, the corresponding product 4l was obtained in 23% yield. This result again disclosed that the directing properties of diazine in quinazoline are distinctive from that of pyridine.

**Scheme 2:**
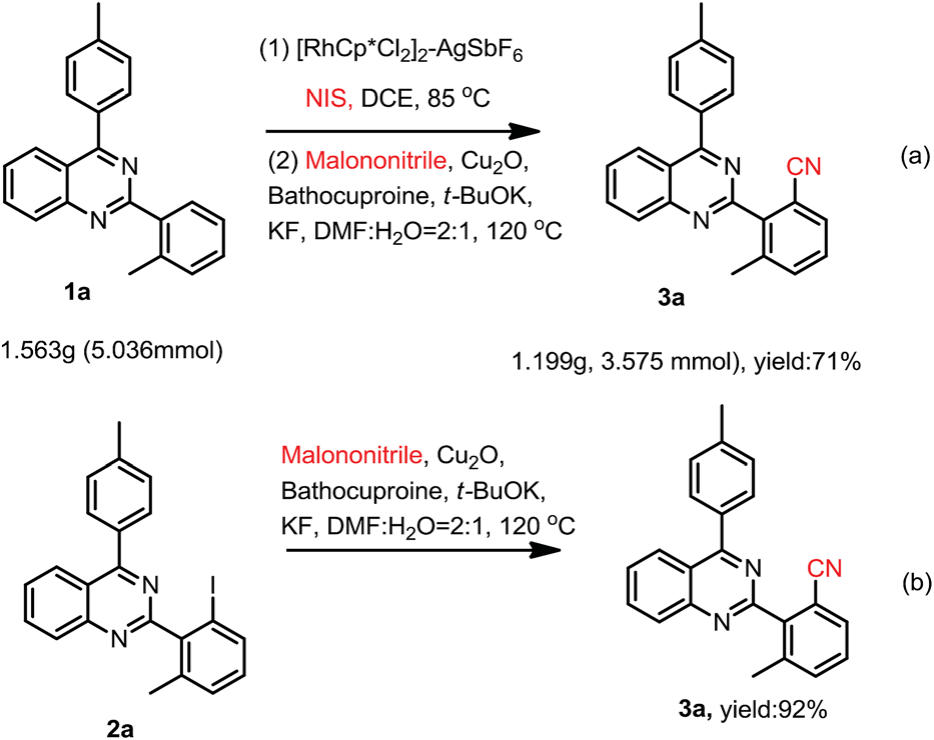
A gram-scale reaction and controlled experiment.

To demonstrate the potential synthetic utility of this transformation, a gram-scale reaction of 2-(*o*-methyl)phenyl-4-(*p*-methyl)phenylquinazoline (1a) was carried out. As shown in Scheme 2a, the cyanation product (3a) was isolated in an 71% yield.

To further understand the reaction mechanism, a controlled experiment was conducted. When 2-(2-iodo-6-methylphenyl)-4-(*p*-methyl)phenylquinazoline (2a) was used as the reactant under the standard reaction conditions in Scheme 2b, the cyanation product (3a) was isolated in an 92% yield.

**Scheme 3:**
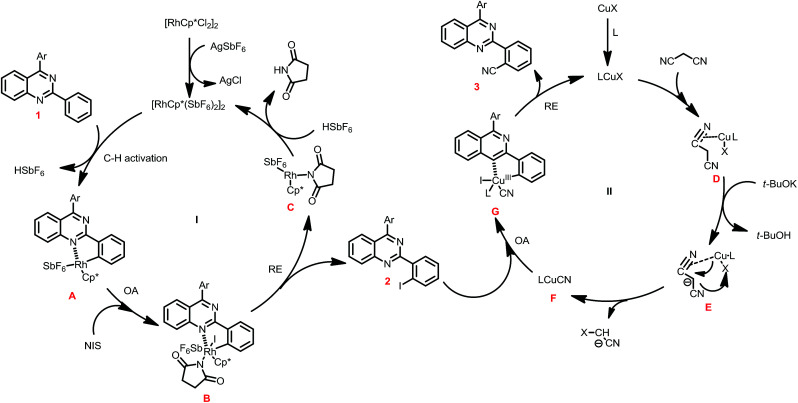
A plausible reaction mechanism.

In view of the above results, a plausible mechanism was disclosed in Scheme 3. The one-pot iodination/cyanation reaction include two catalytic cycles. In the first catalytic cycle I, the reaction of [Cp*RhCl_2_]_2_ and AgSbF_6_ forms [Cp*Rh(SbF_6_)_2_]_2_, which reacted with 1 through C–H activation to give five-membered rhodacycle A, and then was oxidation adduction with NIS to provide the Rh(iv) intermediate B, followed reductive eliminated to give iodide intermediate product 2 along with the Rh(iii) C, which was acidized to regenerate catalyst [Cp*Rh(SbF_6_)_2_]_2_ and finish catalytic cycle I.

In the second catalytic cycle II, the ligand was incorporated with Cu^I^X to form the catalyst LCuX, and then incorporate with malononitrile to provide the complex D, which was reacted with *t*-BuOK to give intermediate E, and then undergoes transmetalation to generate the active species LCu^I^CN. The LCu^I^CN subjected to oxidative adduction with 2 to form five-membered cyclic Cu(iii) G, and followed reductive eliminated to give product 3, and release the LCu^I^X catalyst and finish the catalytic cycle.

In summary, we here have developed methods for one-pot process for selective the synthesis of 2-(2-cyano)aryl-4-aryiquinazolines or 2-(2,6-dicyano)aryl-4-arylquinazolines. The one-pot reaction include two steps, the Rh-catalyzed selective C–H activation iodization of 2,4-diarylquinazoline with NIS, and then Cu-catalyzed cyanation of the corresponding iodide intermediate with malononitrile to give 2-(*o*-cyanoaryl)-4-arylquinazolines or 2-(2,6-dicyano)aryl-4-arylquinazolines in good to excellent yields.

## Experimental section

Unless otherwise noted, commercial reagents were purchased from Aldrich, Alfa, or other commercial suppliers. All solvents were dried and distilled according to standard procedures before use. Reactions were conducted in standard techniques on vacuum line. Analytical thin-layer chromatography (TLC) was performed using glass plates pre-coated with 0.25 mm 230–400 mesh silica gel impregnated with a fluorescent indicator (254 nm). Flash column chromatography was performed using silica gel (60 Å pore size, 32–63 μm, standard grade). Organic solutions were concentrated on rotary evaporators at ∼20 torr (house vacuum) at 25–35 °C. Nuclear magnetic resonance (NMR) spectra are recorded in parts per million (ppm) from internal standard tetramethylsilane (TMS) on the *δ* scale.

### General procedure for preparation of 2-(*o*-cyanoaryl)-4-arylquinazoline 3 (3a as an example)

A mixture of 2,4-phenylquinazolines 1a (0.2 mmol), NIS (0.3 mmol, 1.5 eq.), [RhCp*Cl_2_]_2_ (1.0 mol%), AgSbF_6_ (8.0 mol%) in DCE (2.0 mL) was stirred at 85 °C, until 1a was completed consumed (detected by TLC). The solvent was removed under reduced pressure. A mixture of malononitrile (2.0 eq.), Cu_2_O (10 mol%), Bathocuproin (20 mol%), *t*-BuOK (2.0 eq.), KF (2.0 eq.) and DMF and water (3.0 mL 2 : 1) was added and stirred at 120 °C for 3 h. After completion of the reaction as indicated by TLC, the mixture was cooled to room temperature. The solvent was evaporated, residue was diluted with EtOAc (10 mL), washed with H_2_O (10 mL), dried by anhydrous Na_2_SO_4_. Evaporation of the solvent followed purification by column chromatograph over silica gel provided the corresponding product 3a.

### General procedure for preparation of 2-(*o*-dicyanoaryl)-4-arylquinazoline 4 (4a as an example)

A mixture of 2,4-phenylquinazolines 1aa (0.2 mmol), NIS (0.6 mmol. 3.0 eq.), [RhCp*Cl_2_]_2_ (2.0 mol%), AgSbF_6_ (16 mol%) and in DCE (2.0 mL) was stirred at 85 °C for 0.5 h, until 1a was completed consumed. The solvent was removed under reduced pressure. A mixture of malononitrile (4.0 eq.), Cu_2_O (20 mol%), Bathocuproin (40 mol%), *t*-BuOK (4.0 eq.), KF (4.0 eq.) in DMF and water (3.0 mL 2 : 1) was stirred at 120 °C. After completion of the reaction as indicated by TLC, the mixture was cooled to room temperature. The solvent was evaporated, residue was diluted with EtOAc (10 mL), washed with H_2_O (10 mL), dried by anhydrous Na_2_SO_4_. Evaporation of the solvent followed purification by column chromatograph over silica gel provided the corresponding product 4a.

#### 2-(2-Cyano-6-methylphenyl)-4-(*p*-tolyl)quinazoline (3a)

Compound was obtained as a white solid: yield 84%; mp 109–112 °C; H NMR (400 MHz, CDCl_3_) *δ* 8.26 (d, *J* = 8.4 Hz, 1H), 8.18 (d, *J* = 8.4 Hz, 1H), 7.97 (t, *J* = 7.6 Hz, 1H), 7.82 (d, *J* = 8.0 Hz, 2H), 7.73–7.63 (m, 2H), 7.56 (d, *J* = 8.0 Hz, 1H), 7.44 (t, *J* = 7.6 Hz, 1H), 7.40 (d, *J* = 8.0 Hz, 2H), 2.48 (s, 6H). ^13^C NMR (100 MHz, CDCl_3_) *δ* 168.9, 160.2, 151.4, 142.5, 140.5, 138.3, 135.1, 134.1, 131.2, 130.3, 129.5, 129.1, 128.9, 128.3, 127.4, 121.6, 118.6, 113.1, 21.5, 20.4. HRMS (ESI): *m*/*z* [M + H]^+^ calcd for C_23_H_18_N_3_: 336.1495; found: 336.1501.

#### 2-(2-Cyano-6-methylphenyl)-4-(4-methoxyphenyl)quinazoline (3b)

Compound was obtained as a white solid: yield 85%; mp 146–148 °C; ^1^H NMR (400 MHz, CDCl_3_) *δ* 8.20 (d, *J* = 8.8 Hz, 1H), 8.09 (d, *J* = 8.4 Hz, 1H), 7.89 (d, *J* = 7.2 Hz, 1H), 7.83 (d, *J* = 8.8 Hz, 2H), 7.63–7.57 (m, 2H), 7.47 (d, *J* = 7.6 Hz, 1H), 7.35 (t, *J* = 7.6 Hz, 1H), 7.03 (d, *J* = 8.8 Hz, 2H), 3.83 (s, 3H), 2.40 (s, 3H). ^13^C NMR (100 MHz, CDCl_3_) *δ* 168.3, 161.4, 160.1, 151.4, 142.5, 138.3, 135.1, 134.1, 132.0, 131.2, 129.5, 129.1, 128.9, 128.2, 127.3, 121.6, 118.7, 114.2, 113.1, 55.5, 20.4. HRMS (ESI): *m*/*z* [M + H]^+^ calcd for C_23_H_18_N_3_O: 352.1444; found: 352.1450.

#### 2-(2-Cyano-6-methyl)phenyl-4-phenylquinazoline (3c)

Compound was obtained as a colorless oil: yield 76%; ^1^H NMR (400 MHz, CDCl_3_) *δ* 8.24 (dd, *J* = 8.4, 1.6 Hz, 1H), 8.20 (d, *J* = 8.4 Hz, 1H), 7.98 (ddd, *J* = 8.0, 6.8, 1.6 Hz, 1H), 7.93–7.91 (m, 2H), 7.71–7.61 (m, 2H), 7.6–7.55 (m, 4H), 7.44 (t, *J* = 8.0 Hz, 1H), 2.48 (s, 3H). ^13^C NMR (100 MHz, CDCl_3_) *δ* 168.8, 160.2, 151.4, 138.3, 137.0, 135.1, 134.2, 131.2, 130.2, 130.1, 129.2, 128.9, 128.7, 128.4, 127.3, 121.6, 118.6, 113.2, 20.4. HRMS (ESI): *m*/*z* [M + H]^+^ calcd. for C_22_H_16_N_3_: 322.1339; found: 322.1342.

#### 2-(2-Cyano-6-methylphenyl)-4-(4-fluorophenyl)quinazoline (3d)

Compound was obtained as a white solid: yield 59%; mp 122–124 °C; ^1^H NMR (400 MHz, CDCl_3_) *δ* 8.20 (d, *J* = 8.8 Hz, 2H), 8.01–7.93 (m, 3H), 7.73–7.68 (m, 2H), 7.57 (d, *J* = 8.0 Hz, 1H), 7.45 (t, *J* = 8.0 Hz, 1H), 7.32–7.27 (m, 2H), 2.49 (s, 3H). ^13^C NMR (100 MHz, CDCl_3_) *δ* 167.6, 164.1 (d, ^1^*J* = 249.0 Hz), 160.1, 151.5, 142.2, 138.3, 135.2, 134.3, 133.1 (d, ^4^*J* = 3.0 Hz), 132.4 (d, ^3^*J* = 9.0 Hz), 131.2, 129.3, 129.0, 128.5, 126.9, 121.5, 118.6, 115.9 (d, ^2^*J* = 22.0 Hz), 113.2, 20.3. HRMS (ESI): *m*/*z* [M + H]^+^ calcd for C_22_H_15_FN_3_: 340.1245, found: 340.1250.

#### 2-(2-Cyano-6-methylphenyl)-4-(3-methylphenyl)-quinazoline (3e)

Compound was obtained as a yellow oil: yield 63%; ^1^H NMR (400 MHz, CDCl_3_) *δ* 8.23 (d, *J* = 8.4 Hz, 1H), 8.19 (d, *J* = 8.4 Hz, 1H), 7.97 (t, *J* = 7.6 Hz, 1H), 7.73 (s, 1H), 7.70–7.66 (m, 3H), 7.55 (d, *J* = 7.6 Hz, 1H), 7.49–7.37 (m, 3H), 2.48 (s, 3H), 2.47 (s, 3H); ^13^C NMR (100 MHz, CDCl_3_) *δ* 169.1, 160.2, 151.4, 142.5, 138.6, 138.3, 136.9, 135.1, 134.2, 131.1, 131.0, 130.7, 129.1, 128.9, 128.5, 128.3, 127.4, 121.7, 118.6, 113.1, 21.6, 20.4. HRMS (ESI): *m*/*z* [M + H]^+^ calcd for C_23_H_18_N_3_: 336.1495, found: 336.1501.

#### 2-(2-Cyano-6-methylphenyl)-4-(2-methylphenyl)quinazoline (3f)

Compound was obtained as a yellow oil: yield 48%; ^1^H NMR (400 MHz, CDCl_3_) *δ* 8.20 (d, *J* = 8.4 Hz, 1H), 7.98 (ddd, *J* = 8.4, 6.8, 1.6 Hz, 1H), 7.76 (dd, *J* = 8.4, 1.6 Hz, 1H), 7.67–7.61 (m, 2H), 7.54 (d, *J* = 8.0 Hz, 1H), 7.46–7.41 (m, 3H), 7.39–7.34 (m, 2H), 2.40 (s, 3H), 2.21 (s, 3H). ^13^C NMR (100 MHz, CDCl_3_) *δ* 170.6, 160.5, 150.8, 142.6, 138.0, 136.3, 136.2, 134.9, 134.5, 130.8, 130.6, 129.5, 129.3, 129.0, 128.9, 128.4, 127.3, 125.8, 122.6, 118.2, 113.0, 20.1, 20.0. HRMS (ESI): *m*/*z* [M + H]^+^ calcd for C_23_H_18_N_3_: 336.1495, found: 336.1503.

#### 2-(2-Cyano-6-methylphenyl)-4-(naphthalen-1-yl)quinazoline (3g)

Compound was obtained as a white solid: yield 67%; mp 148–150 °C; ^1^H NMR (400 MHz, CDCl_3_) *δ* 8.25 (d, *J* = 8.4 Hz, 1H), 8.05 (d, *J* = 8.0 Hz, 1H), 8.01–7.96 (m, 2H), 7.7–7.69 (m, 2H), 7.67–7.63 (m, 3H), 7.58–7.51 (m, 3H), 7.45–7.40 (m, 2H), 2.45 (s, 3H). ^13^C NMR (100 MHz, CDCl_3_) *δ* 169.8, 160.6, 151.0, 142.5, 138.1, 135.0, 134.7, 134.2, 133.7, 131.5, 130.9, 130.1, 129.1, 129.0, 128.5, 128.4, 128.1, 127.6, 127.0, 126.4, 125.8, 125.1, 123.3, 118.4, 113.0, 20.3. HRMS (ESI): *m*/*z* [M + H]^+^ calcd for C_26_H_18_N_3_: 372.1495, found: 372.1501.

#### 2-(2-Cyano-6-chlorophenyl)-4-(*p*-tolyl)quinazoline (3h)

Compound was obtained as a white solid: yield 79%; mp 121–124 °C; ^1^H NMR (400 MHz, CDCl_3_) *δ* 8.26 (d, *J* = 8.4 Hz, 1H), 8.20 (d, *J* = 8.4 Hz, 1H), 7.98 (td, *J* = 8.46.8, 1.2 Hz, 1H), 7.81 (d, *J* = 8.0 Hz, 2H), 7.75 (ddd, *J* = 8.0, 7.2, 0.8 Hz 2H), 7.69 (ddd, *J* = 8.4, 7.2, 0.8 Hz, 1H), 7.48 (t, *J* = 8.0 Hz, 1H), 7.40 (d, *J* = 8.0 Hz, 2H), 2.47 (s, 3H). ^13^C NMR (100 MHz, CDCl_3_) *δ* 169.1, 158.3, 151.4, 141.9, 140.6, 134.6, 134.5, 134.2, 134.0, 131.7, 130.3, 129.9, 129.5, 129.2, 128.6, 127.4, 122.0, 117.0, 114.9, 21.5. HRMS (ESI): *m*/*z* [M + H]^+^ calcd for C_23_H_21_N_2_O: 406.1106, found: 406.1102.

#### 2-(2-Cyano-6-chlorophenyl)-4-(3-methylphenyl)quinazoline (3i)

Compound was obtained as a yellow oil: yield 59%; ^1^H NMR (400 MHz, CDCl_3_) *δ* 8.25 (dd, *J* = 8.4, 0.4 Hz, 1H), 8.21 (d, *J* = 8.4 Hz, 1H), 7.99 (ddd, *J* = 8.4, 7.6, 1.6 Hz, 1H), 7.78–7.66 (m, 5H), 7.50 (d, *J* = 7.6 Hz, 1H), 7.47 (d, *J* = 7.2 Hz, 1H), 7.39 (d, *J* = 7.6 Hz, 1H), 2.48 (s, 3H). ^13^C NMR (100 MHz, CDCl_3_) *δ* 169.3, 158.3, 151.3, 141.9, 138.7, 136.7, 134.6, 134.5, 134.3, 131.7, 131.1, 130.7, 130.0, 129.2, 128.7, 128.6, 127.5, 127.4, 122.0, 117.0, 114.9, 21.5. HRMS (ESI): *m*/*z* [M + H]^+^ calcd for C_22_H_15_ClN_3_: 356.0949, found: 356.0955.

#### 2-(2-Cyano-6-chlorophenyl)-4-(2-methylphenyl)quinazoline (3j)

Compound was obtained as a yellow solid: yield 49%; mp 101–103 °C; ^1^H NMR (400 MHz, CDCl_3_) *δ* 8.23 (d, *J* = 8.4 Hz, 1H), 8.00 (ddd, *J* = 8.4, 6.8, 1.6 Hz, 1H), 7.78 (d, *J* = 8.4 Hz, 1H), 7.74 (dt, *J* = 8.0, 1.6 Hz, 2H), 7.66 (ddd, *J* = 8.4, 6.8, 1.2 Hz, 1H), 7.49 (t, *J* = 8.0 Hz, 1H), 7.46–7.34 (m, 4H), 2.23 (s, 3H). ^13^C NMR (100 MHz, CDCl_3_) *δ* 170.9, 158.5, 150.9, 142.0, 136.4, 136.0, 134.7, 134.5, 134.3, 131.5, 130.7, 130.1, 129.6, 129.4, 129.1, 128.8, 127.3, 125.8, 123.0, 116.8, 114.7, 20.0. HRMS (ESI): *m*/*z* [M + H]^+^ calcd for C_22_H_15_ClN_3_: 356.0949, found: 356.0951.

#### 2-(2-Cyano-6-methylphenyl)-4-(*p*-tolyl)-6-methoxyl quinazoline (3k)

Compound was obtained as a colorless oil: yield 88%; ^1^H NMR (400 MHz, CDCl_3_) *δ* 8.08 (d, *J* = 8.8 Hz, 1H), 7.82 (d, *J* = 8.0 Hz, 2H), 7.66 (d, *J* = 7.2 Hz, 1H), 7.61 (dd, *J* = 9.2, 2.8 Hz, 1H), 7.54 (d, *J* = 7.6 Hz, 1H), 7.50 (d, *J* = 2.8 Hz, 1H), 7.43–7.38 (m, 3H), 3.89 (s, 3H), 2.48 (s, 3H), 2.46 (s, 3H). ^13^C NMR (100 MHz, CDCl_3_) *δ* 167.0, 159.0, 158.3, 147.6, 142.6, 140.2, 138.3, 135.0, 134.5, 131.1, 130.6, 129.9, 129.5, 128.7, 126.8, 122.6, 113.2, 104.5, 55.8, 21.5, 20.4. HRMS (ESI): *m*/*z* [M + H]^+^ calcd for C_24_H_20_N_3_O: 366.1601, found: 366.1606.

#### 2-(2-Cyano-6-methylphenyl)-4-(3-methylphenyl)-6-methoxylquinazoline (3l)

Compound was obtained as a colorless oil: yield 75%; ^1^H NMR (400 MHz, CDCl_3_) *δ* 8.09 (d, *J* = 9.2 Hz, 1H), 7.74 (s, 1H), 7.6.9 (d, *J* = 8.0 Hz, 1H), 7.66 (d, *J* = 8.4 Hz, 1H), 7.62 (dd, *J* = 9.2, 2.8 Hz, 1H), 7.54 (d, *J* = 7.6 Hz, 1H), 7.49–7.37 (m, 4H), 3.88 (s, 3H), 2.48 (s, 3H), 2.45 (s, 3H). ^13^C NMR (100 MHz, CDCl_3_) *δ* 167.2, 159.0, 158.3, 147.6, 142.6, 138.7, 138.3, 137.3, 135.0, 131.1, 130.8, 130.6, 130.5, 128.7, 128.5, 126.93, 126.9, 122.6, 118.6, 113.2, 104.5, 55.8, 21.6, 20.3. HRMS (ESI): *m*/*z* [M + H]^+^ calcd for C_24_H_20_N_3_O: 366.1601, found: 366.1606.

#### 2-(2-Cyano-6-methylphenyl)-4-(*p*-tolyl)-6-chloro-quinazoline (3m)

Compound was obtained as a yellow solid: yield 55%; mp 106–108 °C; ^1^H NMR (400 MHz, CDCl_3_) *δ* 8.22 (d, *J* = 2.0 Hz, 1H), 8.13 (d, *J* = 8.8 Hz, 1H), 7.90 (dd, *J* = 8.8, 2.4 Hz, 1H), 7.80 (d, *J* = 8.0 Hz, 2H), 7.68 (d, *J* = 7.6 Hz, 1H), 7.56 (d, *J* = 8.0 Hz, 1H), 7.46–7.41 (m, 3H), 2.48 (s, 3H), 2.47 (s, 3H). ^13^C NMR (100 MHz, CDCl_3_) *δ* 168.0, 160.4, 149.9, 142.0, 140.9, 138.3, 135.1, 135.1, 134.1, 133.6, 131.2, 130.8, 130.2, 129.7, 129.0, 126.1, 122.2, 118.5, 113.2, 21.5, 20.4. HRMS (ESI): *m*/*z* [M + H]^+^ calcd for C_23_H_17_ClN_3_: 370.1106, found: 370.1111.

#### 2-(2-Cyano-6-methylphenyl)-4-(3-methylphenyl)-6-chloro-quinazoline (3n)

Compound was obtained as a yellow oil: yield 54%; ^1^H NMR (400 MHz, CDCl_3_) *δ* 8.20 (d, *J* = 2.0 Hz, 1H), 8.13 (d, *J* = 9.2 Hz, 1H), 7.90 (dd, *J* = 9.6, 2.0 Hz, 1H), 7.70–7.64 (m, 3H), 7.55 (d, *J* = 7.6 Hz, 1H), 7.51–7.40 (m, 3H), 2.49 (s, 3H), 2.46 (s, 3H). ^13^C NMR (100 MHz, CDCl_3_) *δ* 168.3, 160.4, 149.9, 142.1, 138.9, 138.3, 136.4, 135.2, 135.1, 134.1, 131.3, 131.2, 130.8, 130.6, 129.1, 128.7, 127.3, 126.1, 122.2, 118.5, 113.2, 21.6, 20.4. HRMS (ESI): *m*/*z* [M + H]^+^ calcd for C_23_H_17_ClN_3_: 370.1106, found: 370.1111.

#### 2-(2-Cyano-6-methylphenyl)-4-(2-methylphenyl)-6-chloro-quinazoline (3o)

Compound was obtained as a yellow oil: yield 63%; ^1^H NMR (400 MHz, CDCl_3_) *δ* 8.15 (d, *J* = 9.2 Hz, 1H), 7.90 (dd, *J* = 9.2, 2.4 Hz, 1H), 7.72 (d, *J* = 2.0 Hz, 1H), 7.65 (d, *J* = 7.6 Hz, 1H), 7.54 (d, *J* = 7.6 Hz, 1H), 7.48–7.38 (m, 5H), 2.39 (s, 3H), 2.22 (s, 3H). ^13^C NMR (100 MHz, CDCl_3_) *δ* 169.9, 160.7, 149.4, 142.2, 138.0, 136.2, 135.6, 135.5, 135.0, 134.3, 130.9, 130.9, 130.8, 129.8, 129.3, 129.1, 126.0, 125.9, 123.2, 118.2, 113.0, 20.2, 20.1. HRMS (ESI): *m*/*z* [M + H]^+^ calcd for C_23_H_17_ClN_3_: 370.1106, found: 370.1113.

#### 2-(2-Cyano-6-methylphenyl)-4-(naphthalen-1-yl)-6-chloro-quinazoline (3p)

Compound was obtained as a yellow oil: yield 89%; ^1^H NMR (400 MHz, CDCl_3_) *δ* 8.20 (d, *J* = 8.8 Hz, 1H), 8.07 (dd, *J* = 7.2, 1.6 Hz, 1H), 7.98 (d, *J* = 8.0 Hz, 1H), 7.92 (dd, *J* = 8.8, 2.0 Hz, 1H), 7.72 (d, *J* = 2.4 Hz, 1H), 7.72–7.63 (m, 3H), 7.62 (s, 1H), 7.57–7.53 (m, 2H), 7.48–7.41 (m,, 2H), 2.44 (s, 3H). ^13^C NMR (100 MHz, CDCl_3_) *δ* 169.04, 160.8, 149.6, 142.1, 138.1, 135.7, 135.0, 134.30, 133.8, 133.5, 131.4, 131.0, 130.80, 130.4, 129.1, 128.5, 128.1, 127.2, 126.6, 126.2, 125.5, 125.1, 123.9, 118.3, 113.0, 20.3. HRMS (ESI): *m*/*z* [M + H]^+^ calcd for C_26_H_17_ClN_3_: 406.1106, found: 406.1102.

#### 2-(2-Cyano-6-chlorophenyl)-4-(2-methylphenyl)-6-methoxyl quinazoline (3q)

Compound was obtained as a yellow oil: yield 72%; ^1^H NMR (400 MHz, CDCl_3_) *δ* 8.12 (d, *J* = 9.2 Hz, 1H), 7.72 (ddd, *J* = 9.2, 8.0, 1.2 Hz, 2H), 7.63 (dd, *J* = 9.6, 2.8 Hz, 1H), 7.49–7.34 (m, 5H), 6.96 (d, *J* = 2.8 Hz, 1H), 3.80 (s, 3H), 2.24 (s, 3H). ^13^C NMR (100 MHz, CDCl_3_) *δ* 168.8, 159.3, 156.4, 147.1, 142.1, 136.3, 136.3, 134.6, 134.3, 131.4, 130.8, 130.6, 129.9, 129.5, 129.2, 127.5, 125.9, 124.0, 116.9, 114.8, 104.2, 55.8, 20.0. HRMS (ESI): *m*/*z* [M + H]^+^ calcd for C_23_H_17_ClN_3_O: 386.1055, found: 386.1057.

#### (2-Cyano-6-methylphenyl)-4-(4-fluorophenyl)-6-chloro-2-quinazoline (3r)

Compound was obtained as a yellow solid: yield 29%; mp: 126–128 °C; ^1^H NMR (400 MHz, CDCl_3_) *δ* 8.17 (d, *J* = 2.0 Hz, 1H), 8.15 (d, *J* = 8.8 Hz, 1H), 7.95–7.91 (m, 3H), 7.69 (d, *J* = 7.2 Hz, 1H), 7.57 (d, *J* = 8.0 Hz, 1H), 7.46 (t, *J* = 8.0 Hz, 1H), 7.34–7.29 (m, 2H), 2.48 (s, 3H). ^13^C NMR (100 MHz, CDCl_3_) *δ* 166.8, 164.4 (d, ^1^*J* = 238.0 Hz), 163.0, 150.0, 141.8, 138.3, 135.3(d, ^3^*J* = 11.0 Hz) 134.4, 132.5(^4^*J* = 3.0 Hz), 123.4, 132.3, 131.3, 131.0, 129.2, 125.7, 122.0, 118.6, 116.2 (d, ^2^*J* = 21.0 Hz), 113.2, 20.4. HRMS (ESI): *m*/*z* [M + H]^+^ calcd for C_22_H_14_ClFN_3_: 374.0855, found: 374.0860.

#### 2-(2,6-Dicyano-4-methylphenyl)-4-(*p*-tolyl)quinazoline (4a)

Compound was obtained as a white solid: yield 65%; mp 158–160 °C; ^1^H NMR (400 MHz, CDCl_3_) *δ* 8.30 (dd, *J* = 8.4, 1.2 Hz, 1H), 8.26 (dd, *J* = 8.4, 1.2 Hz, 1H), 8.00 (ddd, *J* = 8.4, 6.8, 1.2 Hz, 1H), 7.93 (d, *J* = 8.0 Hz, 2H), 7.70 (s, 2H), 7.71 (ddd, *J* = 8.4, 6.8, 1.2 Hz, 1H), 7.42 (d, *J* = 8.0 Hz, 2H), 2.53 (s, 3H), 2.49 (s, 3H). ^13^C NMR (100 MHz, CDCl_3_) *δ* 169.2, 156.3, 151.4, 142.4, 140.8, 140.6, 138.3, 134.4, 133.9, 130.5, 129.5, 129.3, 128.9, 127.4, 122.0, 117.3, 114.5, 21.5, 20.8. HRMS calcd. For C_24_H_17_N_4_: 361.1448, found: 361.1452.

#### 2-(2,6-Dicyano-4-methoxyphenyl)-4-(*p*-tolyl)quinazoline (4b)

Compound was obtained as a white solid: yield 72%; mp 194–196 °C; ^1^H NMR (400 MHz, CDCl_3_) *δ* 8.29 (dd, *J* = 8.8, 1.2 Hz, 1H), 8.25 (d, *J* = 9.2, 1.2 Hz, 1H), 7.98 (ddd, *J* = 8.4, 6.8, 1.2 Hz 1H), 7.92 (d, *J* = 8.0 Hz, 2H), 7.70 (ddd, *J* = 8.4, 7.6, 1.2 Hz 1H), 7.55 (s, 2H), 7.42 (d, *J* = 8.0 Hz, 2H), 3.96 (s, 3H), 2.49 (s, 3H). ^13^C NMR (100 MHz, CDCl_3_) *δ* 169.1, 159.7, 156.1, 151.5, 140.8, 137.3, 134.3, 134.0, 130.5, 129.5, 129.3, 128.7, 127.4, 123.4, 122.0, 117.1, 115.7, 56.4, 21.5. HRMS (ESI): *m*/*z* [M + H]^+^ calcd for C_24_H_17_N_4_O: 377.1397, found: 377.1402.

#### 2-(2,6-Dicyanophenyl)-4-(*p*-tolyl)quinazoline (4c)

Compound was obtained as a colorless oil: yield 53%; ^1^H NMR (400 MHz, CDCl_3_) *δ* 8.31 (d, *J* = 8.4 Hz, 1H), 8.27 (d, *J* = 8.4 Hz, 1H), 8.07 (d, *J* = 7.6 Hz, 2H), 8.01 (t, *J* = 8.0 Hz, 1H), 7.93 (d, *J* = 8.0 Hz, 2H), 7.76–7.67 (m, 2H), 7.43 (d, *J* = 8.0 Hz, 2H), 2.49 (s, 3H). ^13^C NMR (100 MHz, CDCl_3_) *δ* 169.3, 156.2, 151.4, 145.2, 140.9, 137.7, 134.6, 133.9, 130.5, 129.7, 129.6, 129.4, 129.1, 127.5, 122.1, 117.1, 114.7, 21.6. HRMS (ESI): *m*/*z* [M + H]^+^ calcd for C_23_H_15_N_4_: 347.1291, found: 347.1294.

#### 2-(2,6-Dicyano-4-chlorophenyl)-4-(*p*-tolyl)quinazoline (4d)

Compound was obtained as a yellow solid: yield 47%; mp: 172–175 °C; ^1^H NMR (400 MHz, CDCl_3_) *δ* 8.31 (d, *J* = 8.0 Hz, 1H), 8.26 (d, *J* = 8.4 Hz, 1H), 8.04–8.00 (m, 3H), 7.92 (d, *J* = 8.4 Hz, 2H), 7.74 (t, *J* = 7.6 Hz, 1H), 7.43 (d, *J* = 7.6 Hz, 2H), 2.49 (s, 3H). ^13^C NMR (100 MHz, CDCl_3_) *δ* 169.4, 155.4, 151.4, 143.3, 141.0, 137.5, 136.0, 134.6, 133.7, 130.5, 129.6, 129.4, 129.2, 127.5, 122.1, 116.1, 116.0, 21.6. HRMS (ESI): *m*/*z* [M + H]^+^ calcd for C_23_H_14_ClN_4_: 381.0902, found: 381.0907.

#### 2-(2,6-Dicyano-4-fluorophenyl)-4-(*p*-tolyl)quinazoline (4e)

Compound was obtained as a yellow solid: yield 62%; mp: 170–173 °C; ^1^H NMR (400 MHz, CDCl_3_) *δ* 8.30 (dd, *J* = 8.4, 0.4 Hz, 1H), 8.26 (d, *J* = 8.4 Hz, 1H), 8.01 (ddd, *J* = 8.4, 7.2, 1.2 Hz, 1H), 7.91 (d, *J* = 8.0 Hz, 2H), 7.78 (d, *J* = 7.6 Hz, 2H), 7.73 (ddd, *J* = 8.4, 7.2, 1.2 Hz, 1H), 7.42 (d, *J* = 8.0 Hz, 2H), 2.49 (s, 3H). ^13^C NMR (100 MHz, CDCl_3_) *δ* 169.4, 161.4 (d, ^1^*J* = 254.0 Hz), 155.4, 151.4, 141.7 (d, ^3^*J* = 4.0 Hz), 141.0, 134.6, 133.8, 130.5, 129.5, 129.3, 129.1, 127.5, 125.1 (d, ^2^*J* = 24.0 Hz), 122.1, 116.5 (d, ^3^*J* = 10.0 Hz), 116.0 (d, ^4^*J* = 2.0 Hz), 21.5. HRMS (ESI): *m*/*z* [M + H]^+^ calcd for C_23_H_14_FN_4_^+^ [M + H]^+^ 365.1197, found: 365.1121.

#### 2-(2,6-Dicyano-4-methylphenyl)-4-(4-methoxyphenyl)quinazoline (4f)

Compound was obtained as a white solid: yield 72%; mp 149–153 °C; ^1^H NMR (400 MHz, CDCl_3_) *δ* 8.31 (d, *J* = 8.4 Hz, 1H), 8.25 (d, *J* = 8.4 Hz, 1H), 8.03 (d, *J* = 8.8 Hz, 2H), 7.98 (ddd, *J* = 8.4, 6.8, 1.2 Hz, 1H), 7.86 (s, 2H), 7.71 (ddd, *J* = 8.4, 6.8, 1.2 Hz, 1H), 7.14 (d, *J* = 8.8 Hz, 2H), 3.92 (s, 3H), 2.53 (s, 3H). ^13^C NMR (100 MHz, CDCl_3_) *δ* 168.6, 161.7, 156.2, 151.5, 142.5, 140.6, 138.3, 134.3, 132.3, 129.4, 129.3, 128.8, 127.4, 122.0, 117.3, 114.5, 114.3, 55.5, 20.8; HRMS (ESI): *m*/*z* [M + H]^+^ calcd for C_24_H_17_N_4_O: 377.1397, found: 377.1404.

#### 2-(2,6-Dicyano-4-methylphenyl)-4-(4-chlorophenyl)quinazoline (4g)

Compound was obtained as a yellow solid: yield 65%; mp: 187–190 °C; ^1^H NMR (400 MHz, CDCl_3_) *δ* 8.28 (d, *J* = 8.4 Hz, 1H), 8.22 (d, *J* = 8.0 Hz, 1H), 8.01(ddd, *J* = 8.0, 6.8, 1.2 Hz, 1H), 7.97 (d, *J* = 8.4 Hz, 2H), 7.87 (s, 2H), 7.73 (ddd, *J* = 8.0, 6.8, 1.2 Hz, 1H), 7.60 (d, *J* = 8.4 Hz, 2H), 2.53 (s, 3H). ^13^C NMR (100 MHz, CDCl_3_) *δ* 167.8, 156.2, 151.5, 142.1, 140.8, 138.3, 136.9, 135.1, 134.6, 131.9, 129.6, 129.2, 129.1, 126.8, 121.8, 117.2, 114.5, 20.8. HRMS (ESI): *m*/*z* [M + H]^+^ calcd for C_23_H_14_ClN_4_: 381.0902, found: 381.0907.

#### 2-(2,6-Dicyano-4-methylphenyl)-4-(4-fluorophenyl)quinazoline (4h)

Compound was obtained as a yellow solid: yield 54%; mp 209–212 °C; ^1^H NMR (400 MHz, CDCl_3_) *δ* 8.28 (d, *J* = 8.4 Hz, 1H), 8.24 (d, *J* = 8.4 Hz, 1H), 8.06–8.0 (m, 3H), 7.87 (s, 2H), 7.74 (ddd, *J* = 8.0, 7.6, 1.2 Hz, 1H), 7.31 (t, *J* = 8.4 Hz, 2H), 2.53 (s, 3H). ^13^C NMR (100 MHz, CDCl_3_) *δ* 166.7(d, ^1^*J* = 243.0 Hz), 156.2, 151.5, 142.2, 140.8, 138.3, 134.6, 132.8 (d, ^4^*J* = 3.0 Hz), 132.7 (d, ^3^*J* = 9.0 Hz), 129.5, 129.1, 127.0, 121.9, 117.2, 116.1, 116.0 (d, ^2^*J* = 22.0 Hz), 114.5, 20.8; HRMS (ESI): *m*/*z* [M + H]^+^ calcd for C_23_H_14_FN_4_: 365.1197, found: 365.1121.

#### 2-(2,6-Dicyano-4-methylphenyl)-4-(*p*-tolyl)-6-methoxylquinazoline (4i)

Compound was obtained as a white solid: yield 68%; mp 156–159 °C; ^1^H NMR (400 MHz, CDCl_3_) *δ* 8.16 (d, *J* = 9.2 Hz, 1H), 7.93 (d, *J* = 8.0 Hz, 2H), 7.84 (s, 2H), 7.63 (dd, *J* = 9.2, 2.8 Hz, 1H), 7.54 (d, *J* = 2.8 Hz, 1H), 7.42 (d, *J* = 8.0 Hz, 2H), 3.90 (s, 3H), 2.51 (s, 3H), 2.48 (s, 3H). ^13^C NMR (100 MHz, CDCl_3_) *δ* 167.2, 159.5, 154.4, 147.6, 142.6, 140.5, 140.2, 138.2, 134.3, 130.8, 130.1, 129.5, 127.1, 123.1, 117.3, 114.4, 104.6, 55.8, 21.5, 20.7. HRMS (ESI): *m*/*z* [M + H]^+^ calcd for C_25_H_19_N_4_O: 391.1553, found: 391.1558.

#### 2-(2,6-Dicyano-3-methylphenyl)-4-(*p*-tolyl)quinazoline (4j)

Compound was obtained as a white solid: yield 68%; mp 128–130 °C; ^1^H NMR (400 MHz, CDCl_3_) *δ* 8.34 (s, 1H), 8.22 (dd, *J* = 8.4, 3.6 Hz, 2H), 7.94–7.89 (m, 3H), 7.75 (d, *J* = 8.0 Hz, 1H), 7.62 (t, *J* = 8.0 Hz, 1H), 7.41 (d, *J* = 8.0 Hz, 2H), 7.35 (d, *J* = 7.6 Hz, 1H), 2.51 (s, 3H), 2.48 (s, 3H). ^13^C NMR (100 MHz, CDCl_3_) *δ* 168.8, 158.5, 151.7, 143.4, 141.3, 140.6, 134.9, 134.3, 134.0, 131.3, 130.7, 130.6, 129.4, 129.2, 128.0, 127.3, 121.8, 119.7, 109.4, 21.9, 21.5. HRMS (ESI): *m*/*z* [M + H]^+^ calcd for C_24_H_17_N_4_: 361.1448, found: 361.1452.

#### 2-(2,6-Dicyano-3-chlorophenyl)-4-(*p*-tolyl)quinazoline (4k)

Compound was obtained as a yellow solid: yield 37%; mp: 157–160 °C; ^1^H NMR (400 MHz, CDCl_3_) *δ* 8.31 (d, *J* = 8.4 Hz, 1H), 8.26 (d, *J* = 8.4 Hz, 1H), 8.02 (ddd, *J* = 8.4, 7.2, 1.2 Hz, 1H), 7.96 (d, *J* = 8.4 Hz, 1H), 7.91 (d, *J* = 8.0 Hz, 2H), 7.77–7.73 (m, 2H), 7.43 (d, *J* = 7.6 Hz, 2H), 2.49 (s, 3H). ^13^C NMR (100 MHz, CDCl_3_) *δ* 169.4, 155.8, 151.4, 147.5, 142.9, 141.0, 137.5, 134.6, 133.7, 130.6, 130.5, 129.5, 129.4, 129.3, 127.5, 122.3, 116.3, 115.6, 113.9, 112.9, 21.5. HRMS (ESI): *m*/*z* [M + H]^+^ calcd for C_23_H_14_ClN_4_: 381.0902, found: 381.0907.

#### 2-(2,6-Dicyanophenyl)pyridine (4l)

Compound was obtained as a white solid: yield 23%; mp 140–142 °C; 1H NMR (400 MHz, CDCl_3_) *δ* 8.78 (d, *J* = 4.8 Hz, 1H), 7.86–7,78 (m, 3H), 7.72–7.68 (m, 1H), 7.51 (td, *J* = 7.6, 1.2 Hz, 1H), 7.37 (ddd, *J* = 7.6, 4.8, 1.2 Hz, 1H). ^13^C NMR (101 MHz, CDCl_3_) *δ* 155.3, 150.0, 143.5, 134.1, 132.8, 131.0, 130.0, 128.7, 123.3, 123.2. HRMS (ESI): *m*/*z* [M + H]^+^ calcd for C_12_H_9_N_2_: 181.0760, found: 181.0769.

## Conflicts of interest

There are no conflicts to declare.

## Supplementary Material

RA-009-C9RA02979F-s001
