# Supplementary material for: One-pot regioselective C–H activation iodination–cyanation of 2,4-diarylquinazolines using malononitrile as a cyano source
Source: RSC Adv. 2019 Jun 11;9(32):18256–64. doi: 10.1039/c9ra02979f (PMC9064619; doi:10.1039/c9ra02979f)

Electronic Supplementary Material (ESI) for RSC Advances.
This journal is © The Royal Society of Chemistry 2019

One-pot regioselective C-H activation iodination - cyanonation of 2,4-diarylquinazolines using malononitrile as a cyano source

Ziqiao Yan,[a] Banlai Ouyang[b], Xunchun Mao[a], Wei Gao[a], Zhihong Deng[a] and Yiyuan Peng[*a]

[a] *Key Laboratory of Functional Small Organic Molecules, Ministry of Education, Jiangxi Province's Key Laboratory of Green Chemistry, and Jiangxi Normal University, Nanchang 330022, PR China*

[b]*Department of Chemistry, Nanchang Normal University, Nanchang 330032, PR China*

1. General experimental methods......... 1

2. General procedure for the preparation of substrate **3** and **4**... .... (1-2)

3. ^{1}H and ^{13}C NMR spectra of compounds **3** and **4** **......** ....**.............**(3-32)

Experimental section.

General experimental methods: Unless otherwise noted, commercial reagents were purchased from Aldrich, Alfa Aesar, or other commercial suppliers, and were used as received. All solvents were dried and distilled according to standard procedures before use. Reactions were conducted in standard schlenk techniques on vacuum line. Analytical thin-layer chromatography (TLC) was performed using glass plates pre-coated with 0.25 mm 230-400 mesh silica gel impregnated with a fluorescent indicator (254 nm). Flash column chromatography was performed using silica gel (60-Å pore size, 32-63 μm, standard grade). Organic solutions were concentrated on rotary evaporators at ~20 Torr (house vacuum) at 25-35 °C. Nuclear magnetic resonance (NMR) spectra are recorded in parts per million (ppm) from internal standard tetramethylsilane (TMS) on the δ scale.

General procedure for the preparation of substrate 3

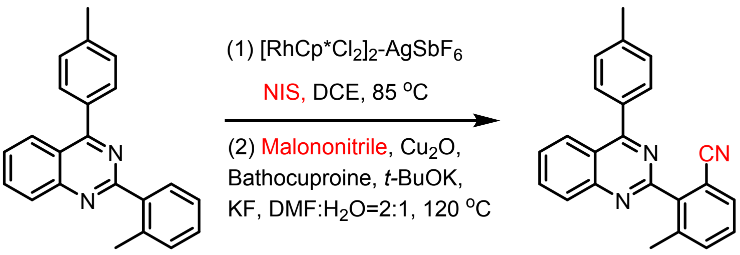

1a **3a**

A mixture of 2,4-phenylquinazolines **1a** (0.2 mmol, 1.0 equiv), NIS (0.3mmol. 1.5eq), $[RhCp^*Cl_2]_2$ (1mol%), $AgSbF_6$(8 mol%) and in DCE (2.0 mL) was stirred at 85°C under for 0.5 h, until **1a** was completed consumed. The solvent was removed under reduced pressure. A mixture of malononitrile (2.0 eq), Cu_2O (10 mol%), Bathocuproine (20 mol%), t-BuOK(2.0 eq), KF (2.0 eq) in DMF and water (3mL, 2:1) was stirred at 120°C. After completion of the reaction as indicated by TLC, the mixture was cooled to room temperature. The solvent was evaporated, residue was diluted with EtOAc (10 mL), washed with H_2O (10 mL), dried by anhydrous Na_2SO_4. Evaporation of the solvent followed purification by column chromatograph over silica gel provided the corresponding product **3a**.

General procedure for the preparation of substrate 4

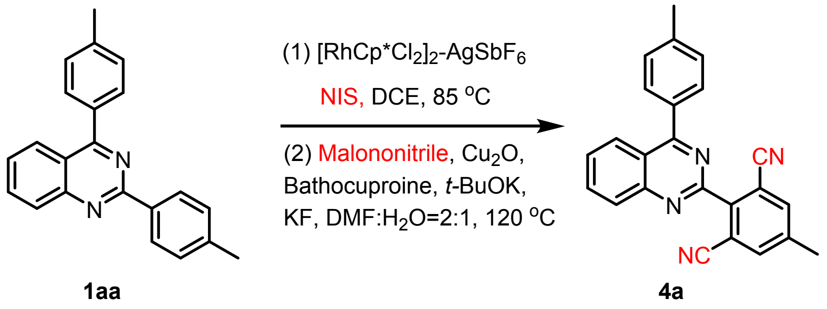

A mixture of 2,4-phenylquinazolines **1aa** (0.2 mmol, 1.0 eq.), NIS (0.6 mmol. 3.0 eq.), $[RhCp^*Cl_2]_2$ (2.0 mol%), $AgSbF_6$(16 mol%) and in DCE (2.0 mL) was stirred at 85°C under for 0.5 h, until **1aa** was completed consumed. The solvent was removed under reduced pressure. A mixture of malononitrile (4.0 eq.), Cu_2O (20 mol%), Bathocuproine (40 mol%), t-BuOK (4.0 eq.), KF (4.0 eq.) in DMF and water (3.0 mL, 2:1) was stirred at 120°C. After completion of the reaction as indicated by TLC, the mixture was cooled to room temperature. The solvent was evaporated, residue was diluted with EtOAc (10 mL), washed with H_2O (10 mL), dried by anhydrous Na_2SO_4. Evaporation of the solvent followed purification by column chromatograph over silica gel provided the corresponding product **4a**.

^{1}H and ^{13}C NMR spectra of the products

2-(2-cyano-6-methylphenyl)-4-(p-tolyl)quinazoline(3a)

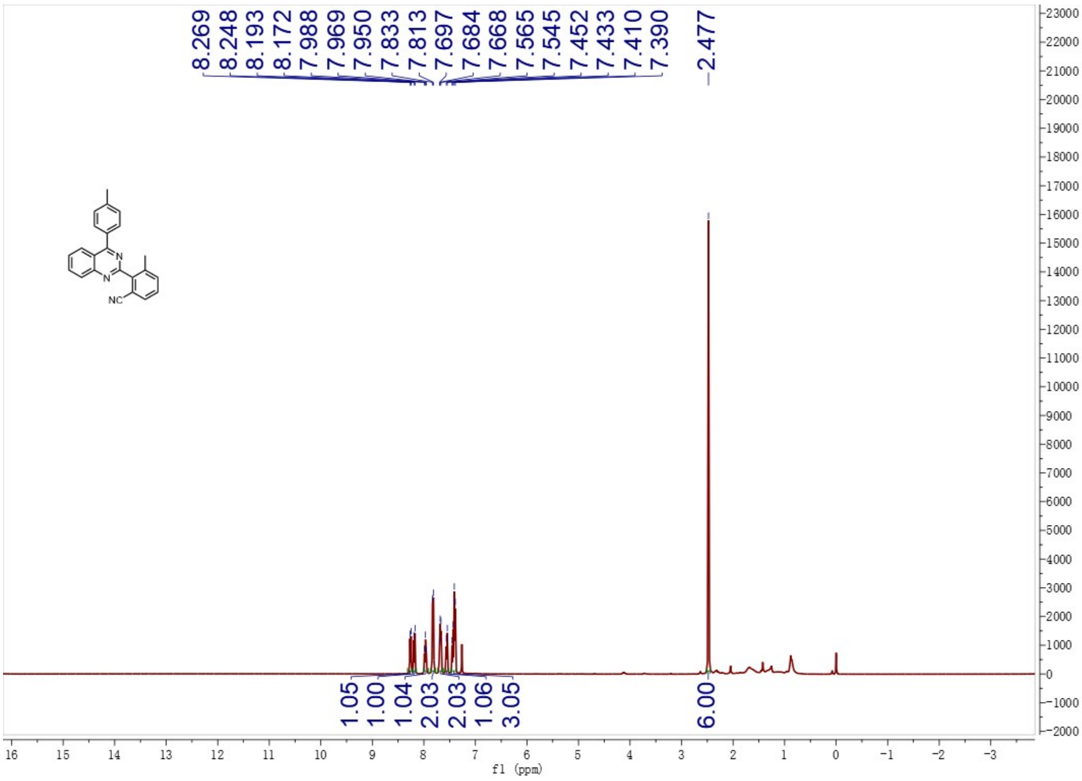

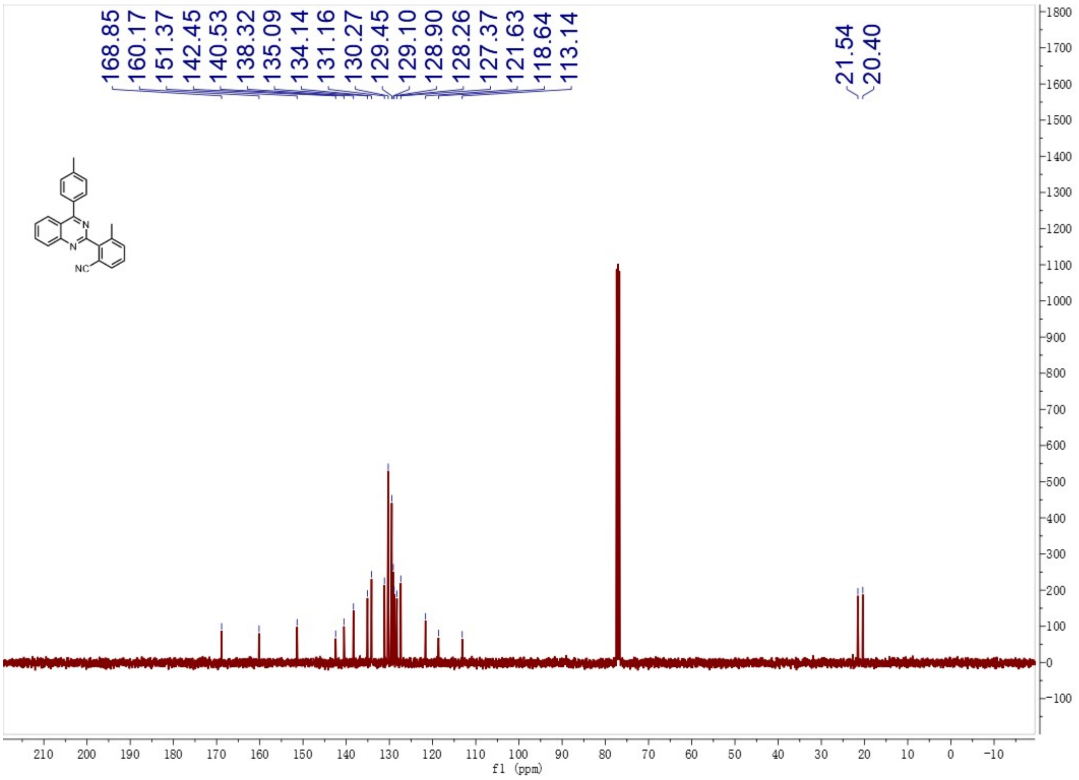

2-(2-cyano-6-methylphenyl)-4-(4-methoxyphenyl)quinazoline(3b)

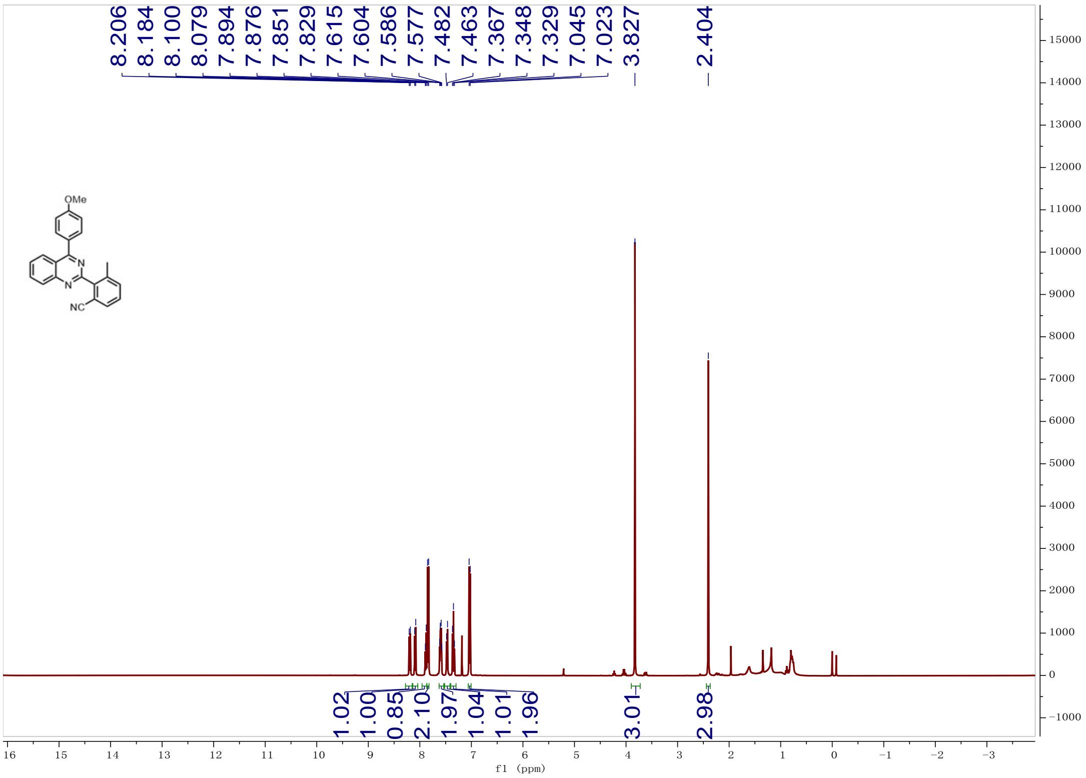

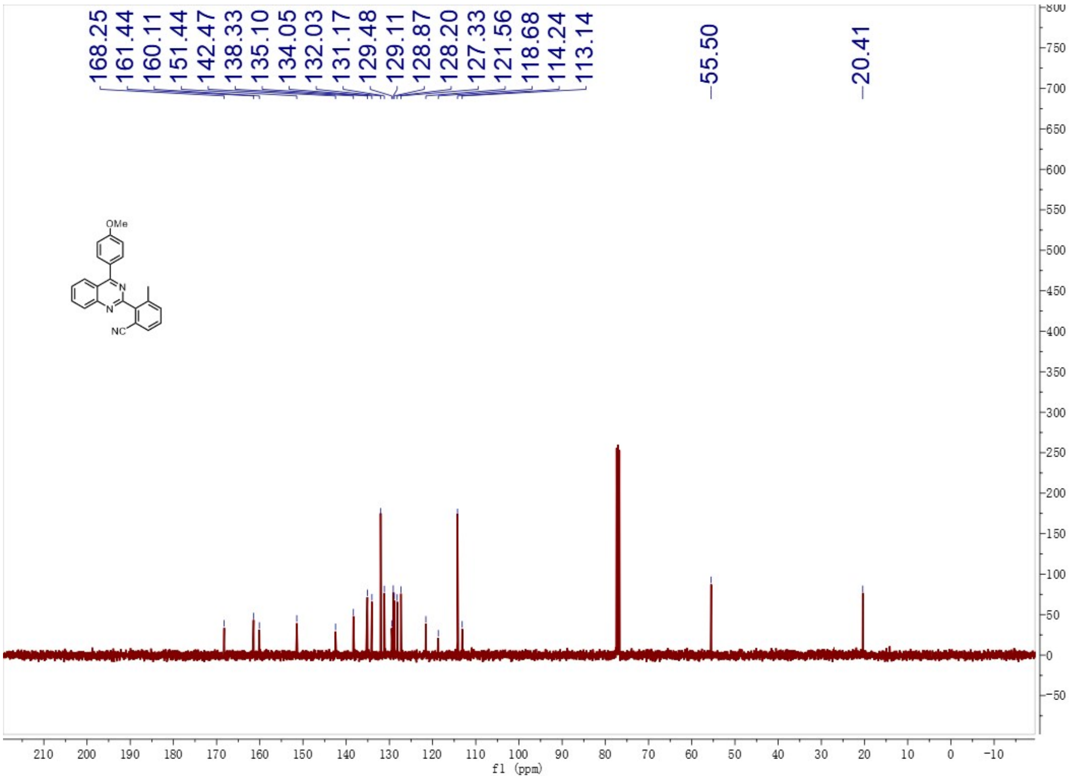

2-(2-cyano-6-methylphenyl)-4-phenylquinazoline (3c)

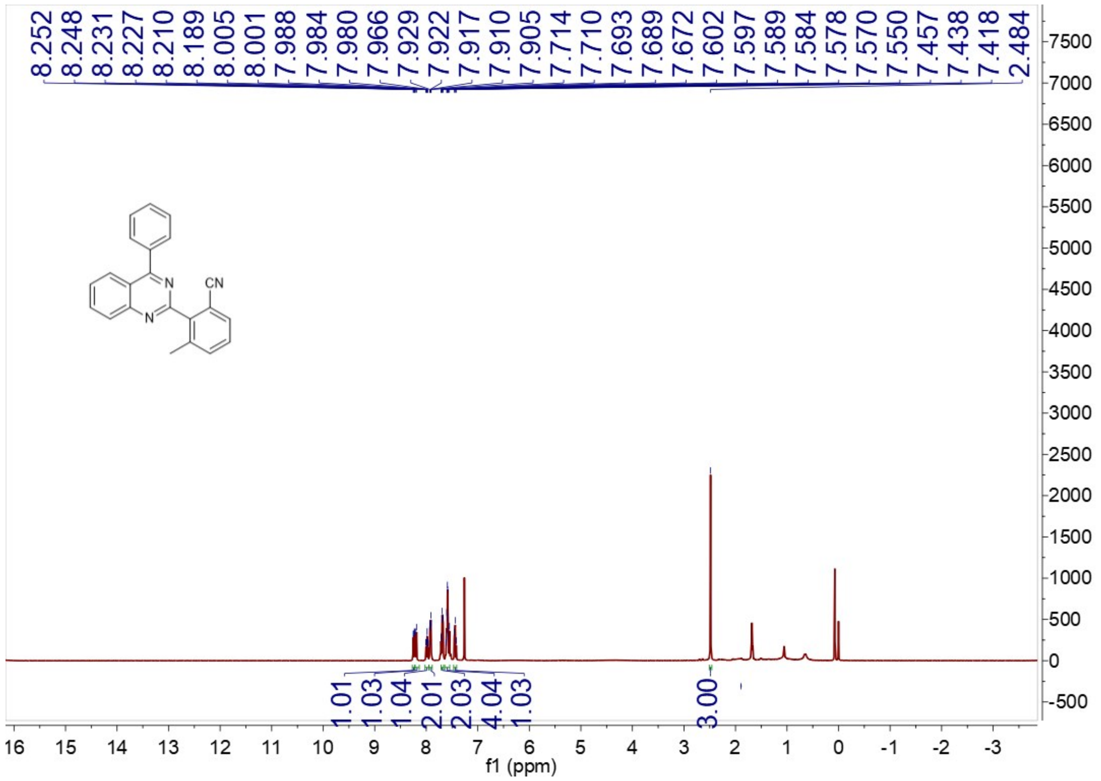

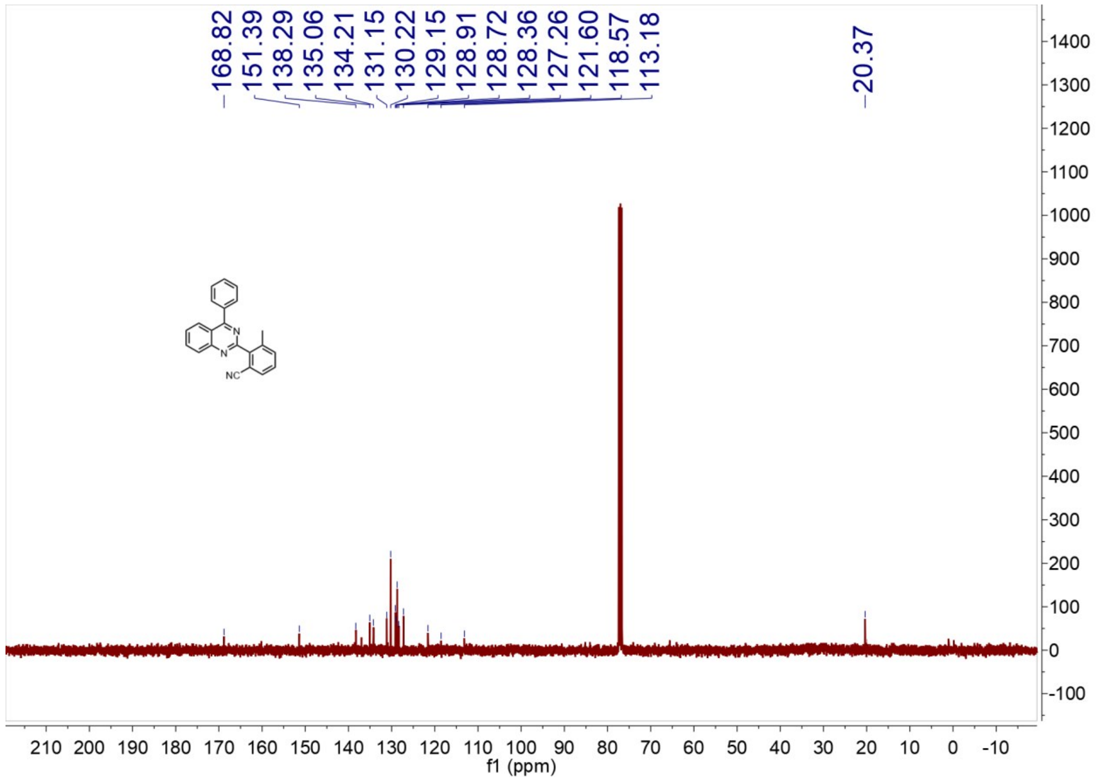

2-(2-cyano-6-methylphenyl)-4-(4-fluorophenyl)quinazoline (3d)

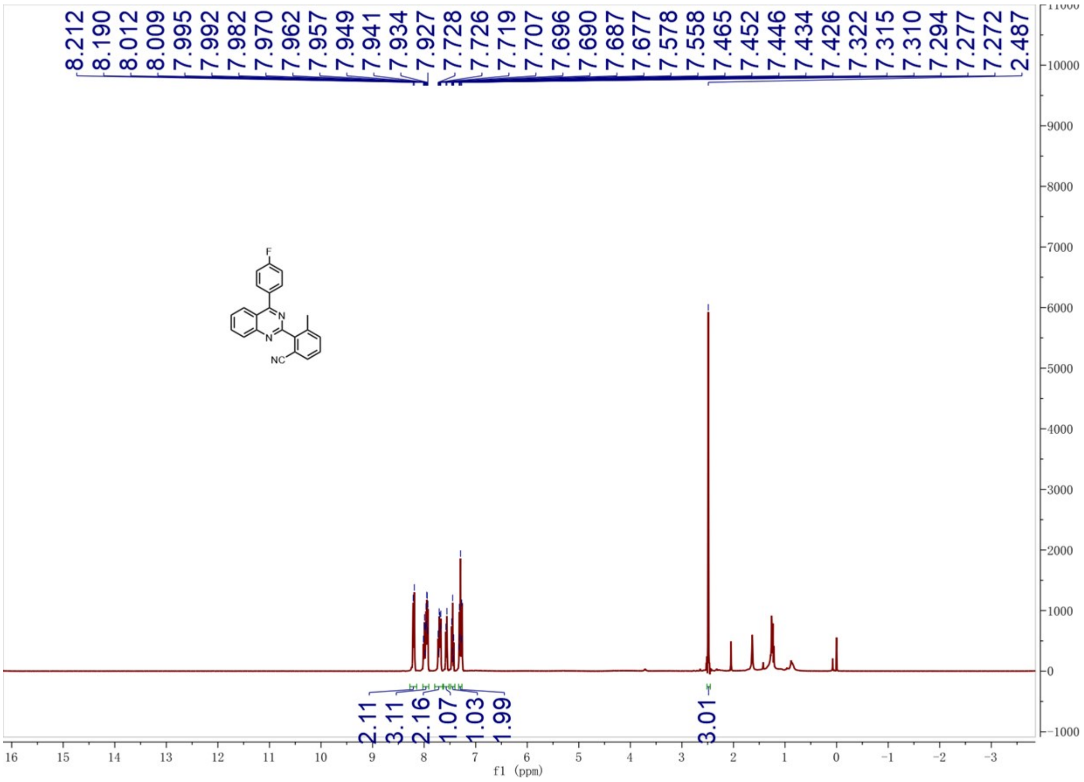

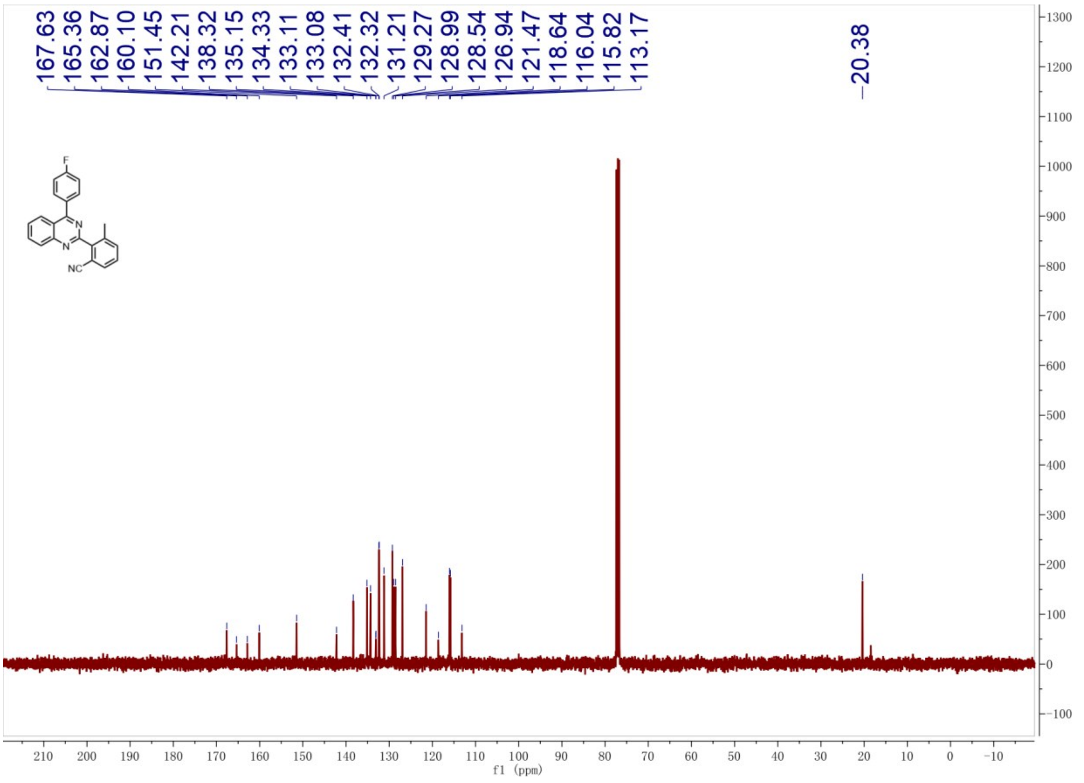

2-(2-cyano-6-methylphenyl)-4-(3-methylphenyl)quinazoline(3e)

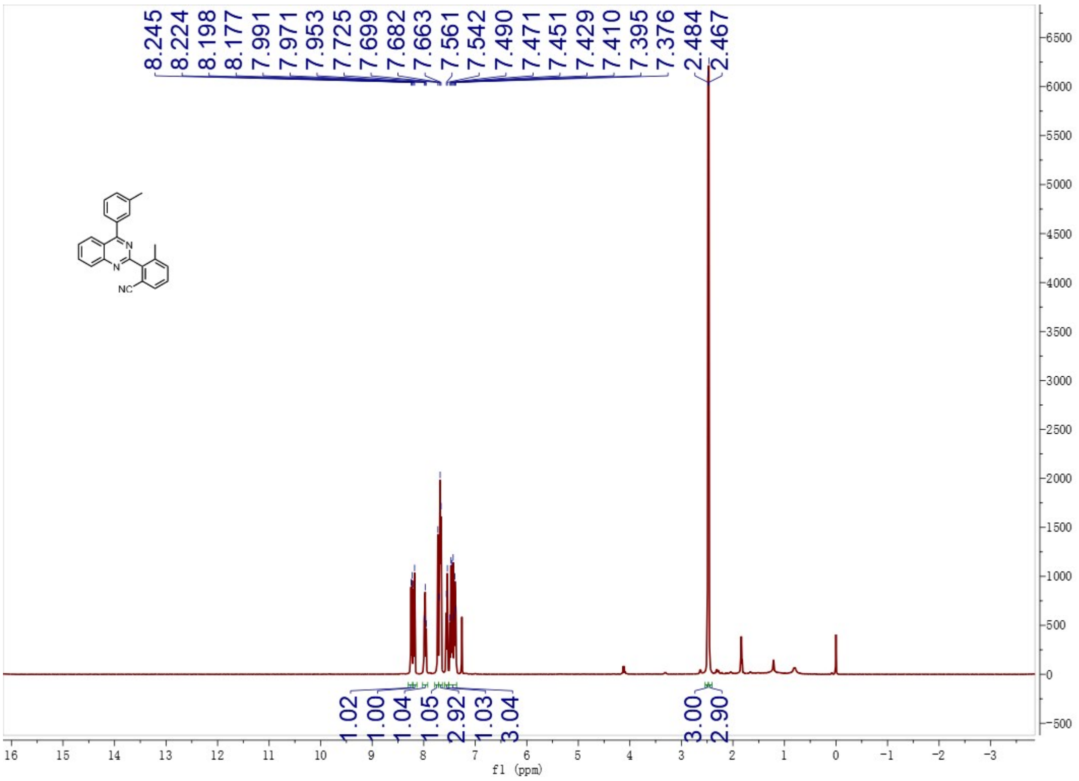

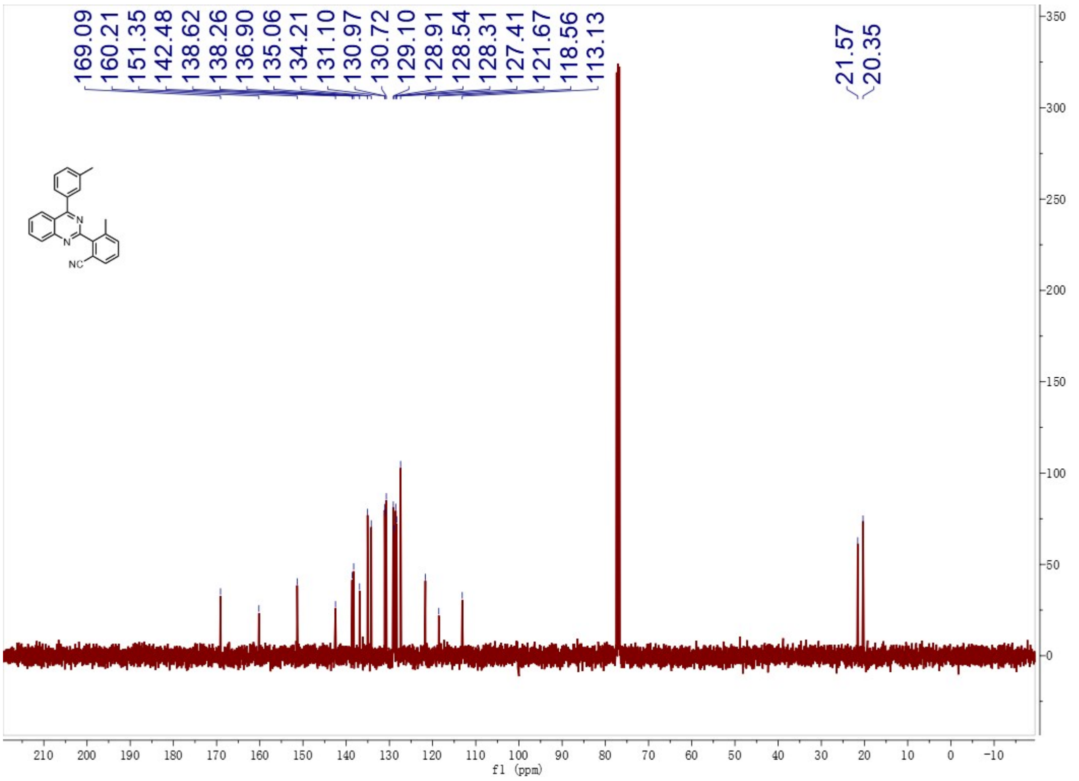

2-(2-cyano-6-methylphenyl)-4-(2-methylphenyl)quinazoline(3f)

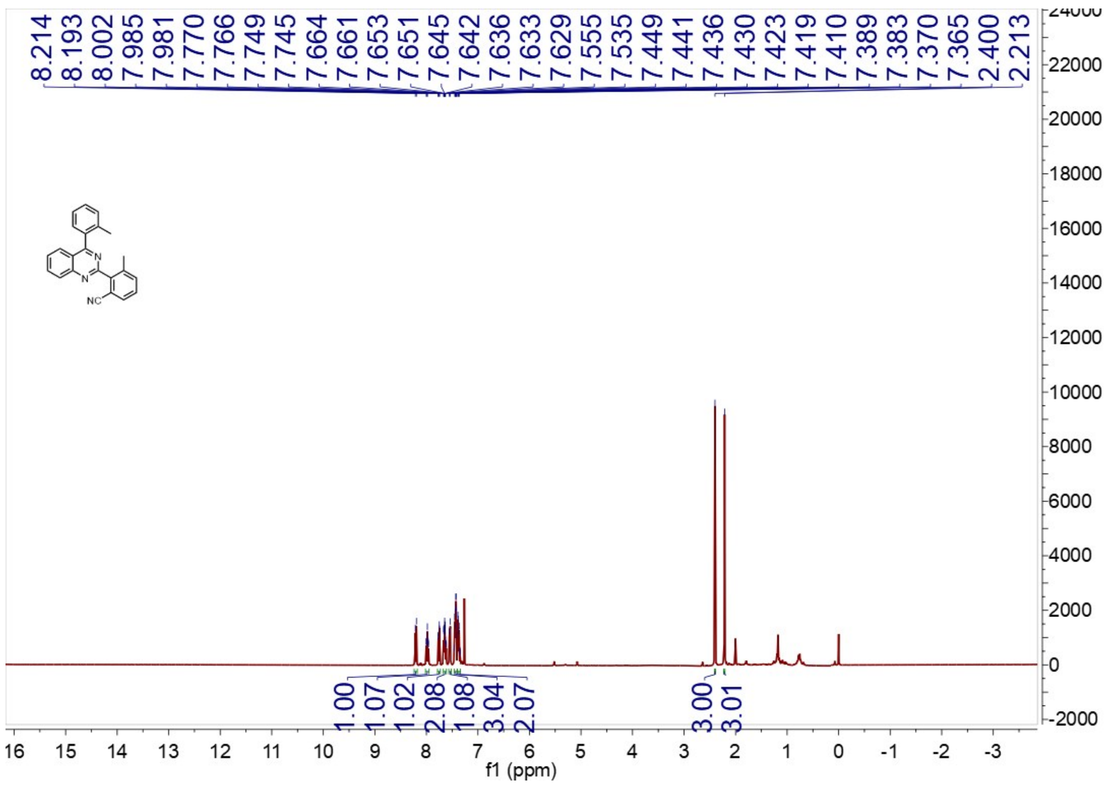

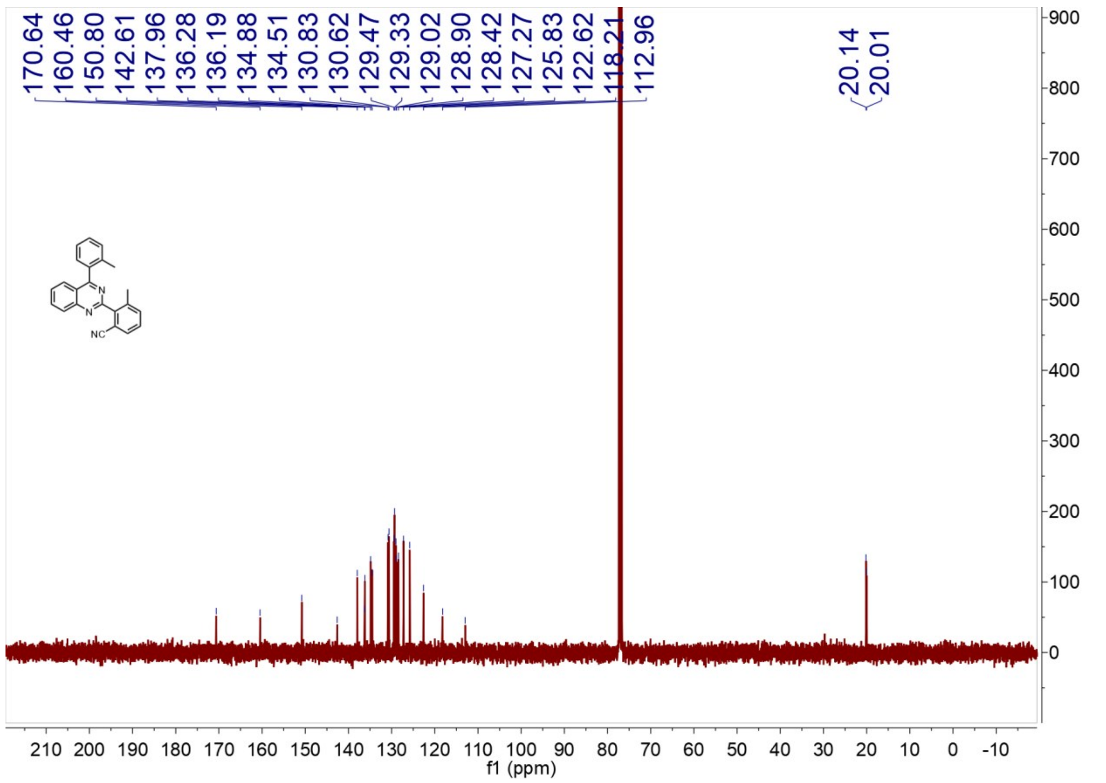

2-(2-cyano-6-methylphenyl)-4-(naphthalen-1-yl)quinazoline (3g)

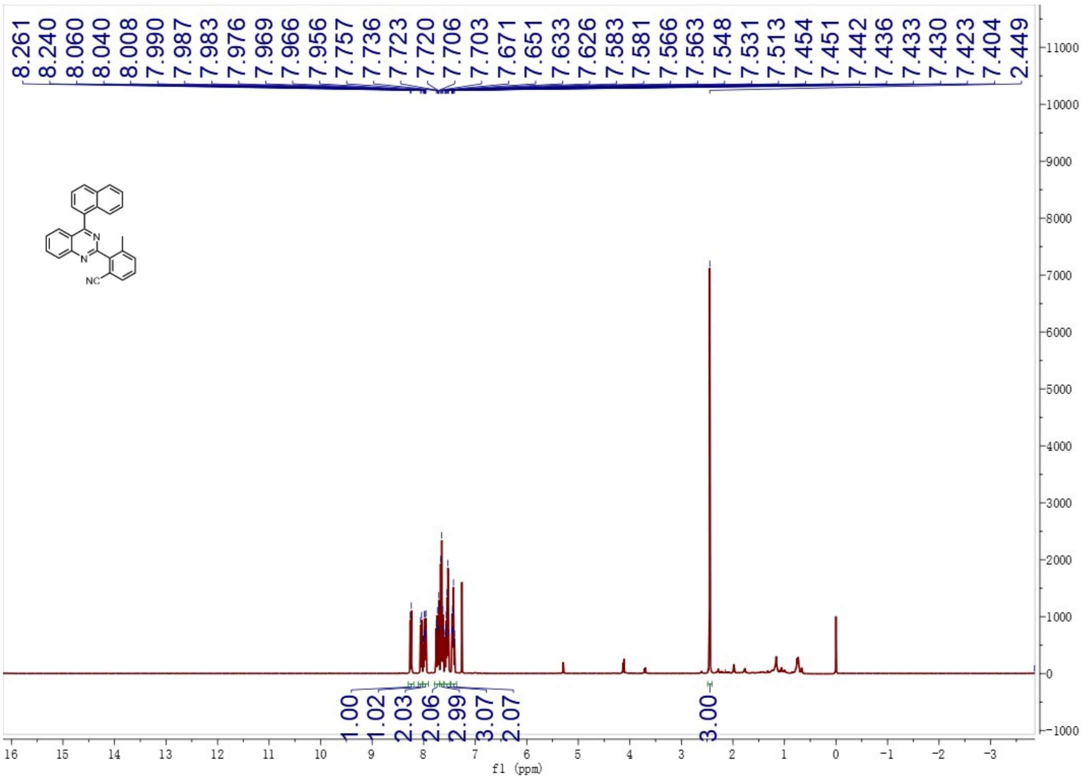

8.261
8.240
8.060
8.040
8.008
7.990
7.987
7.983
7.976
7.969
7.966
7.956
7.757
7.736
7.723
7.720
7.706
7.703
7.671
7.651
7.633
7.626
7.583
7.581
7.566
7.563
7.548
7.531
7.513
7.454
7.451
7.442
7.436
7.433
7.430
7.423
7.404
2.449
1.00
1.02
2.03
2.06
2.99
3.07
2.07
3.00
NC
N
f1 (ppm)

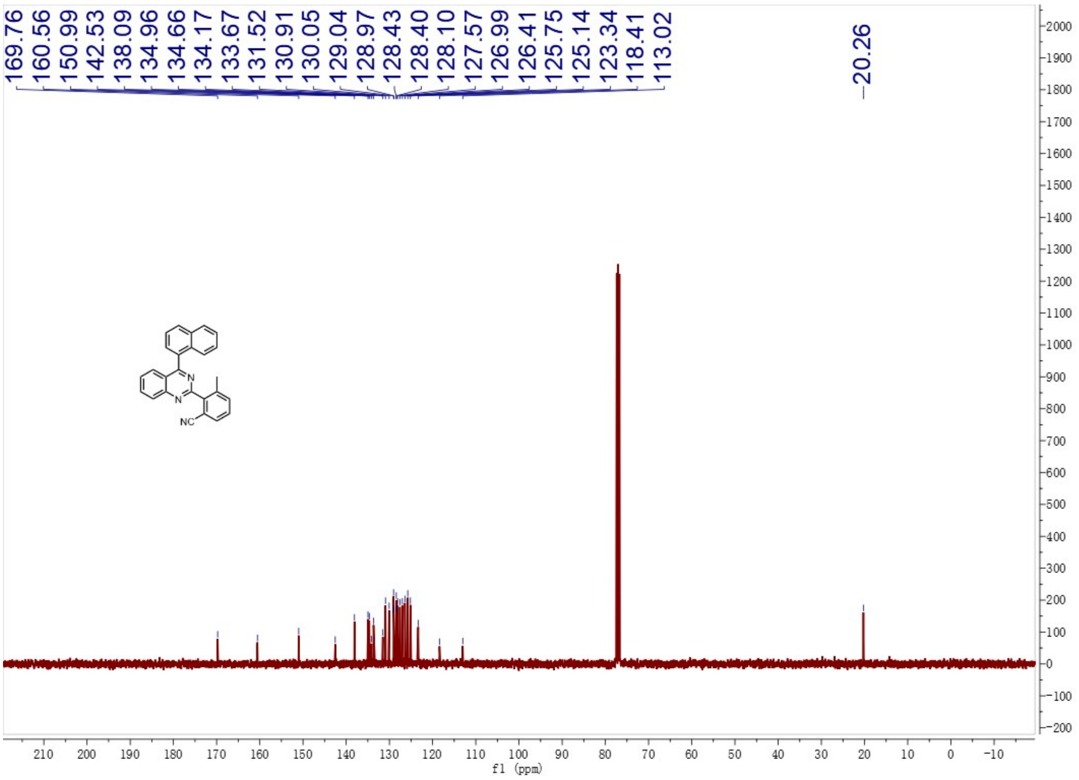

169.76
160.56
150.99
142.53
138.09
134.96
134.66
134.17
133.67
131.52
130.91
130.05
129.04
128.97
128.43
128.40
128.10
127.57
126.99
126.41
125.75
125.14
123.34
118.41
113.02
20.26
NC
N
f1 (ppm)

2-(2-cyano-6-chlorophenyl)-4-(p-tolyl)quinazoline (3h)

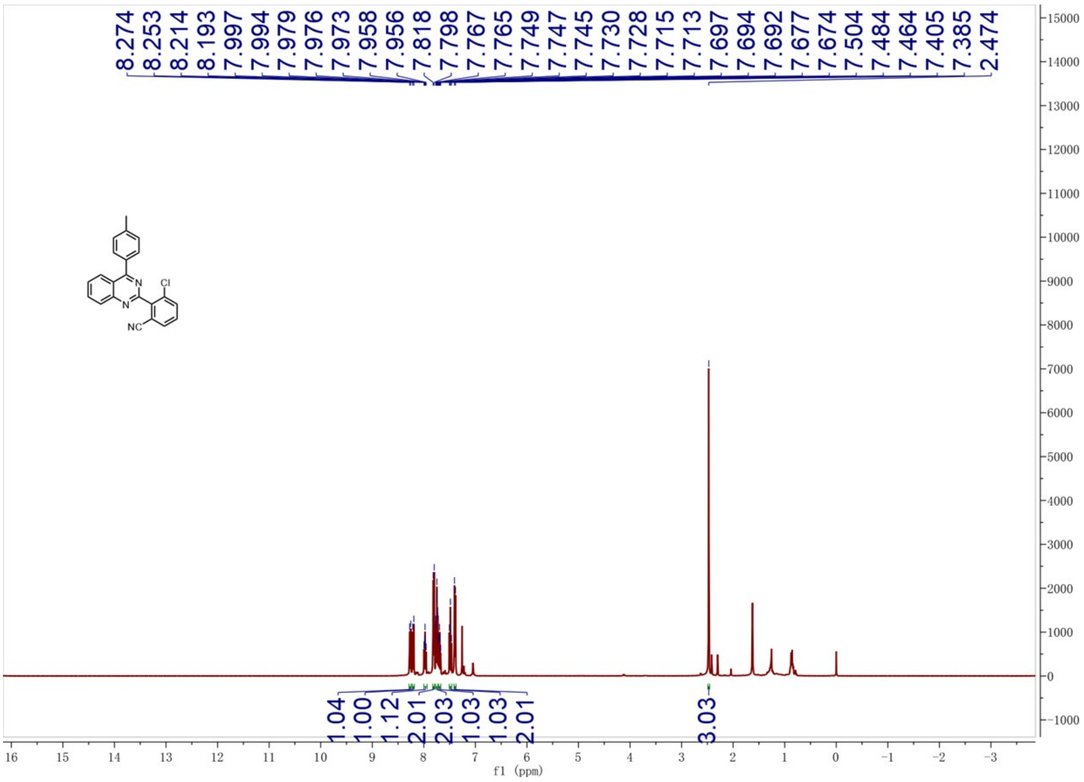

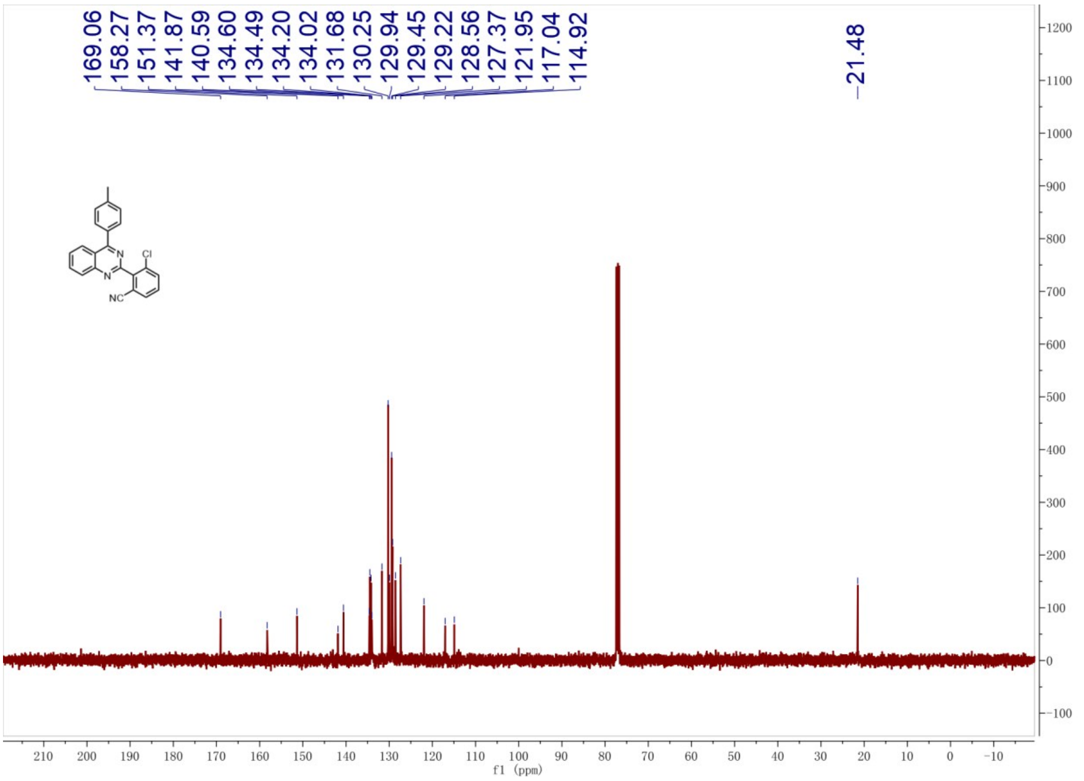

2-(2-cyano-6-chlorophenyl)-4-(3-methylphenyl)quinazoline (3i)

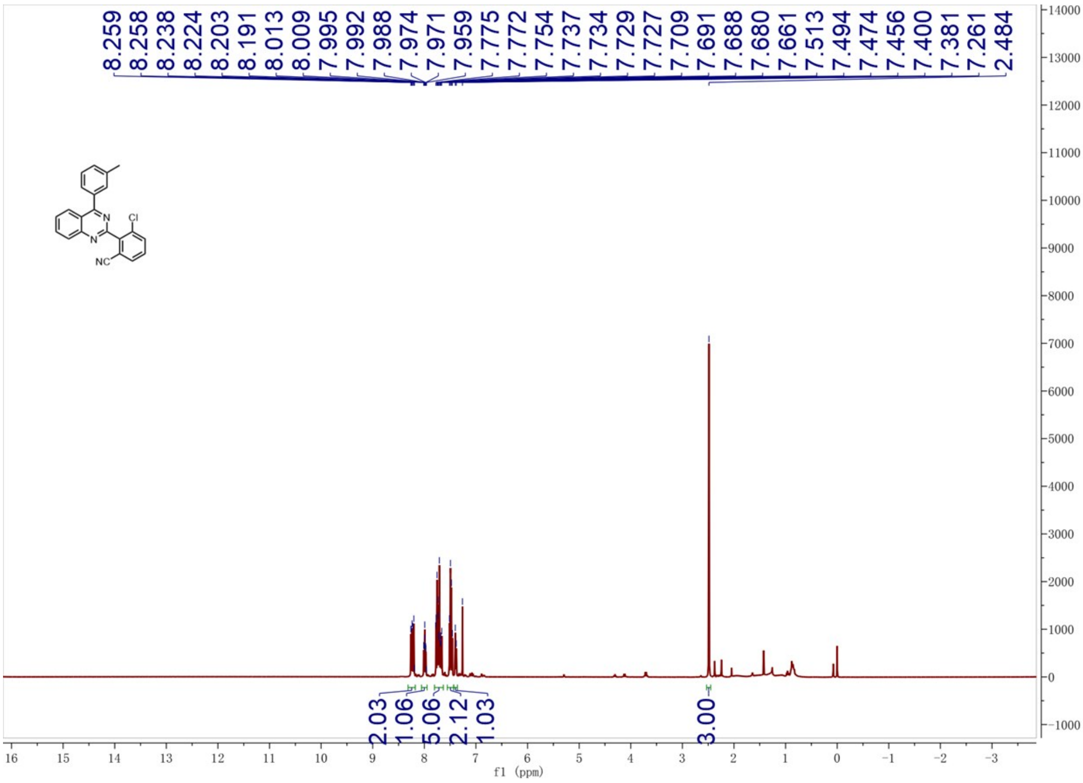

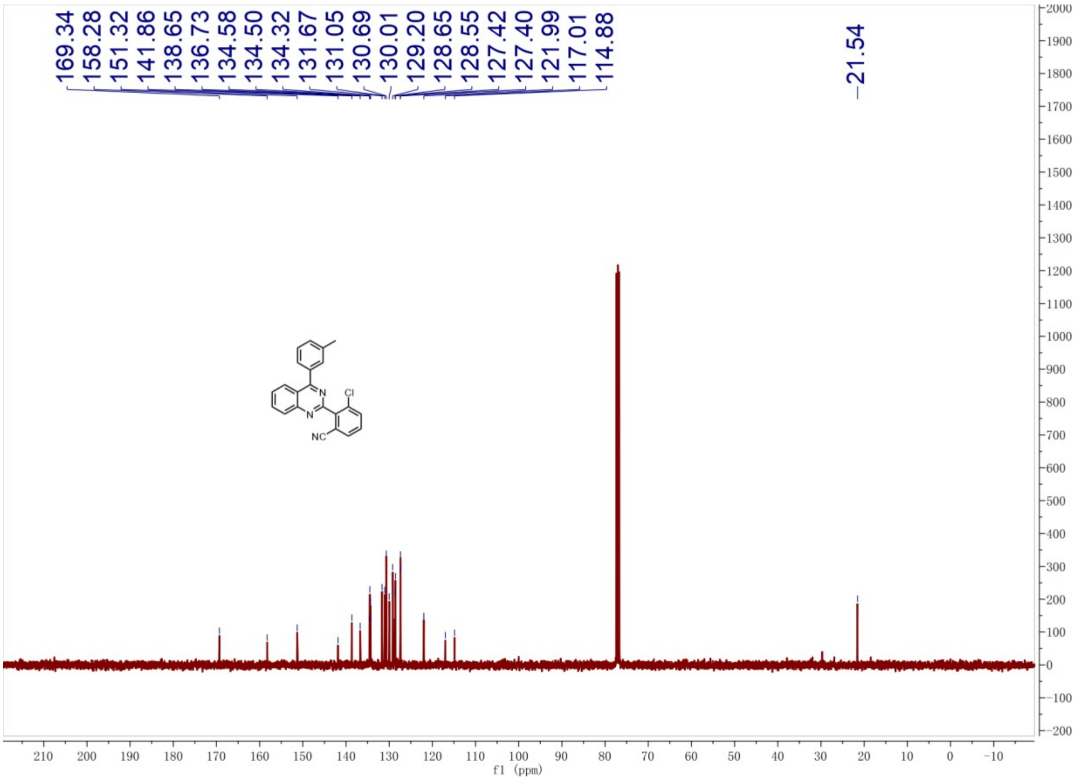

2-(2-cyano-6-chlorophenyl)-4-(2-methylphenyl)quinazoline(3j)

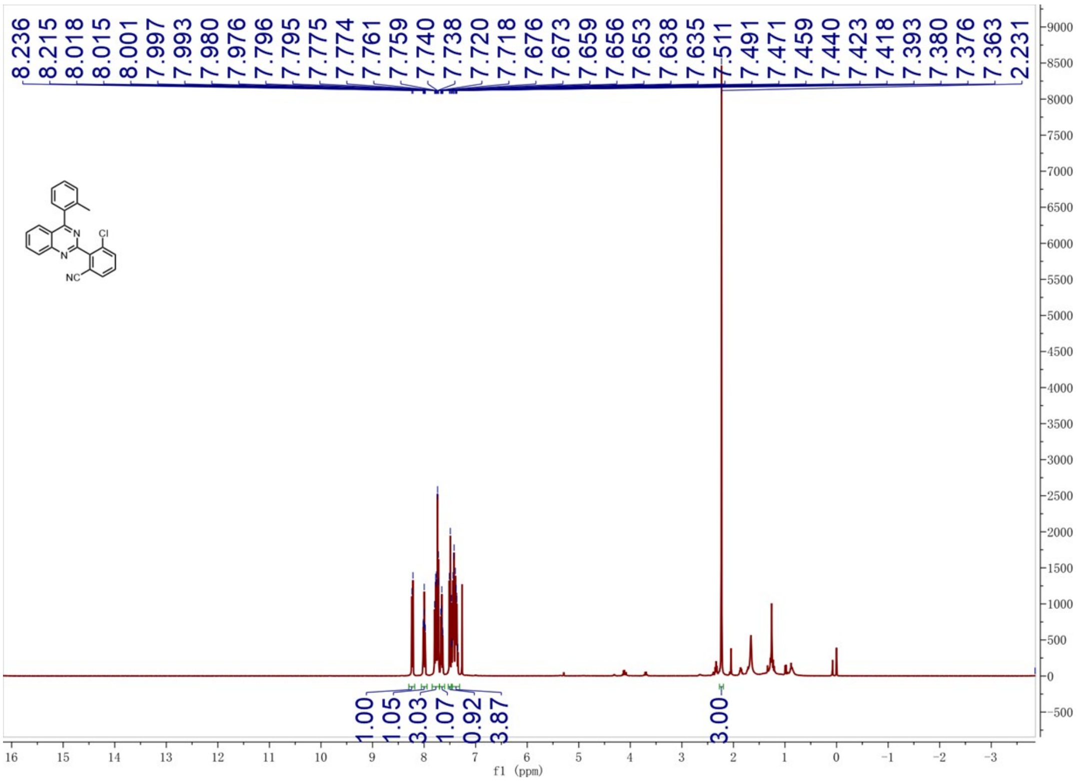

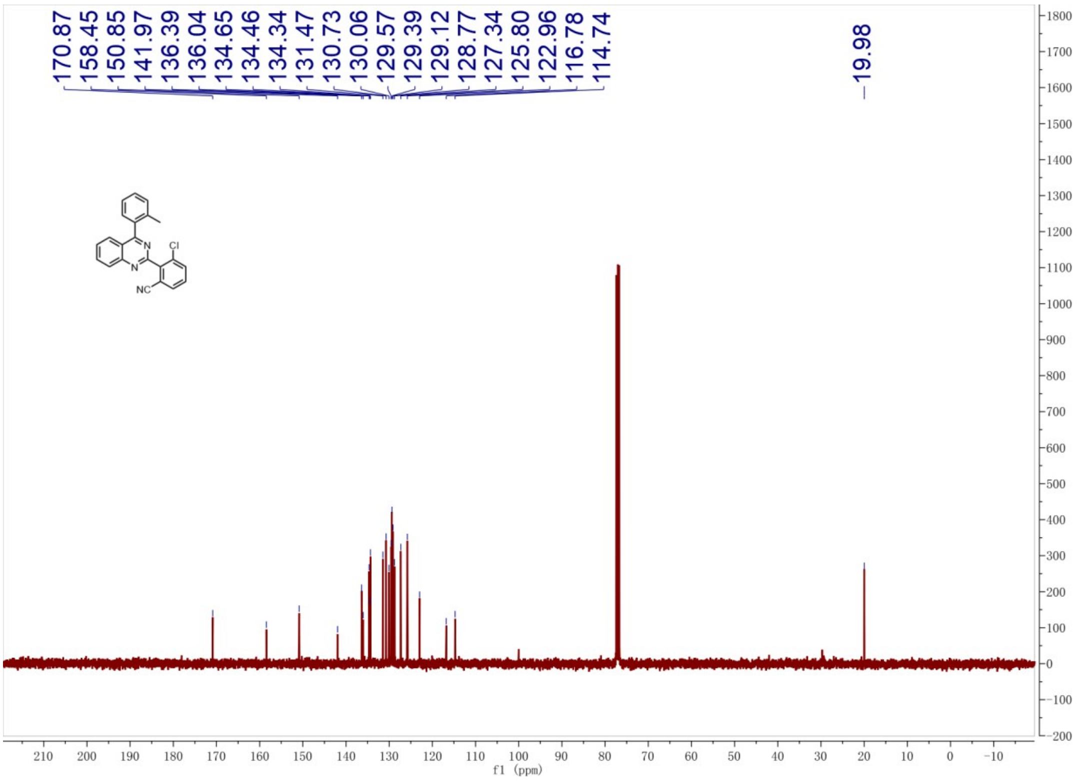

2-(2-cyano-6-methylphenyl)-4-(p-tolyl)-6-methoxylquinazoline (3k)

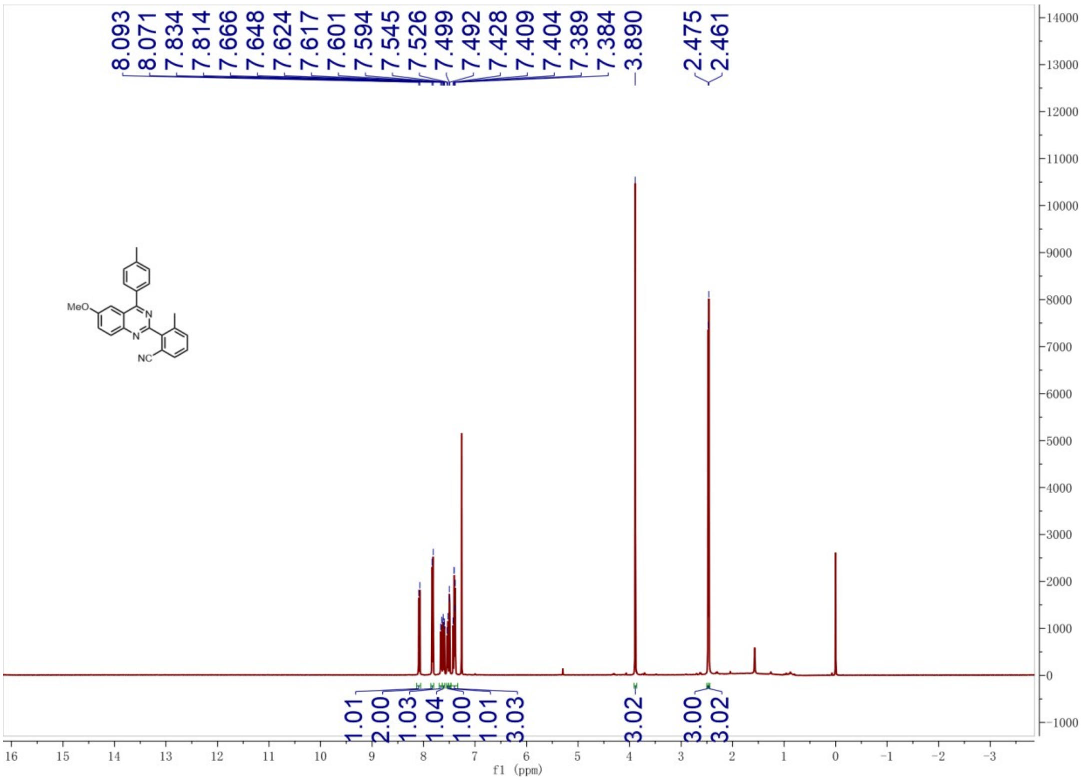

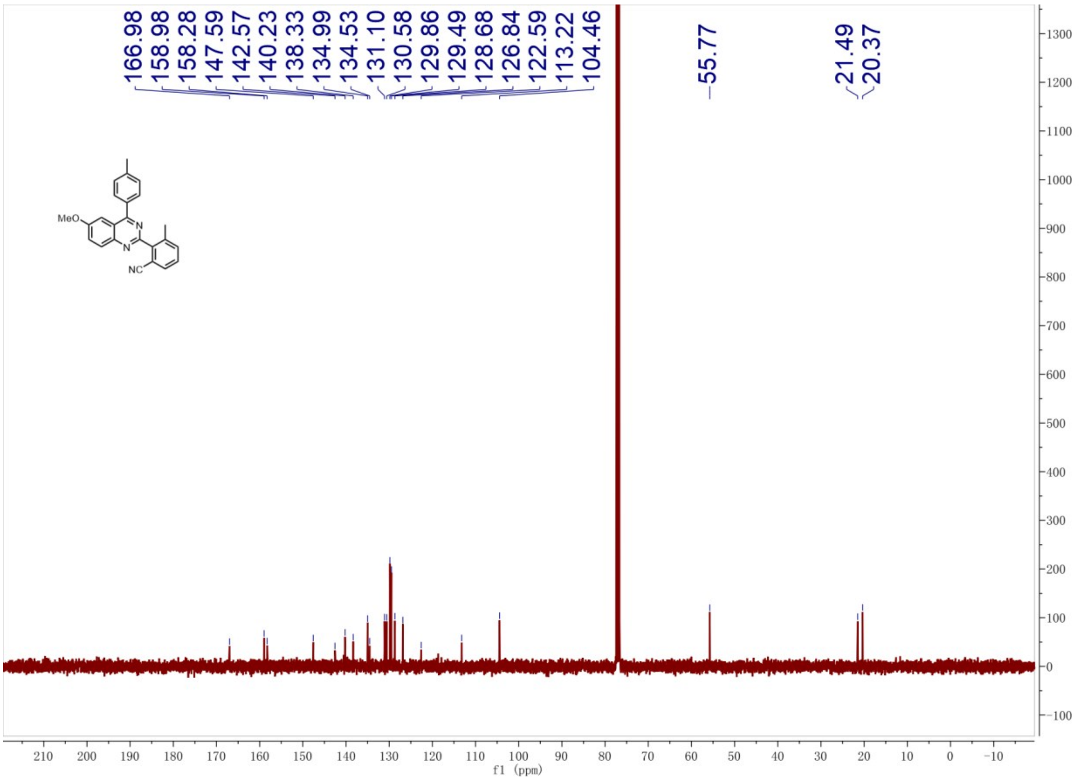

2-(2-cyano-6-methylphenyl)-4-(3-methylphenyl)-6-methoxylquinazoline(3l)

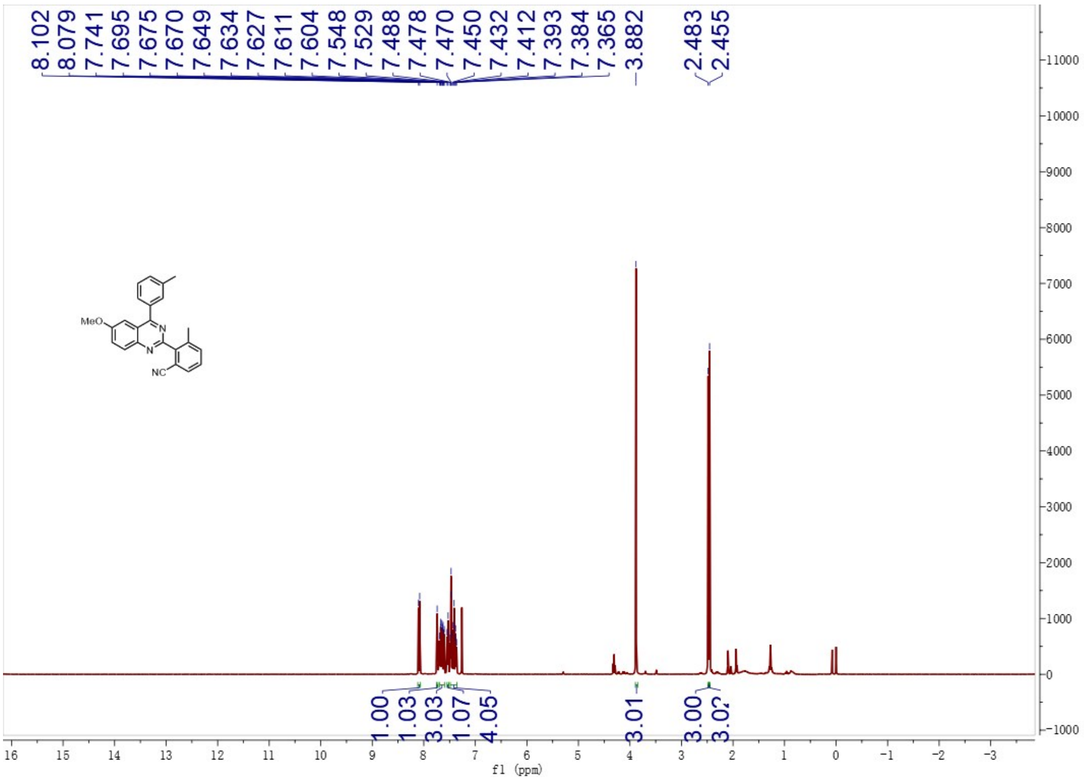

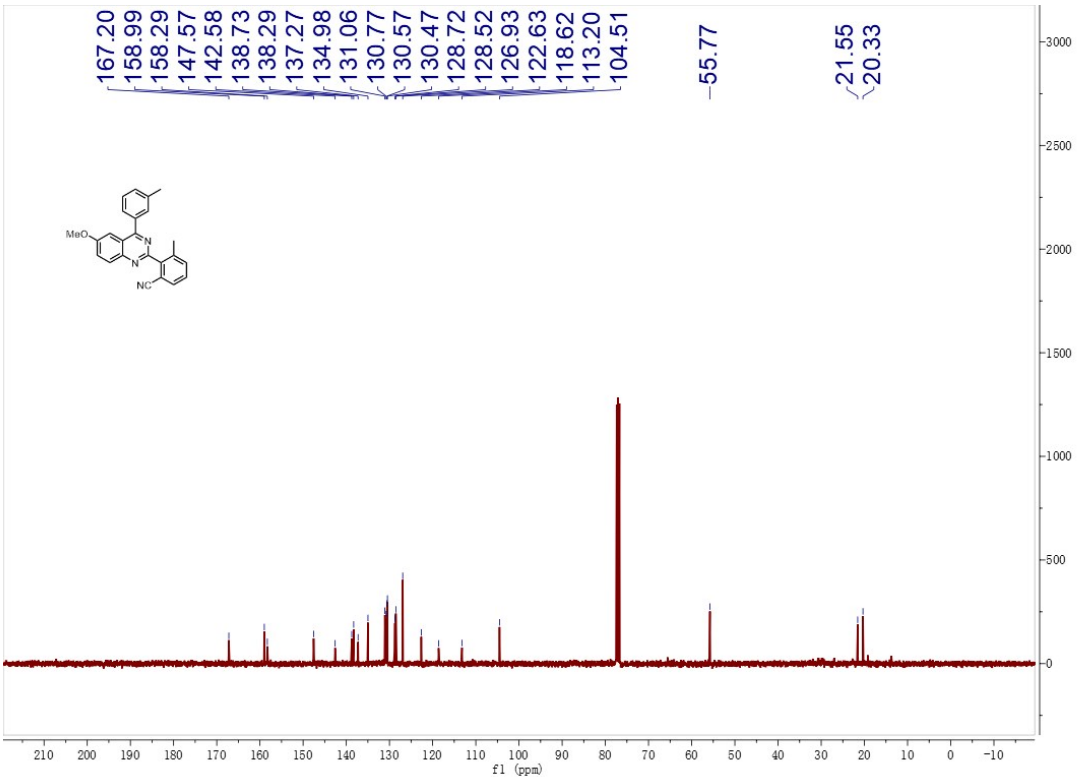

6-chloro-2-(2-cyano-6-methylphenyl)-4-(p-tolyl)quinazoline(3m)

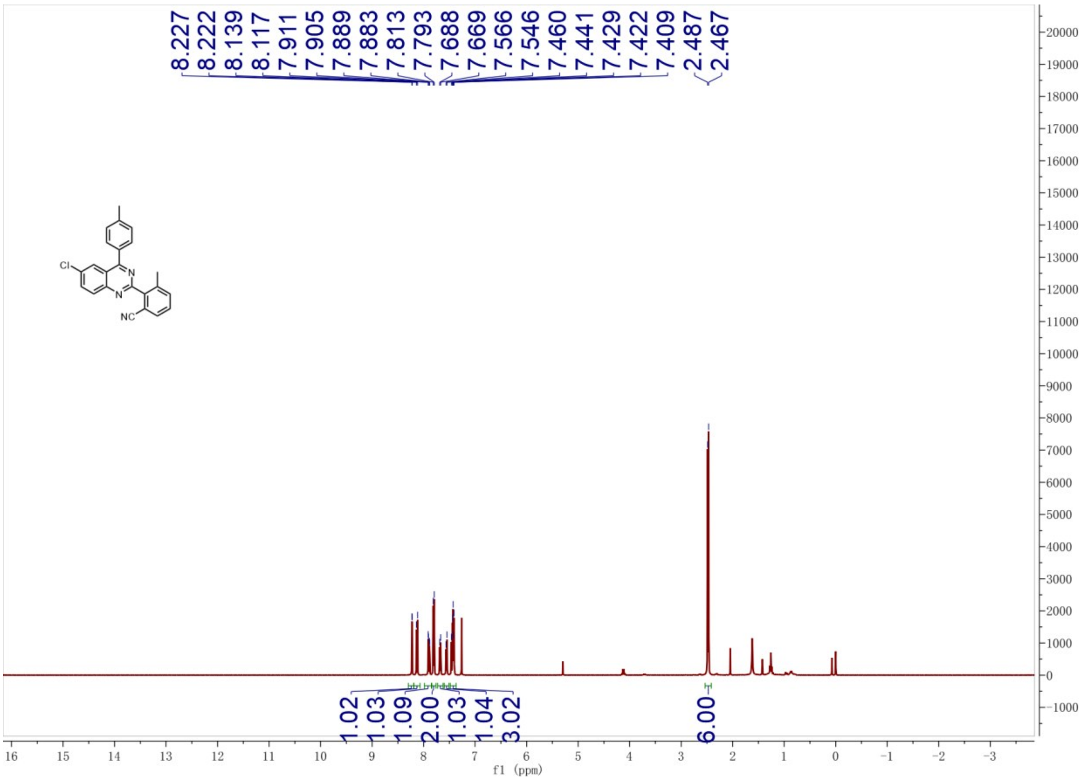

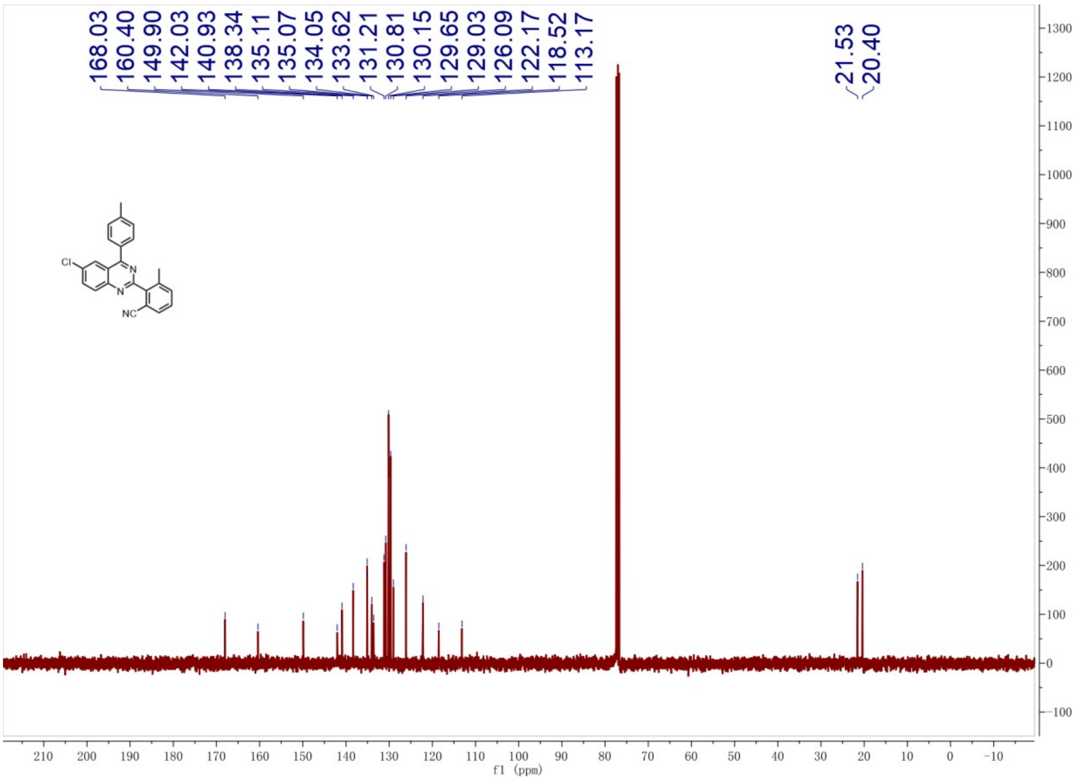

6-chloro-2-(2-cyano-6-methylphenyl)-4-(3-methylphenyl) quinazoline (3n)

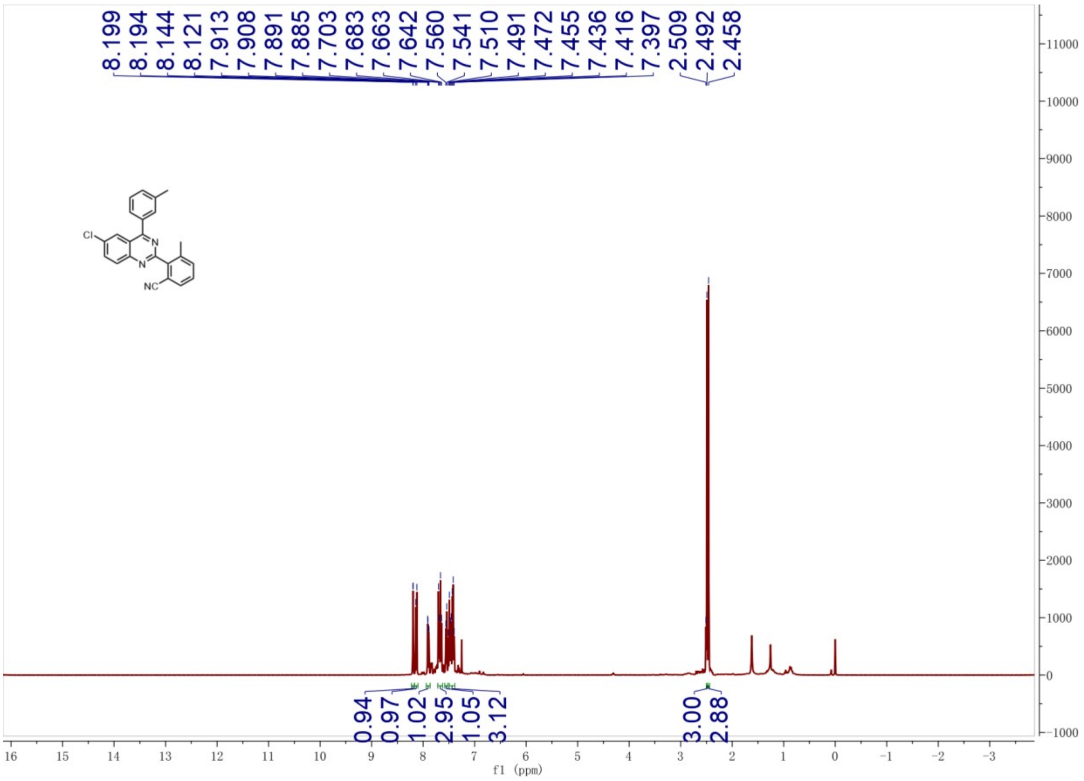

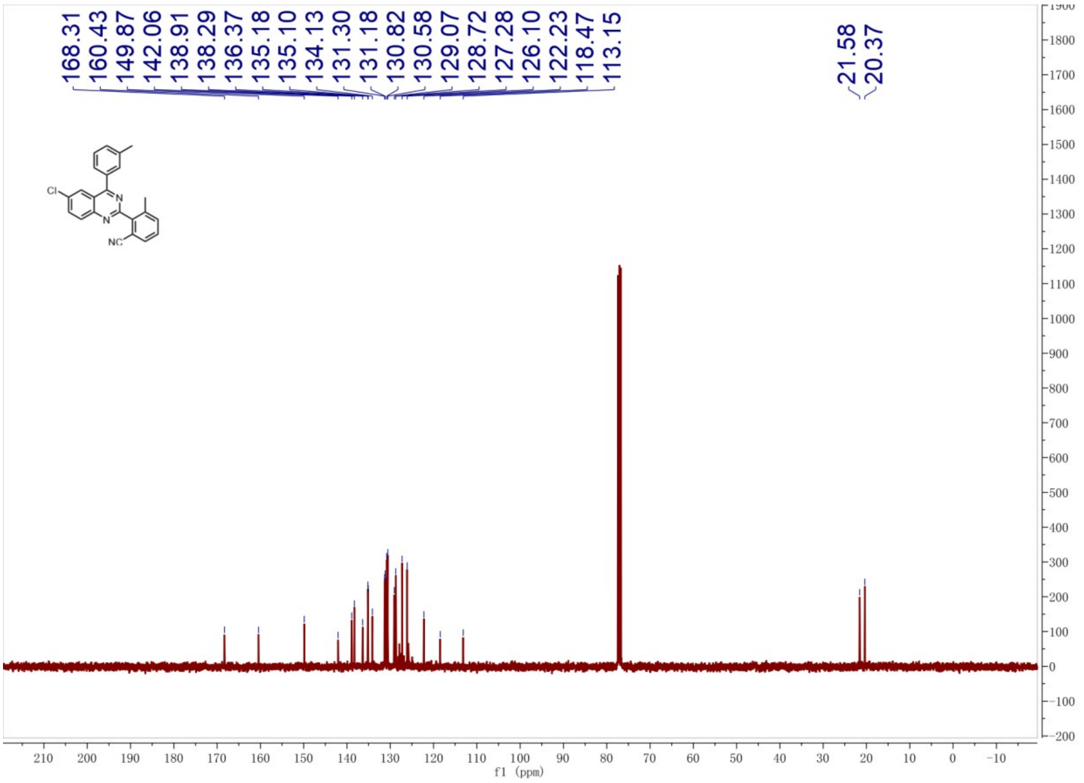

6-chloro-2-(2-cyano-6-methylphenyl)-4-(2-methylphenyl)quinazoline (3o)

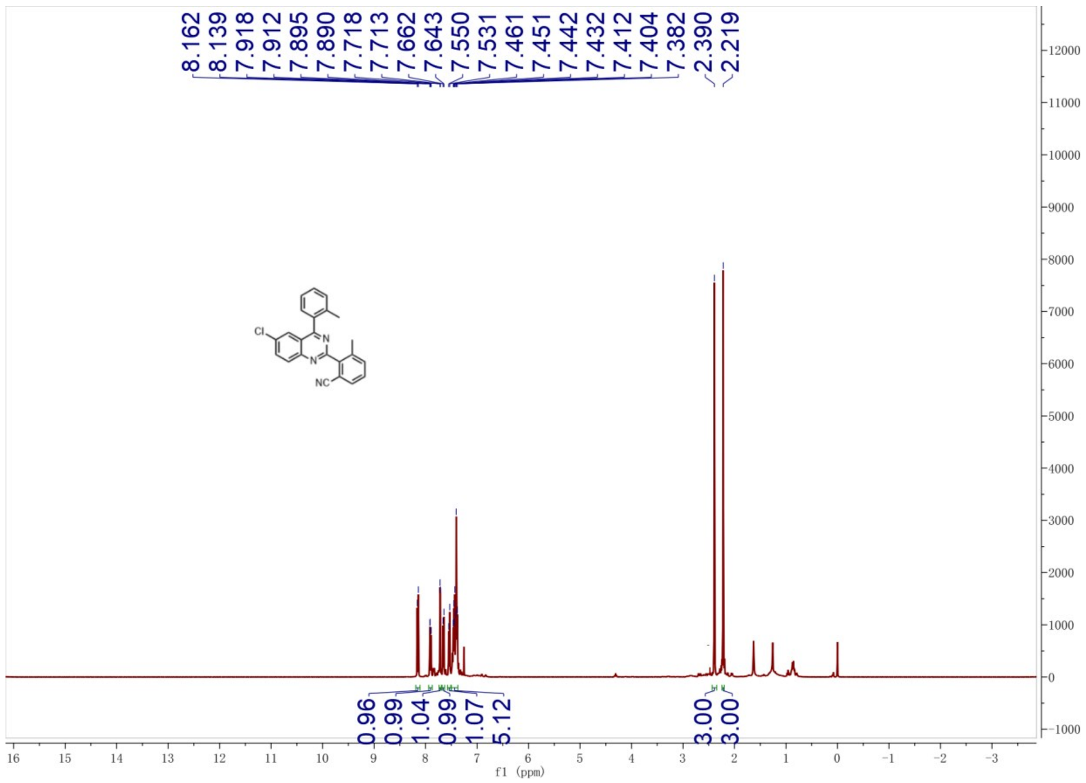

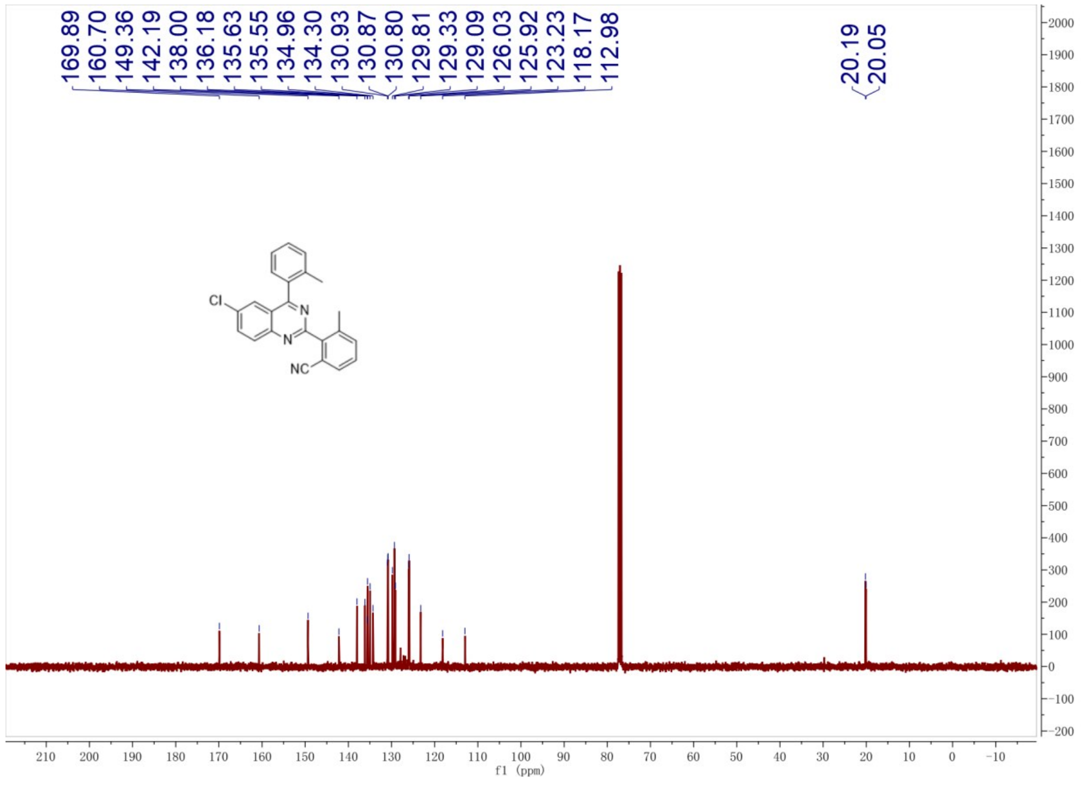

6-chloro-2-(2-cyano-6-methylphenyl)-4-(naphthalen-1-yl) quinazoline (3p)

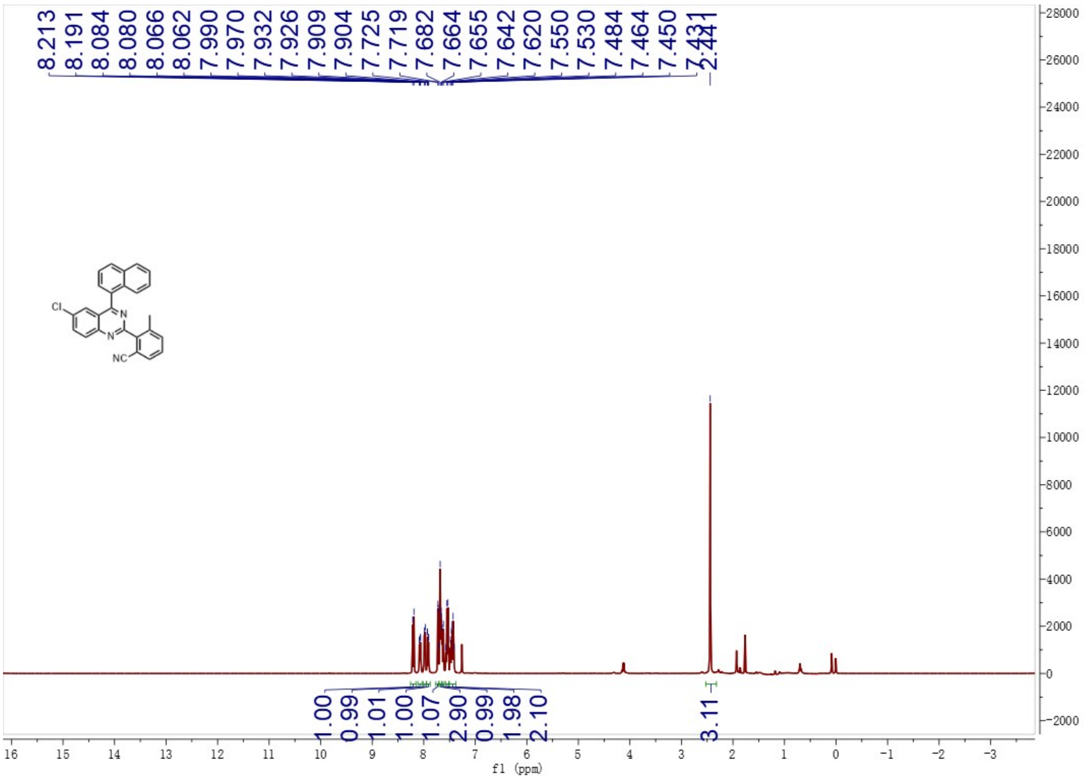

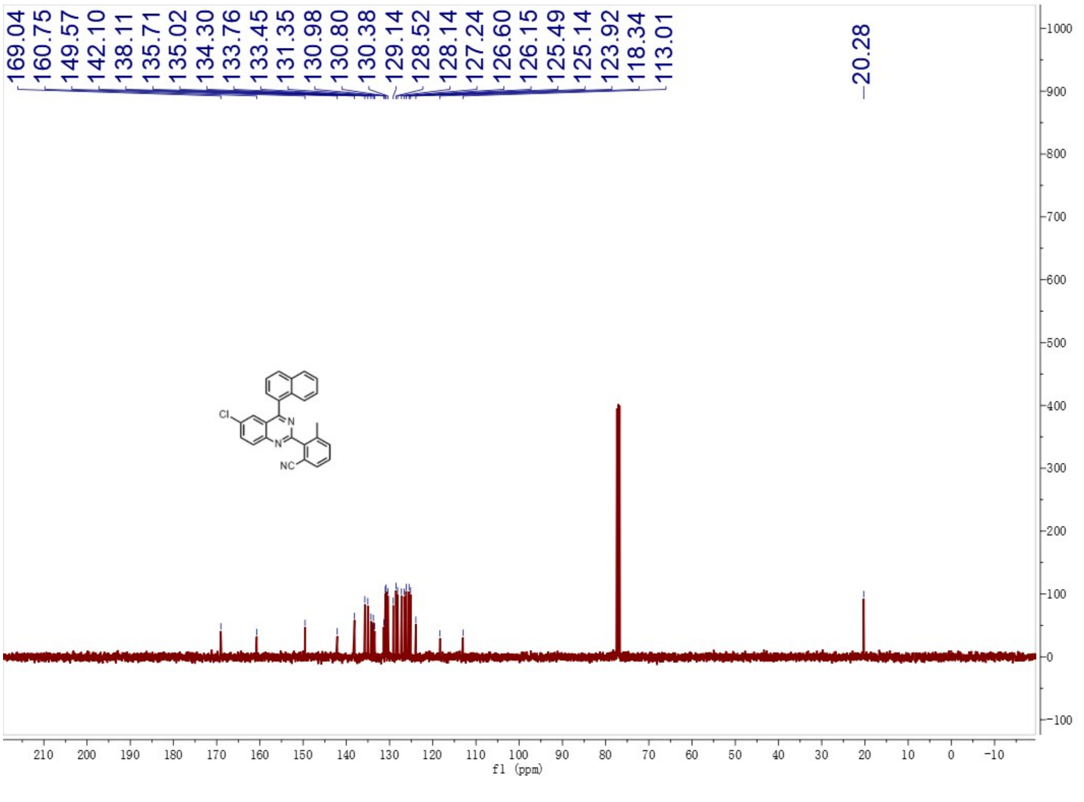

2-(2-cyano-6-chlorophenyl)-4-(2-methylphenyl)-6-methoxylquinazoline (3q)

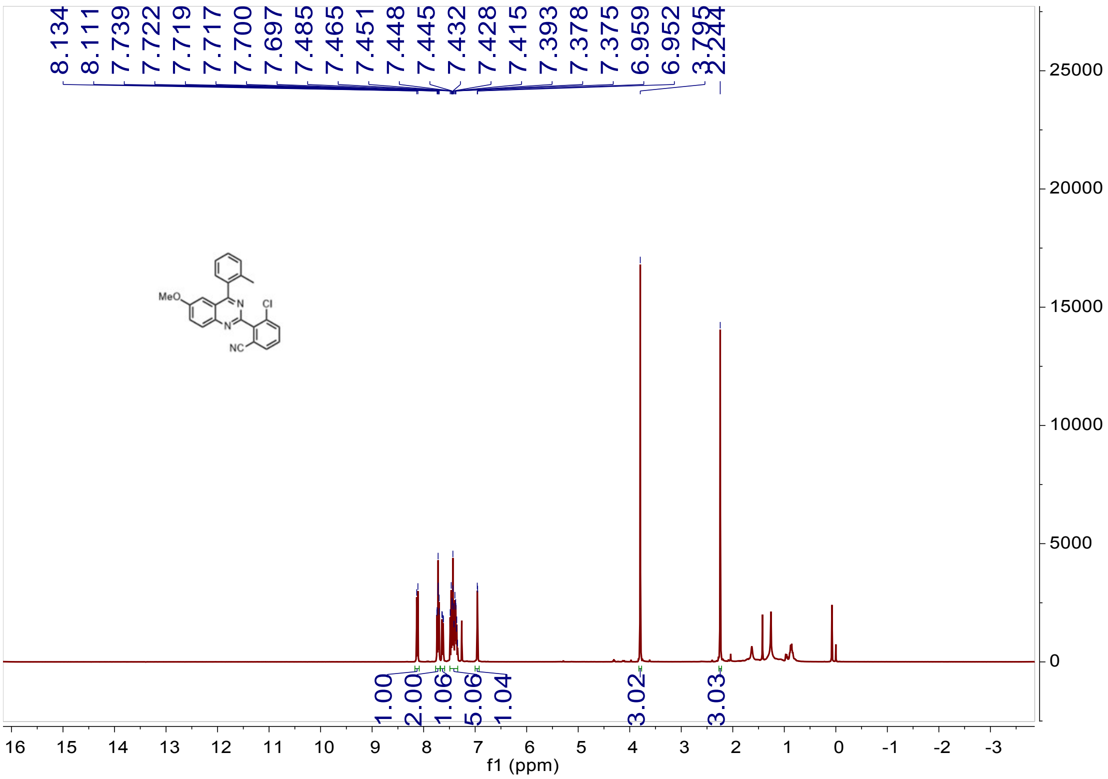

8.134
8.111
7.739
7.722
7.719
7.717
7.700
7.697
7.485
7.465
7.451
7.448
7.445
7.432
7.428
7.415
7.393
7.378
7.375
6.959
6.952
3.795
2.244
MeO
N
N
Cl
NC
1.00
2.00
1.06
5.06
1.04
3.02
3.03
f1 (ppm)

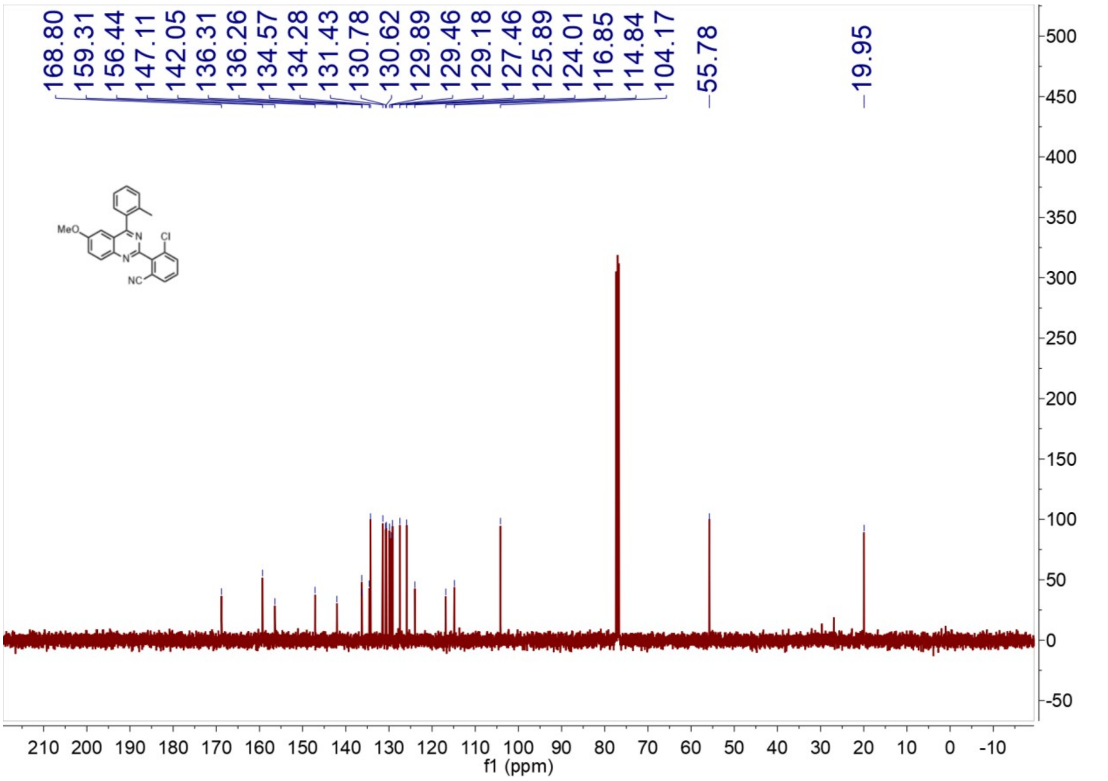

168.80
159.31
156.44
147.11
142.05
136.31
136.26
134.57
134.28
131.43
130.78
130.62
129.89
129.46
129.18
127.46
125.89
124.01
116.85
114.84
104.17
55.78
19.95
MeO
N
N
Cl
NC
f1 (ppm)

6-chloro-2-(2-cyano-6-methylphenyl)-4-(4-fluorophenyl) quinazoline (3r)

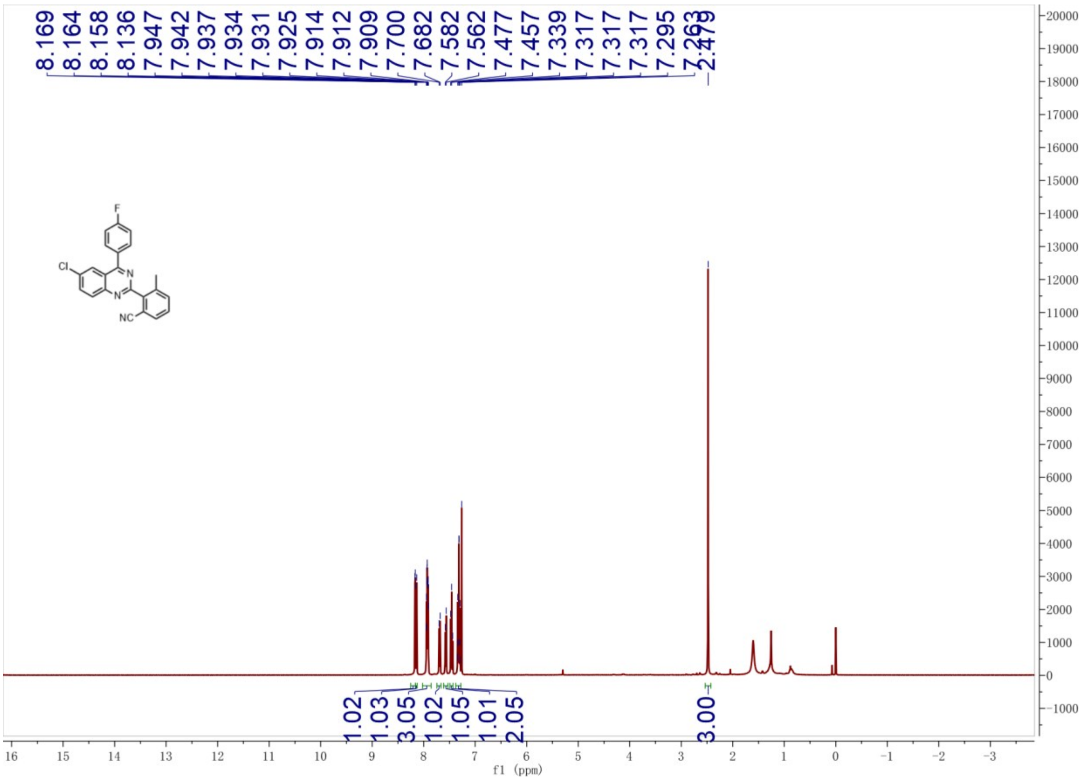

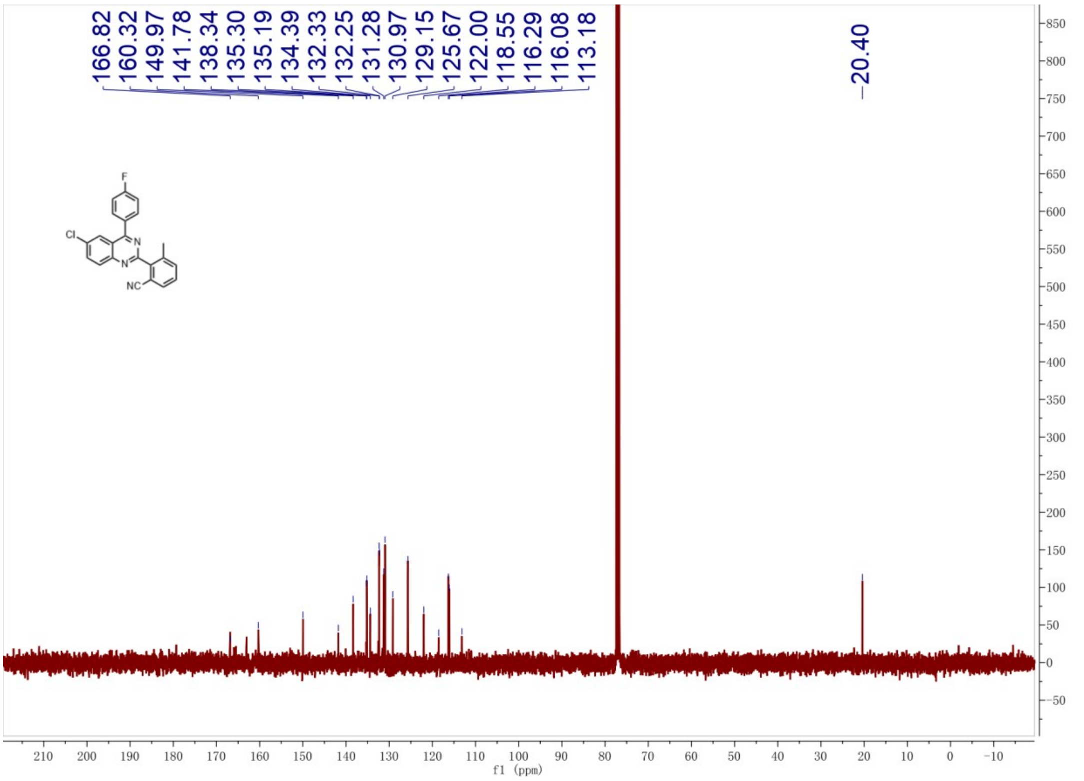

2-(2, 6-dicyano-4-methylphenyl)-4-(p-tolyl)quinazoline (4a)

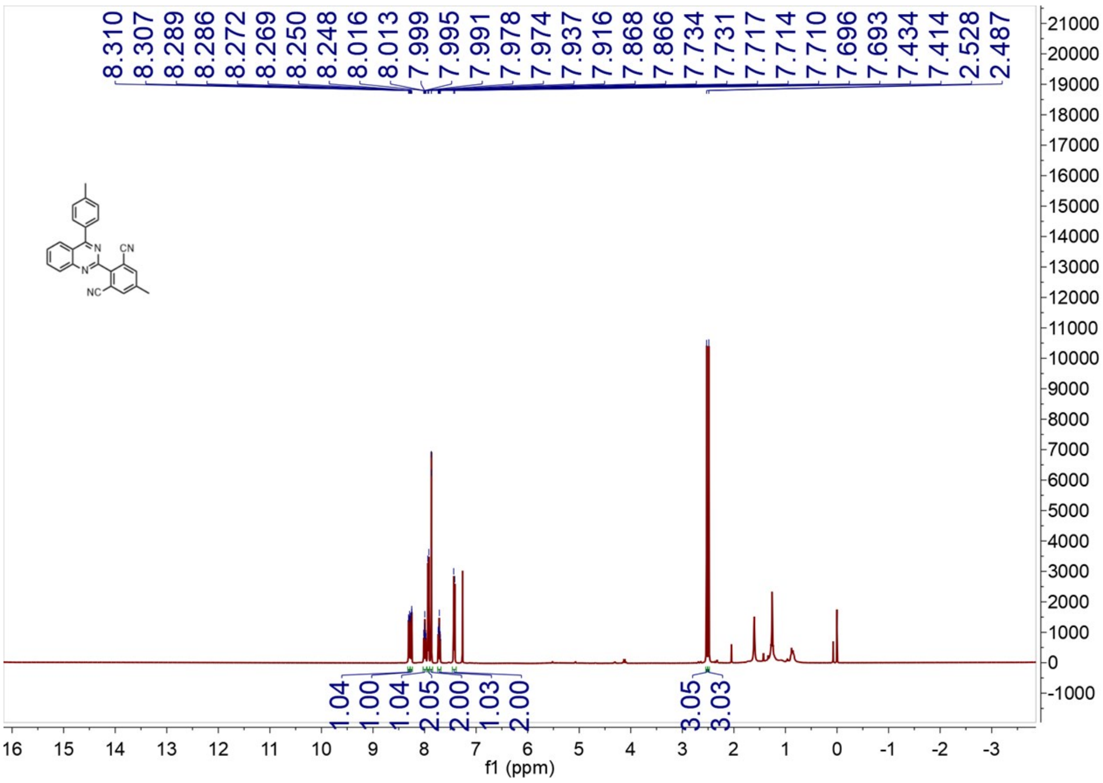

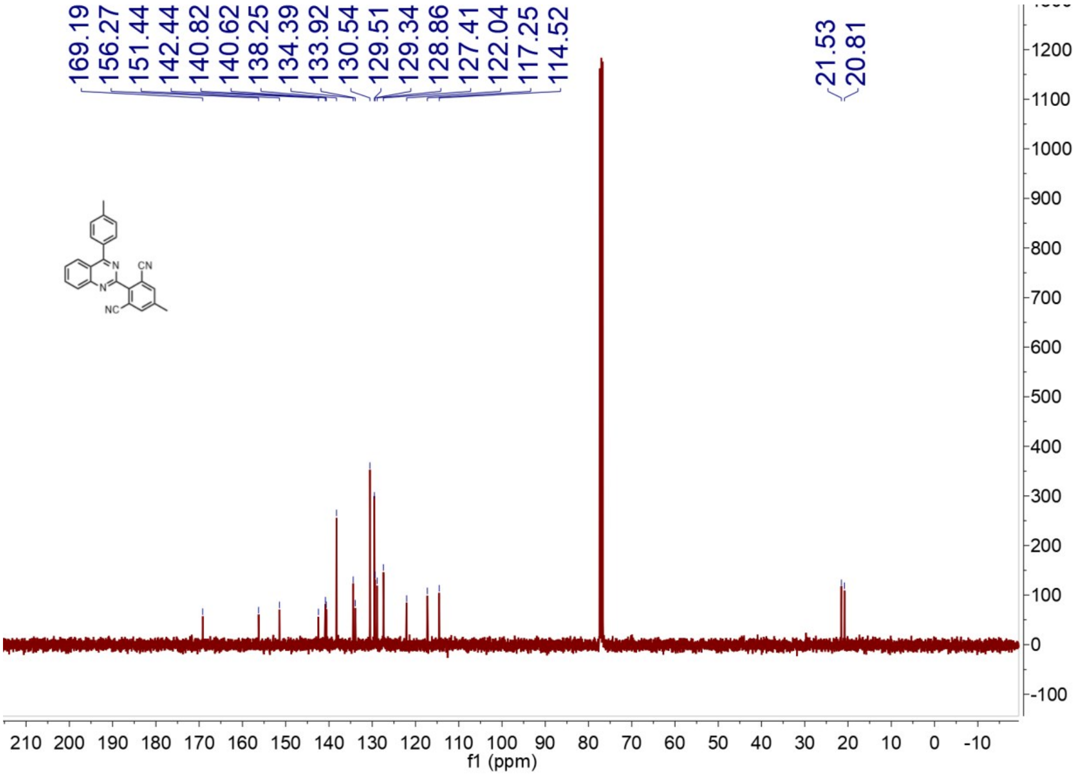

2-(2, 6-dicyano-4-methoxyphenyl)-4-(p-tolyl)quinazoline (4b)

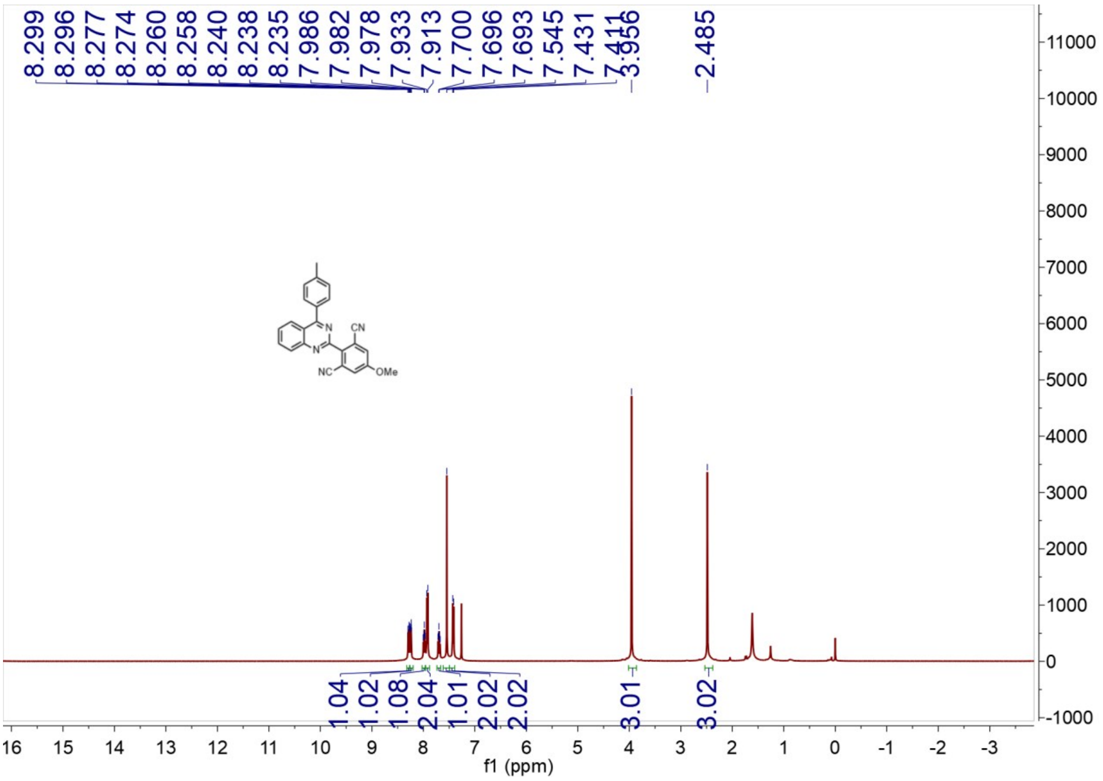
8.299 8.296 8.277 8.274 8.260 8.258 8.240 8.238 8.235 7.986 7.982 7.978 7.933 7.913 7.700 7.696 7.693 7.545 7.431 7.411 3.956
2.485
1.04 1.02 1.08 2.04 1.01 2.02 2.02
3.01
3.02
CN
NC
OMe
N
f1 (ppm)

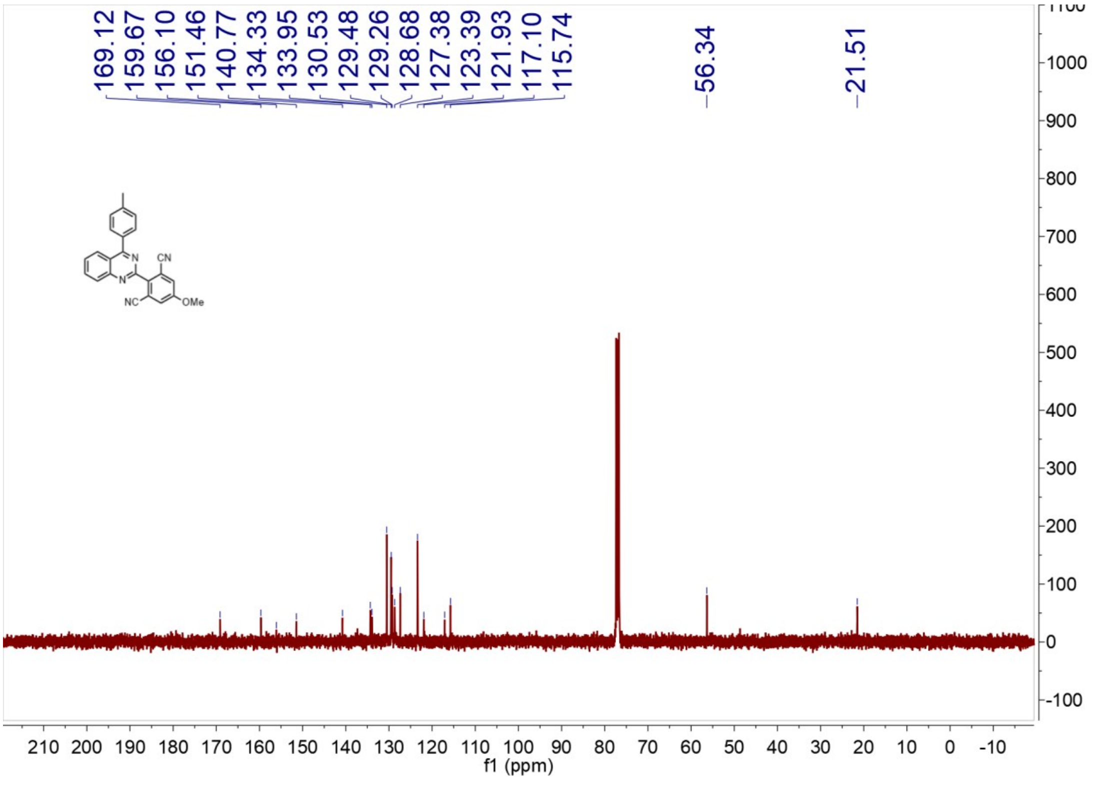
169.12 159.67 156.10 151.46 140.77 134.33 133.95 130.53 129.48 129.26 128.68 127.38 123.39 121.93 117.10 115.74
56.34
21.51
CN
NC
OMe
N
f1 (ppm)

2-(2, 6-dicyanophenyl)-4-(p-tolyl)quinazoline (4c)

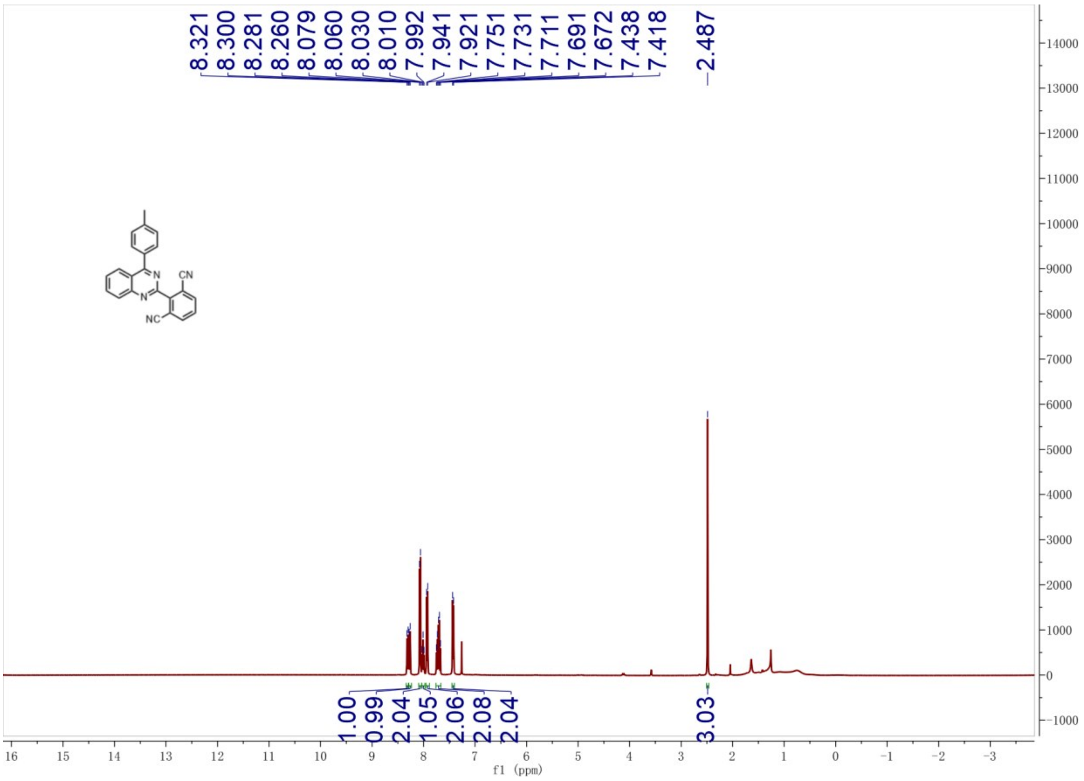

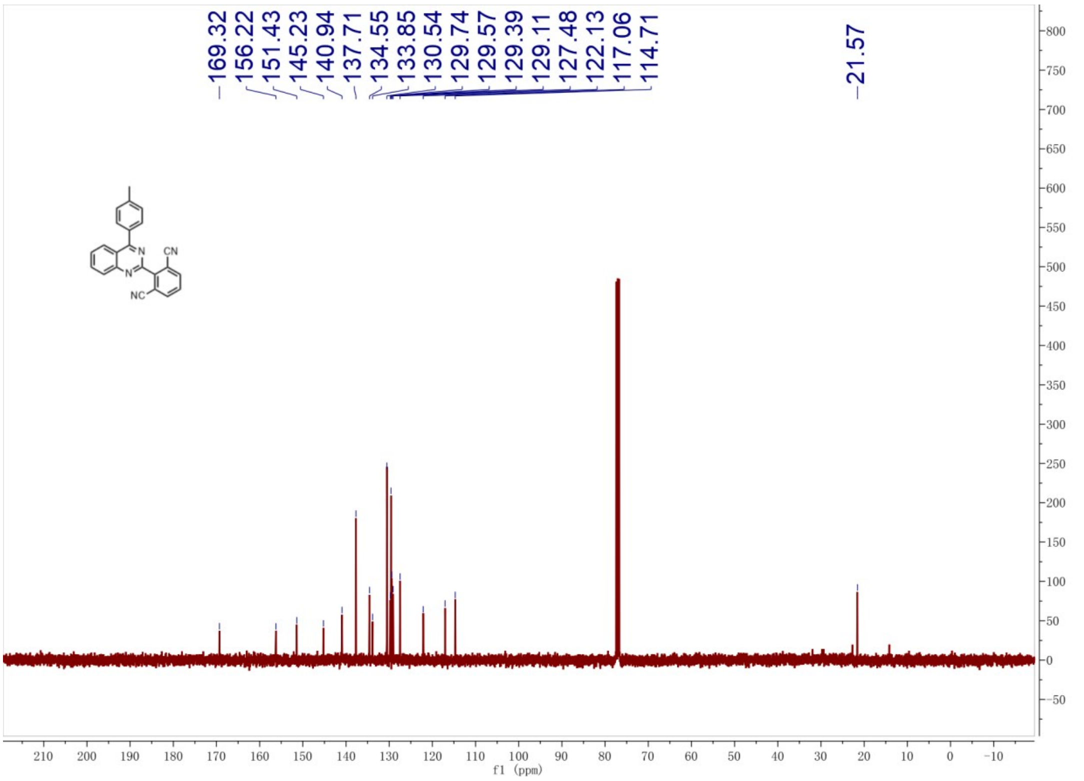

2-(2, 6-dicyano-4-chlorophenyl)-4-(p-tolyl)quinazoline (4d)

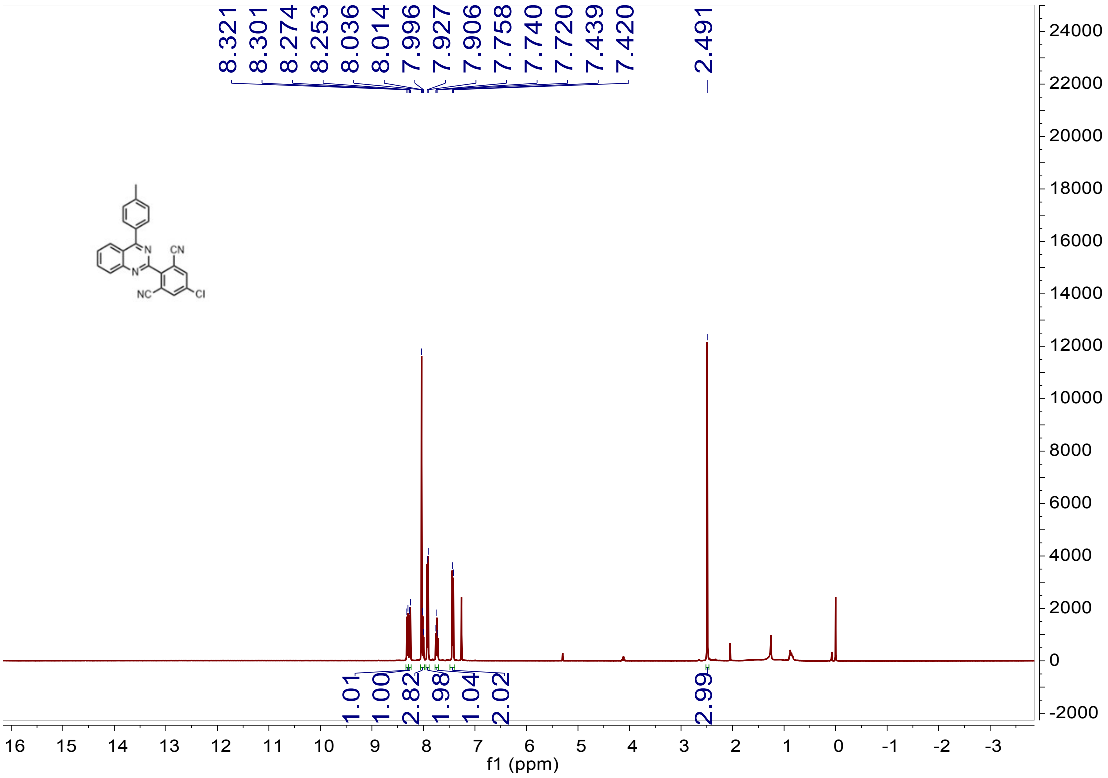

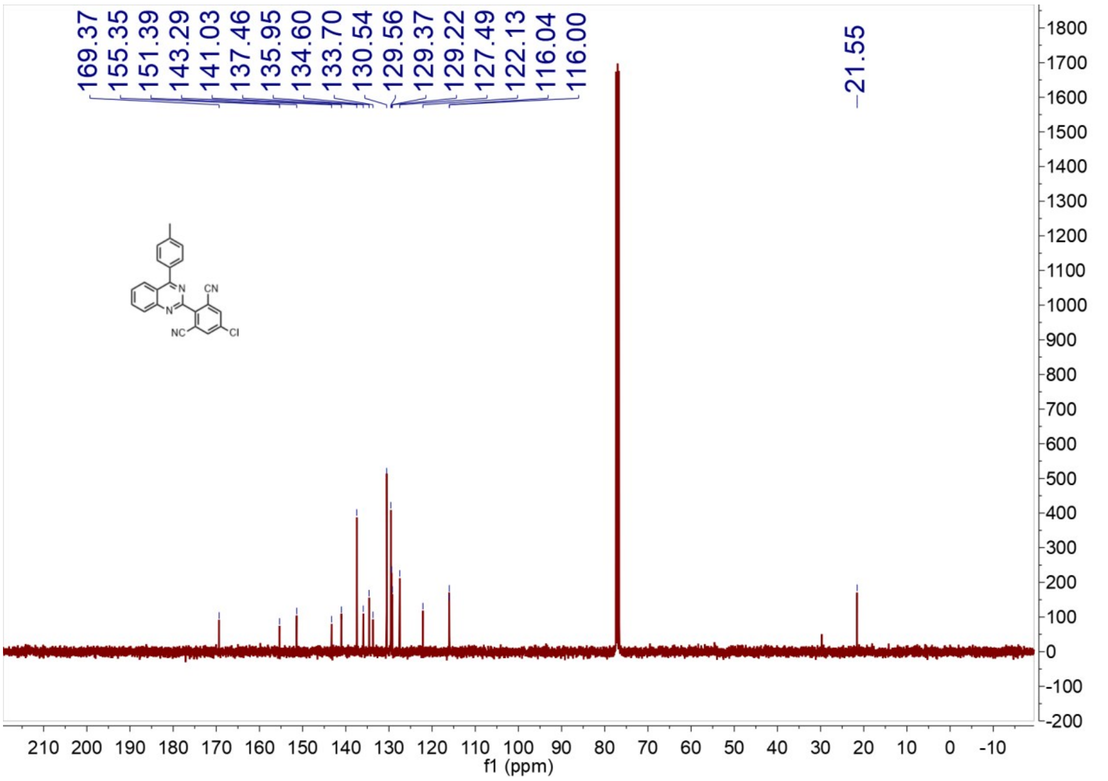

2-(2,6-dicyano-4-fluorophenyl)-4-(p-tolyl)quinazoline(4e)

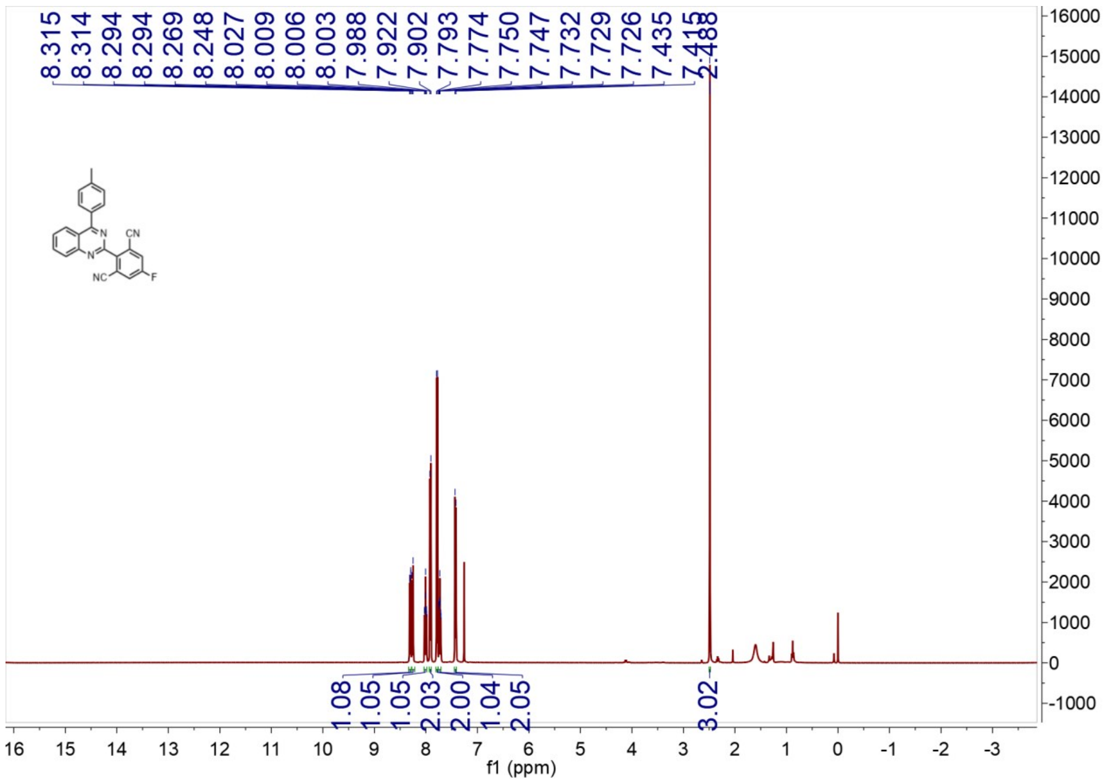

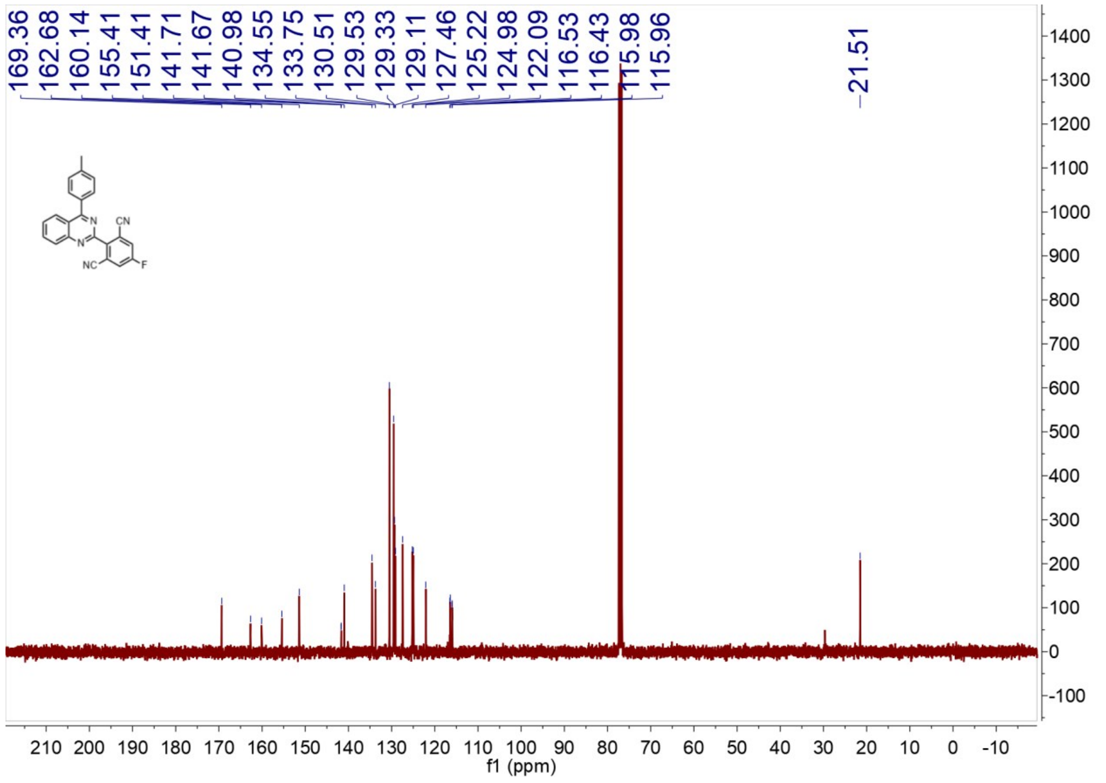

2-(2,6-dicyano-4-methylphenyl)-4-(4-methoxyphenyl)quinazoline(4f)

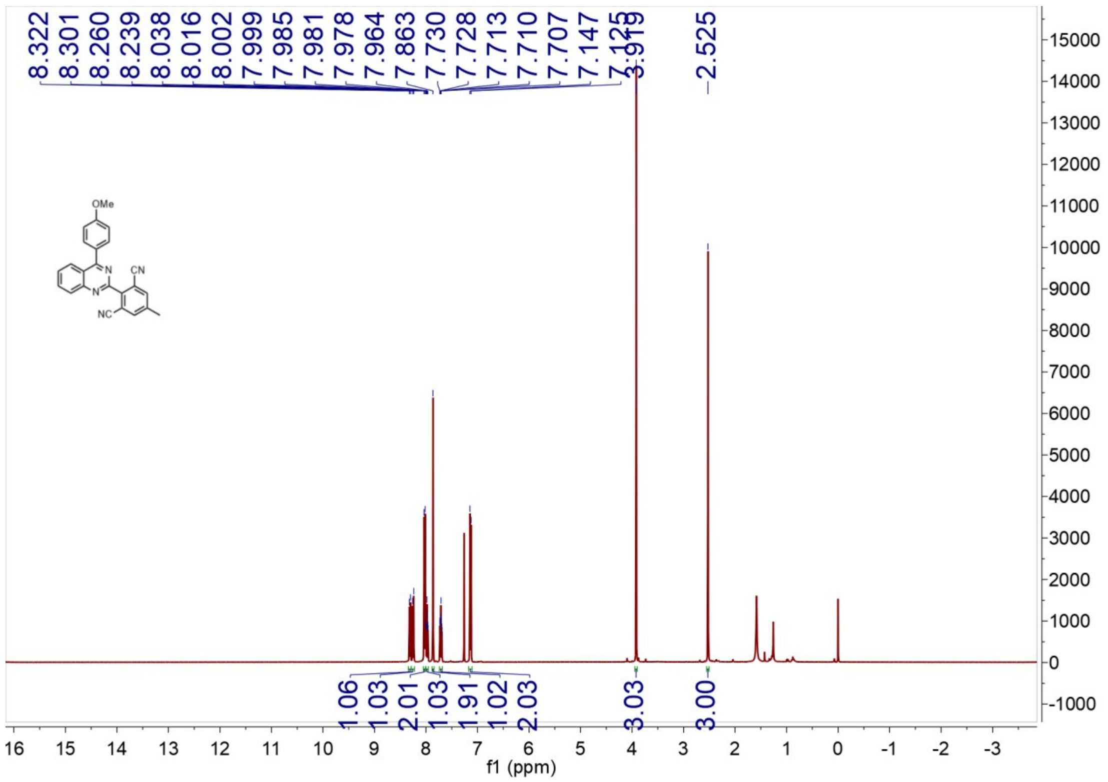

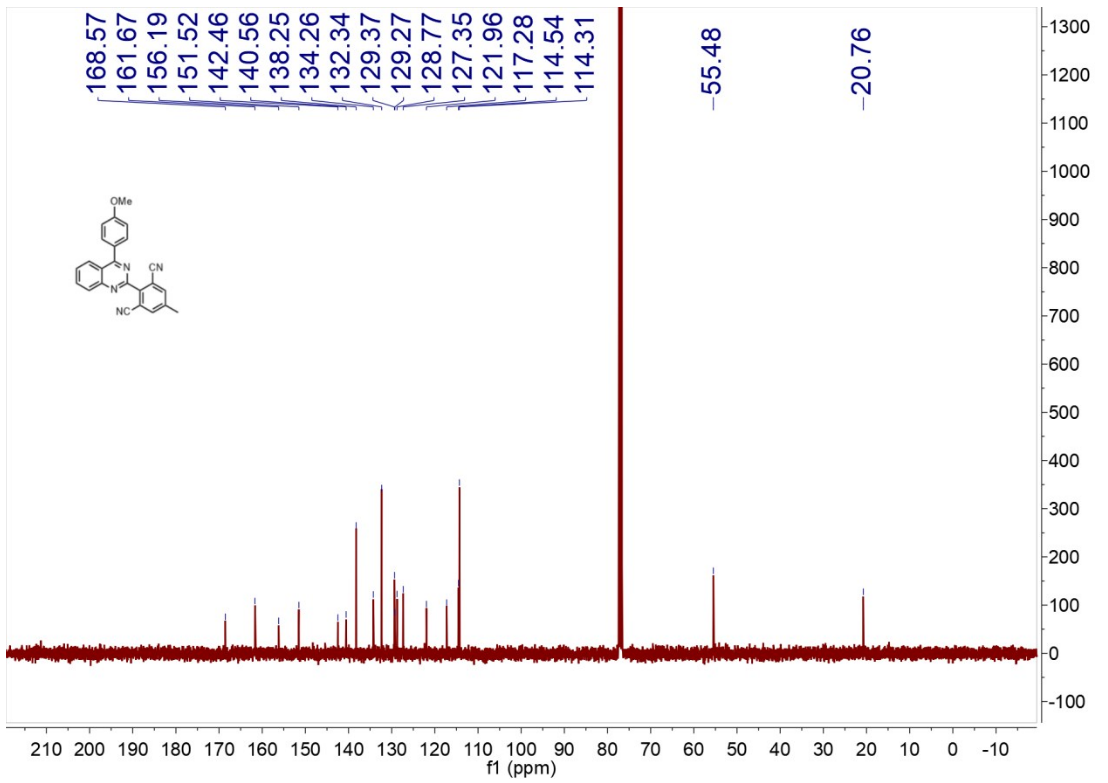

2-(2,6-dicyano-4-methylphenyl)-4-(4-chlorophenyl)quinazoline(4g)

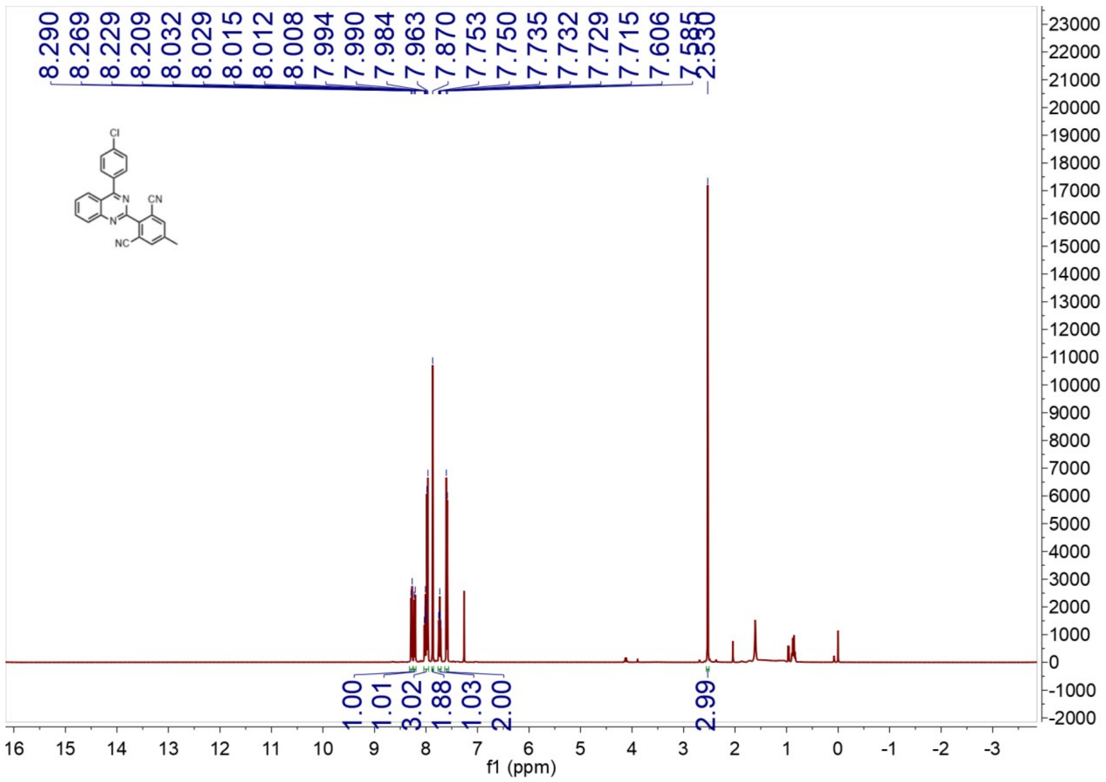

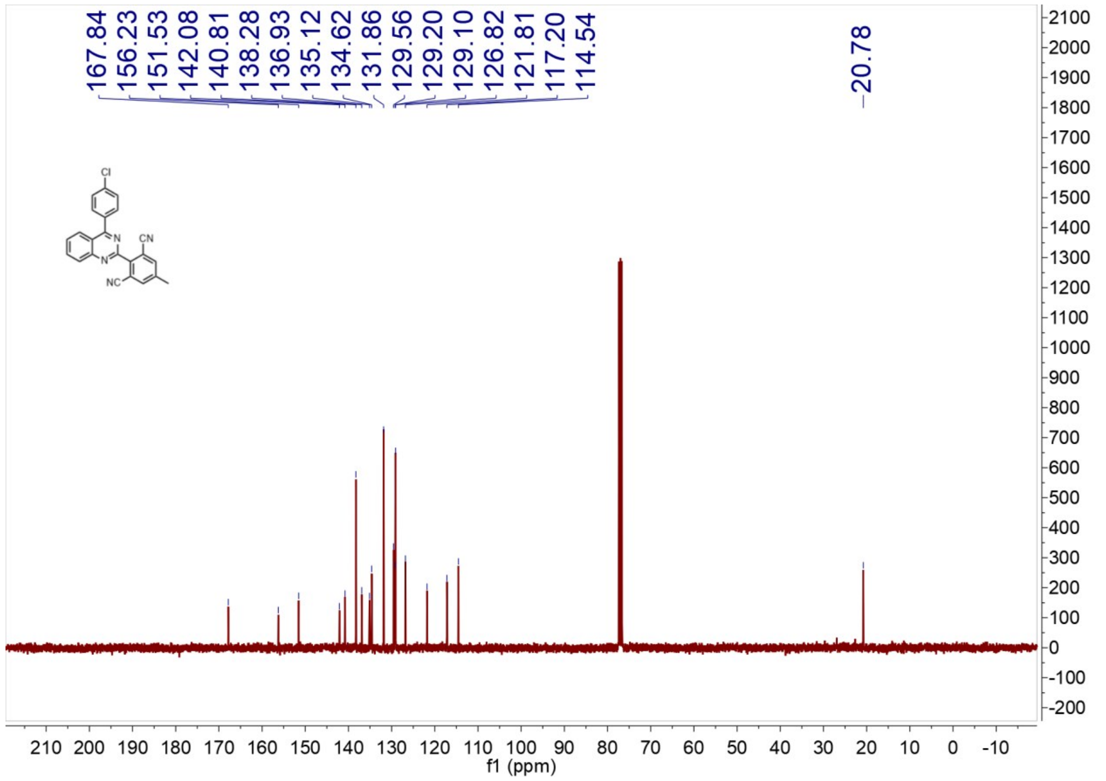

2-(2,6-dicyano-4-methylphenyl)-4-(4-fluorophenyl)quinazoline(4h)

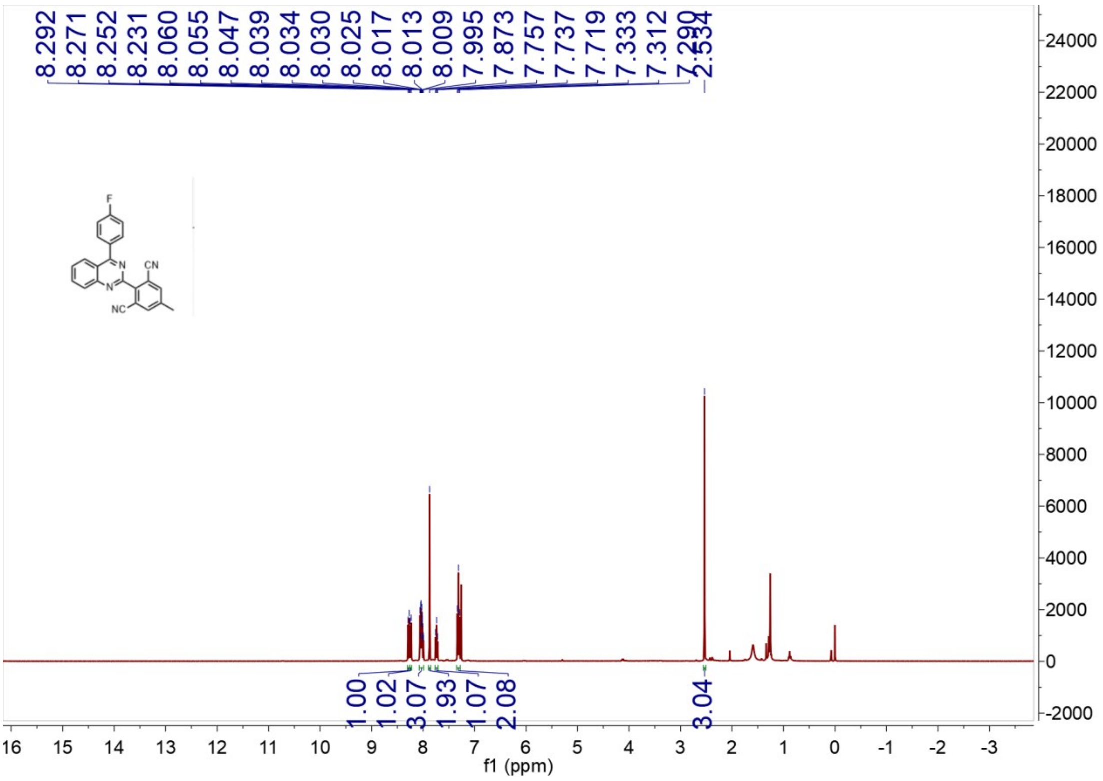

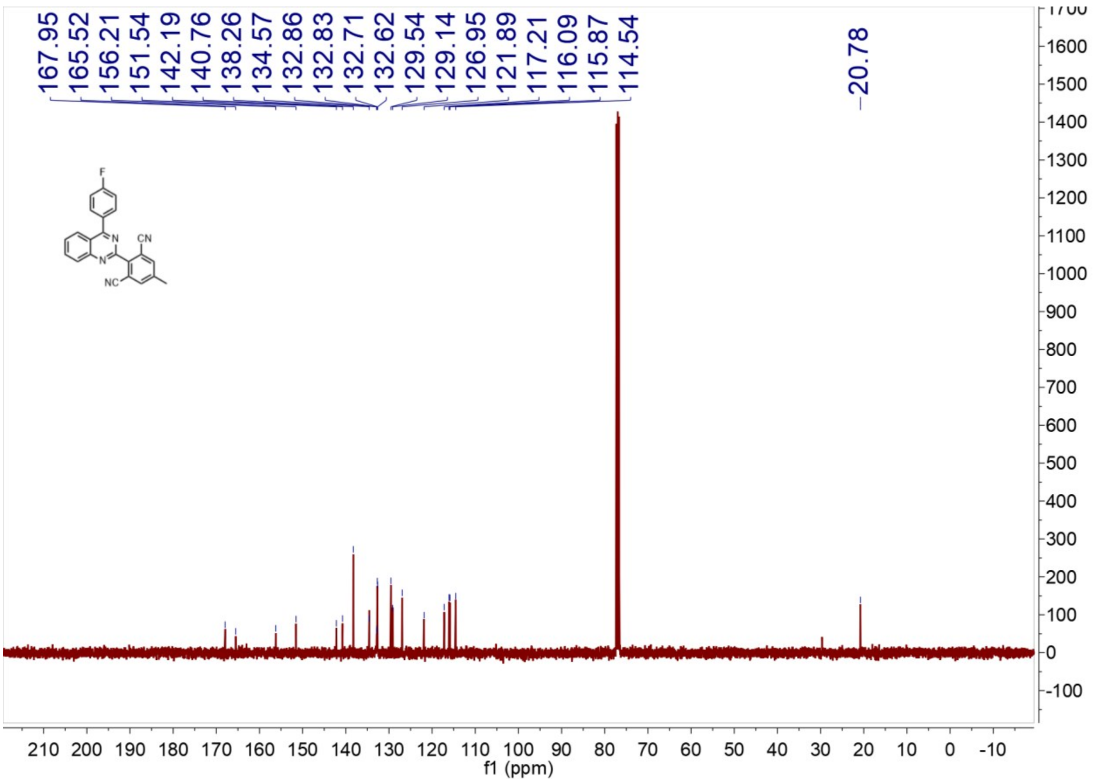

2-(2,6-dicyano-4-methylphenyl)-4-(p-tolyl)-6-methoxylquinazoline(4i)

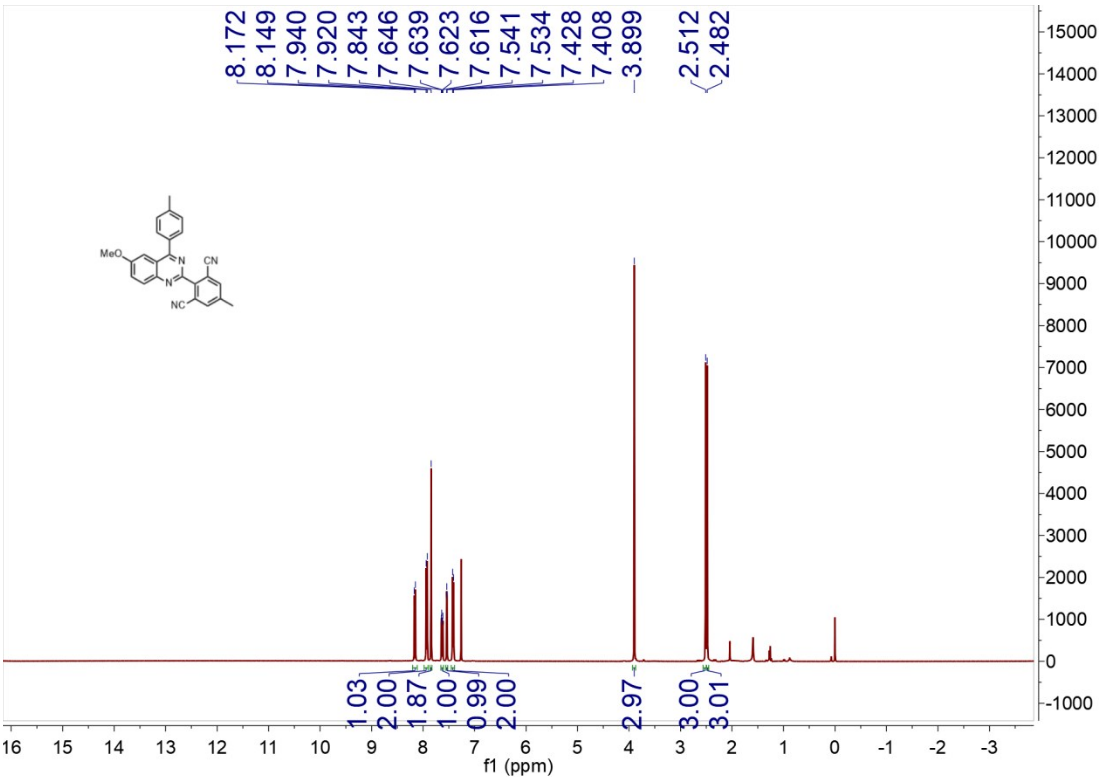

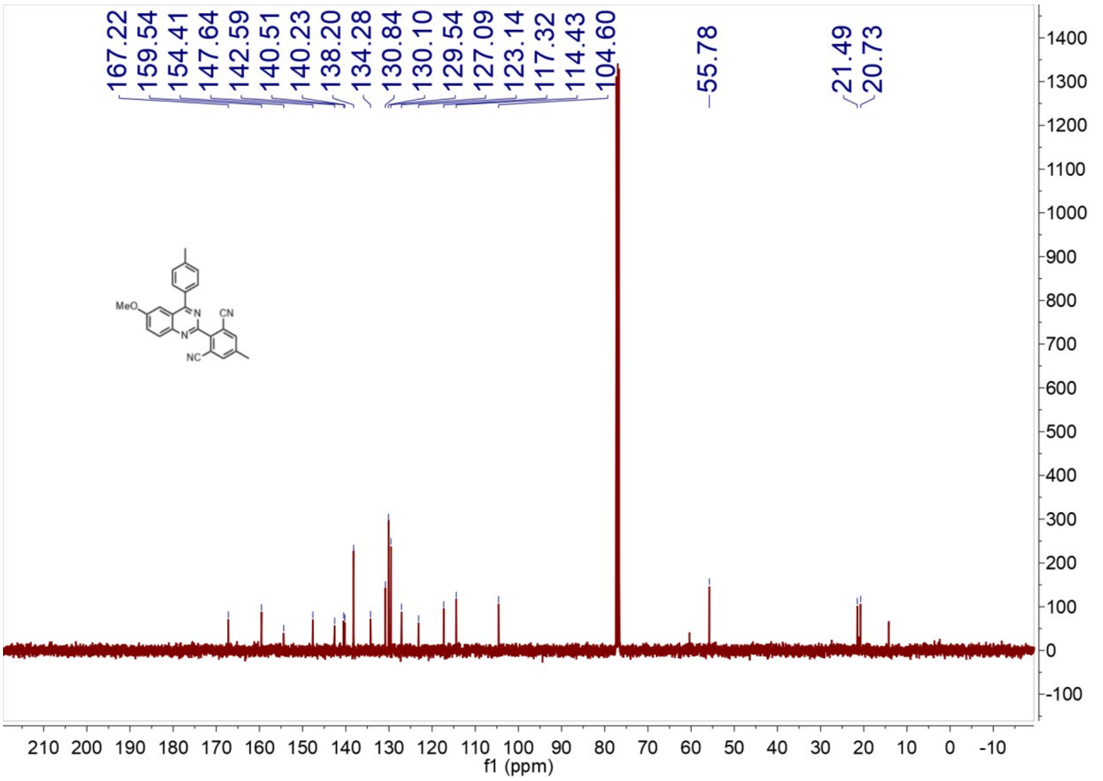

2-(2, 6-dicyano-3-methylphenyl)-4-(p-tolyl)quinazoline (4j)

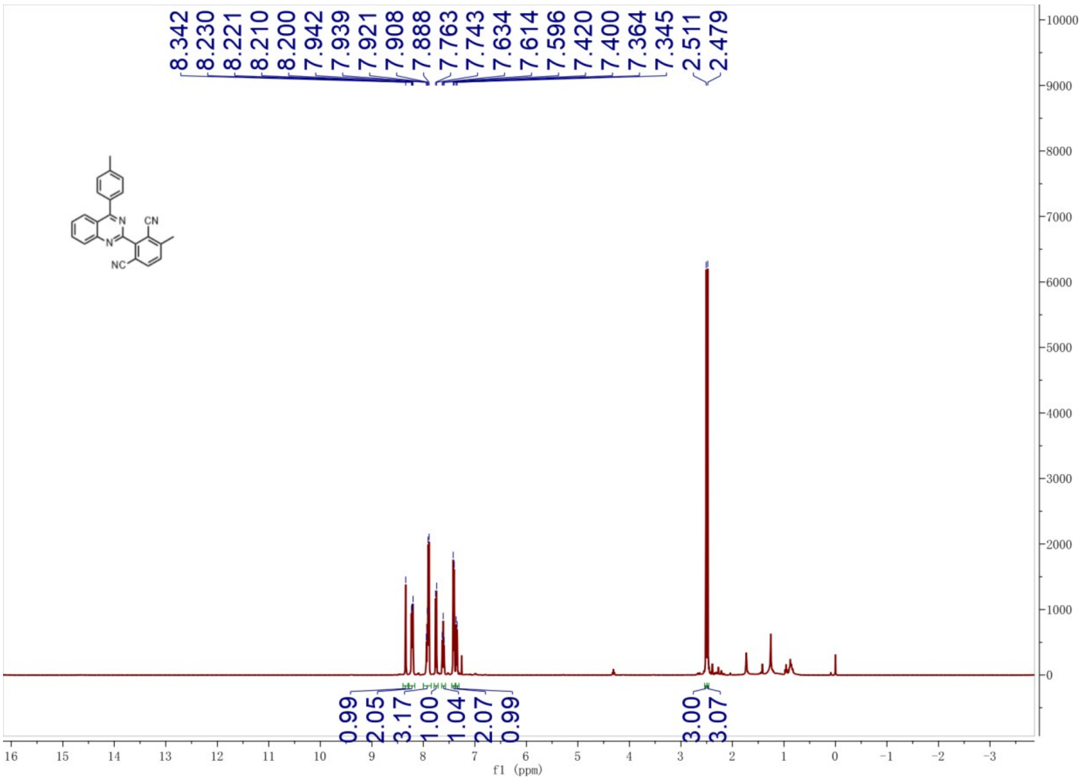

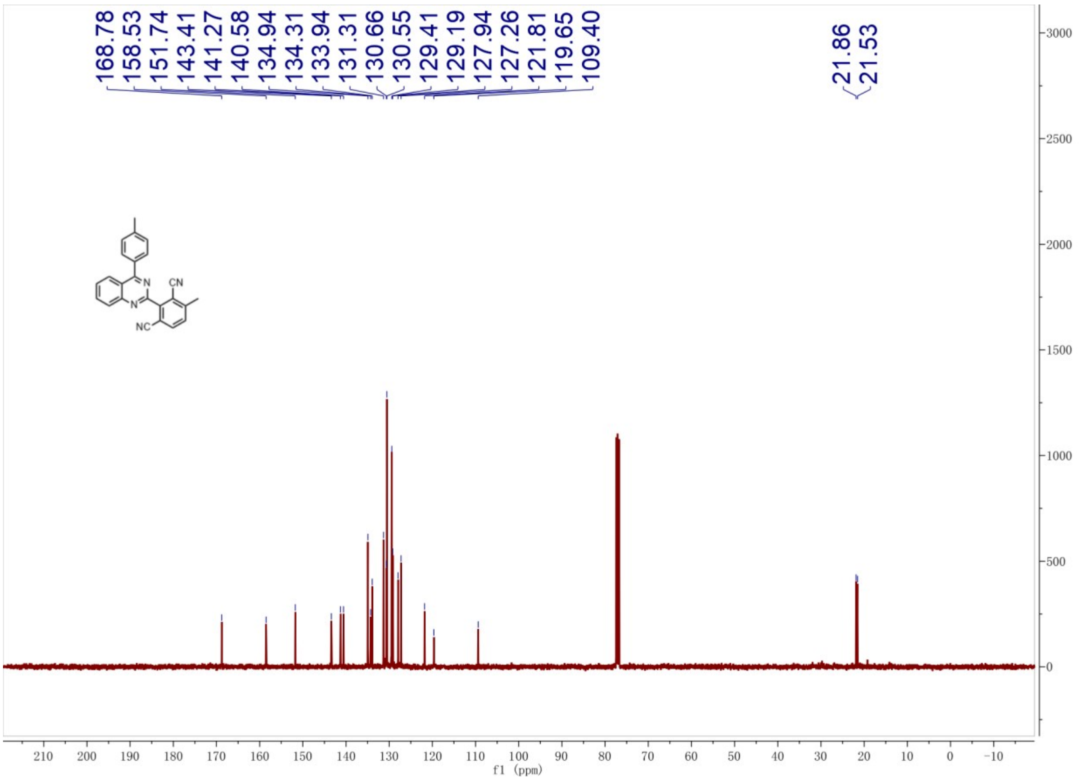

2-(2, 6-dicyano-3-chlorophenyl)-4-(p-tolyl)quinazoline (4k)

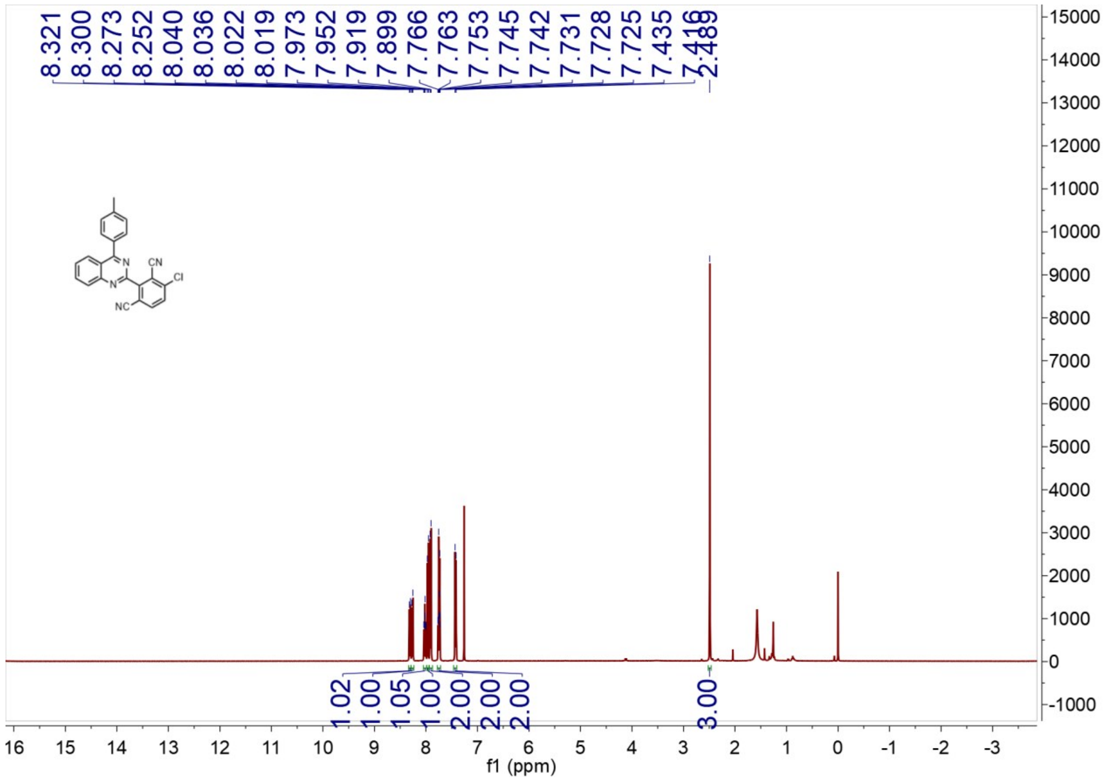
8.321
8.300
8.273
8.252
8.040
8.036
8.022
8.019
7.973
7.952
7.919
7.899
7.766
7.763
7.753
7.745
7.742
7.731
7.728
7.725
7.435
7.416
2.489
CN
N
Cl
NC
1.02
1.00
1.05
1.00
2.00
2.00
2.00
3.00
f1 (ppm)

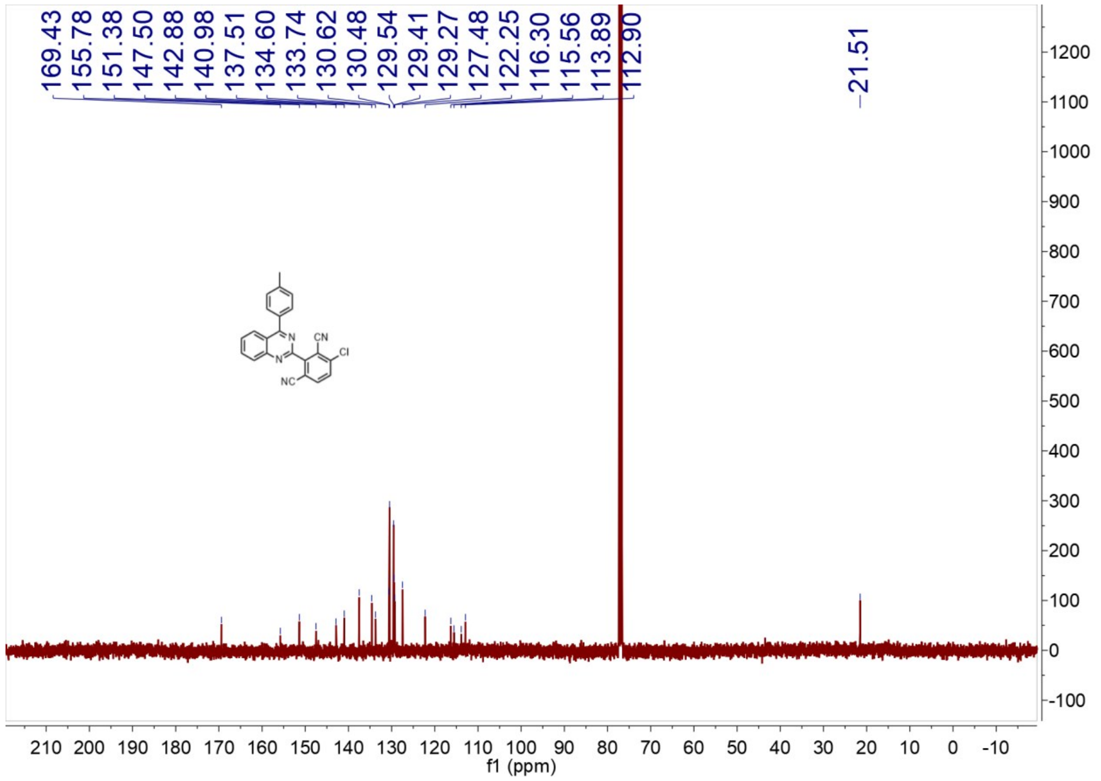
169.43
155.78
151.38
147.50
142.88
140.98
137.51
134.60
133.74
130.62
130.48
129.54
129.41
129.27
127.48
122.25
116.30
115.56
113.89
112.90
21.51
CN
N
Cl
NC
f1 (ppm)

2-(Pyridin-2-yl)isophthalonitrile (4l)

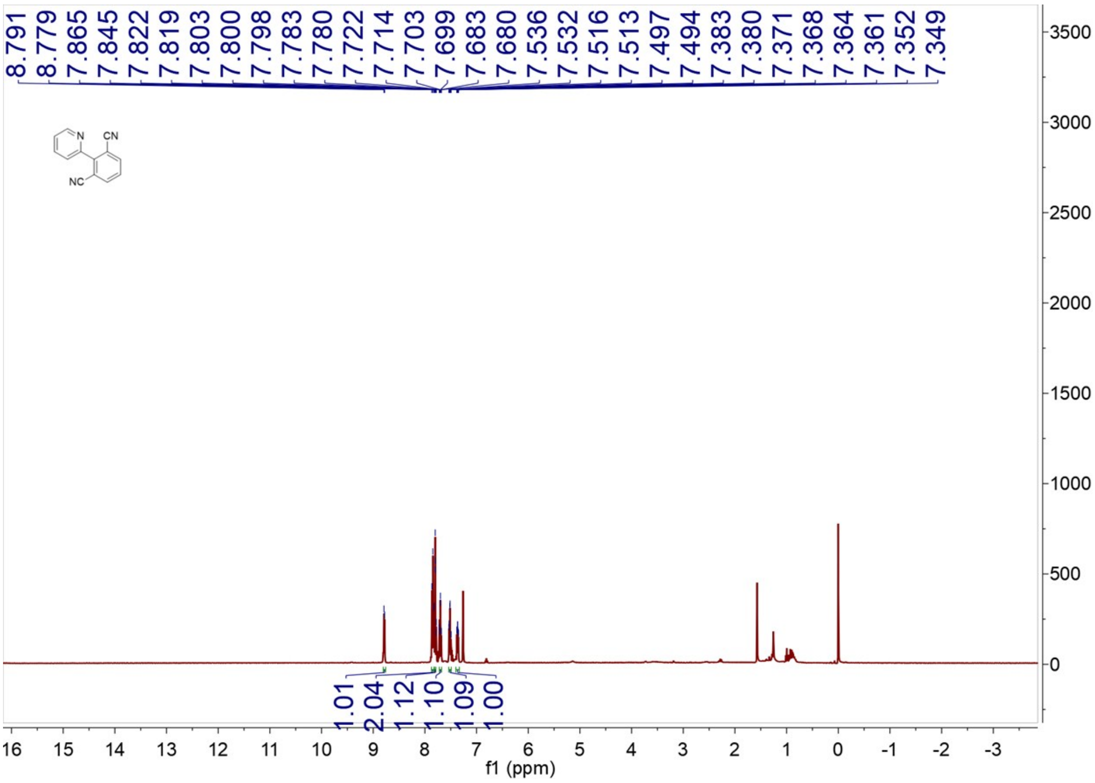

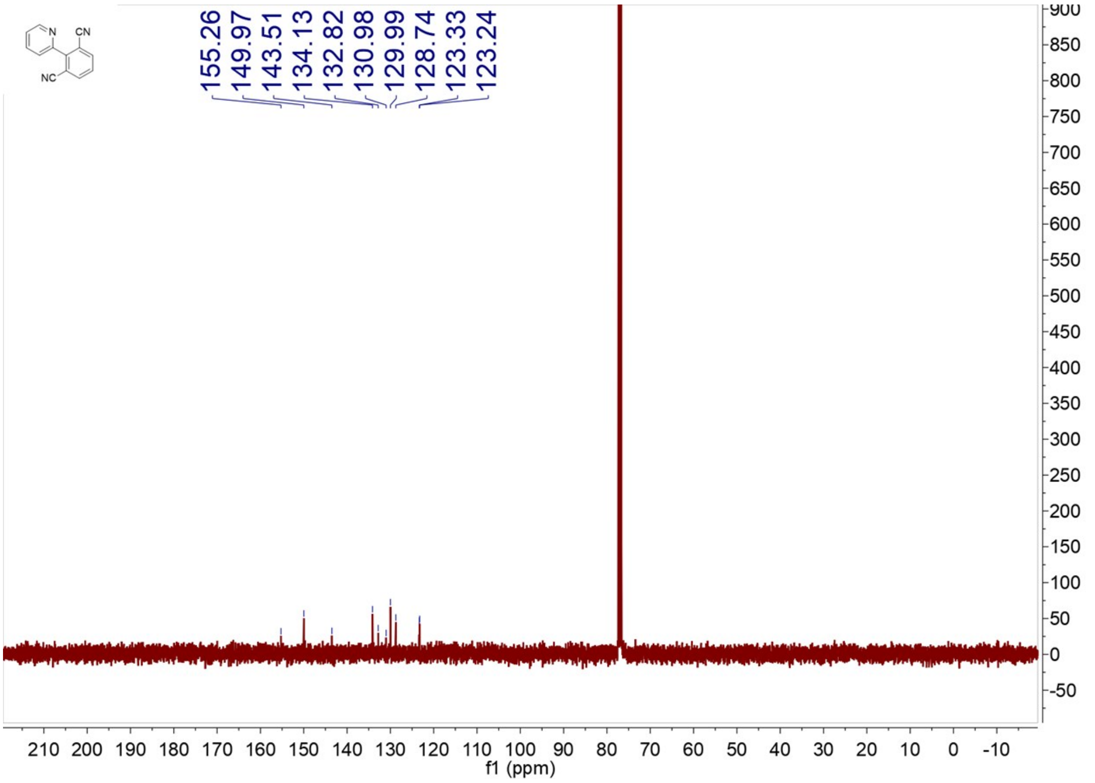

Supplement: RA-009-C9RA02979F-s001 [file RA-009-C9RA02979F-s001.pdf]
